# Autoregulation of *mazEF* expression underlies growth heterogeneity in bacterial populations

**DOI:** 10.1093/nar/gky079

**Published:** 2018-02-08

**Authors:** Nela Nikolic, Tobias Bergmiller, Alexandra Vandervelde, Tanino G Albanese, Lendert Gelens, Isabella Moll

**Affiliations:** 1 Department of Microbiology, Immunobiology and Genetics, Max F. Perutz Laboratories, University of Vienna, Vienna BioCenter (VBC), 1030 Vienna, Austria; 2 Institute of Science and Technology Austria (IST Austria), 3400 Klosterneuburg, Austria; 3 Laboratory of Dynamics in Biological Systems, Department of Cellular and Molecular Medicine, University of Leuven, 3000 Leuven, Belgium

## Abstract

The MazF toxin sequence-specifically cleaves single-stranded RNA upon various stressful conditions, and it is activated as a part of the *mazEF* toxin–antitoxin module in *Escherichia coli*. Although autoregulation of *mazEF* expression through the MazE antitoxin-dependent transcriptional repression has been biochemically characterized, less is known about post-transcriptional autoregulation, as well as how both of these autoregulatory features affect growth of single cells during conditions that promote MazF production. Here, we demonstrate post-transcriptional autoregulation of *mazF* expression dynamics by MazF cleaving its own transcript. Single-cell analyses of bacterial populations during ectopic MazF production indicated that two-level autoregulation of *mazEF* expression influences cell-to-cell growth rate heterogeneity. The increase in growth rate heterogeneity is governed by the MazE antitoxin, and tuned by the MazF-dependent *mazF* mRNA cleavage. Also, both autoregulatory features grant rapid exit from the stress caused by *mazF* overexpression. Time-lapse microscopy revealed that MazF-mediated cleavage of *mazF* mRNA leads to increased temporal variability in length of individual cells during ectopic *mazF* overexpression, as explained by a stochastic model indicating that *mazEF* mRNA cleavage underlies temporal fluctuations in MazF levels during stress.

## INTRODUCTION

MazF is the toxin component of the *mazEF* toxin–antitoxin (TA) system, which is a type II TA locus (1–3). The *mazF* gene encodes an endoribonuclease that sequence-specifically cleaves single-stranded RNA at ACA sites in *Escherichia coli* (4), and *mazE* encodes the unstable antitoxin that is co-expressed with *mazF*. Under non-stressful conditions, MazF is inactivated by a sandwich-like complex consisting of two homodimers of MazF and one homodimer of MazE (5). Upon stress, the activity of the proteases ClpAP (6,7) and Lon (8) is increased, resulting in fast degradation of MazE thereby allowing MazF to exert its function, which leads to reduction in overall translation and consequently inhibition of bacterial growth (9). In addition, toxin activation impairs the ability to form colonies, which is predominantly caused by the formation of viable but non-culturable cells, and not by cell death (1). The *mazEF* system can be triggered by a variety of stress conditions, such as starvation, heat shock and DNA damage (10,11). Moreover, MazF-mediated RNA cleavage occurs in the presence of antibiotics that are general inhibitors of transcription (rifampicin) or translation (chloramphenicol and spectinomycin) (12).

Expression of the *mazEF* module is controlled by a multifaceted transcriptional negative autoregulation system. The MazE antitoxin and the MazE–MazF complex repress transcription of the *mazEF* operon during non-stressful conditions (13). Upon stress the MazE antitoxin is degraded (6,8), which results in de-repression of *mazEF* transcription. Hence, the molar ratio between toxin and antitoxin within a cell dictates the level of *mazEF* transcription through a mechanism called ‘conditional cooperativity’ (14). Described for several TA modules, conditional cooperativity prevents random toxin activation in conditions without stress, and facilitates fast recovery from translational inhibition (15–19). Furthermore, since the *mazF* mRNA comprises several ACA sites, it has been suggested that MazF cleaves and degrades its own transcript *in vitro* (4). However, experimental evidence supporting this hypothesis has not been reported, and the physiological role of this additional post-transcriptional autoregulatory feature possibly affecting TA expression dynamics has not been investigated yet.

Complex networks harboring autoregulatory features and protein-protein interactions are prone to generate cell-to-cell variation in gene expression levels and phenotypic heterogeneity (20–22). Generally, phenotypic heterogeneity in populations of genetically identical bacterial cells can arise independently of genetic or environmental differences (23,24). A number of studies revealed that activation of a toxin promotes phenotypic variation in bacterial populations, which can be measured as heterogeneity in growth rate (25,26), cell size (27), and gene expression (28,29). We hypothesized that dynamic regulation of TA expression could account for population heterogeneity, and control and optimize entry and exit from growth arrest caused by the toxin activation.

Here, we investigate how the autoregulation of *mazEF* expression at the transcriptional and post-transcriptional level affects growth of *E. coli* cells and populations. Although the autoregulation of *mazEF* expression has been biochemically characterized at the transcriptional level (13), we aimed to elucidate the consequences of this transcriptional repression on growth behavior of single cells. In addition, we biochemically verified the previously hypothesized notion that MazF cleaves its own transcript *in vivo* (4,30), and studied the impact of this post-transcriptional autoregulation on heterogeneity between single cells by flow cytometry, as well as on variability within individual cells over time by microfluidic-based time-lapse microscopy and stochastic modeling.

## MATERIALS AND METHODS

### Strains

The *E. coli* strains K-12 MG1655 (31) and BW27784 (32) and their derivatives were used in this study (Supplementary Table S1). As a main reporter system, we used a chromosomally integrated reporter for a constitutively expressed gene, i.e. the phage λ promoter drives expression of *mCherry* (33). A variant of the fast-folding Emerald GFP was used as an additional reporter gene, Em*gfp*ΔACA (34), called ‘*gfp*’ throughout the manuscript. The Em*gfp*ΔACA amino acid sequence corresponds to the wild-type sequence of Emerald GFP, however the nucleotide sequence was modified such that all ACA sites were substituted. This sequence modification prevents MazF-mediated sequence-specific mRNA cleavage inside the coding region of *gfp*. Arabinose-inducible *mazF* was expressed from two sources: either based on a medium-copy plasmid pBAD-*mazF* (20–30 copies of P_BAD_-*mazF* and of AraC (35)) for excessive *mazF* overexpression (7,36), or as a chromosomally integrated system (a chromosomal copy of P_BAD_-*mazF* and a native copy of the transcriptional regulator AraC) for mild level of expression. Sequences of the constructs are reported in Supplementary Information – Supplementary Methods.

### Conditions

Cultures were grown in rich media with 1× M9 salts, 1 mM MgSO_4_, 0.1 mM CaCl_2_, 0.5% casamino acids (Fluka), 10 mM maltose. The antibiotics were added for plasmid maintenance in the following final concentrations: 100 μg/ml ampicillin, 15 μg/ml chloramphenicol, 50 μg/ml kanamycin. In general, frozen glycerol clones were first streaked on LB agar. A single colony was inoculated overnight in 4 ml of rich defined media, incubated at 37°C with shaking at 165 rpm. On the following day, the cultures were diluted 1:1000 into fresh rich media (OD_600_ ∼ 0.007), and analyzed after 2 h 15 min when they were in the exponential phase. Each replicate culture was then split into tubes where one served as a control, and different stressors or inducers of *mazF* expression were added in other tubes (4 ml in each tube). The cultures were analyzed by flow cytometry after the time period indicated in the Results section. For the majority of experiments regarding constitutive reporter genes, the cultures were analyzed in two time points: 2 h (t1) and 5.5–6.5 h (t2) after arabinose induction.

### Growth measurements

Bacteria were grown in 96-well plates incubated at 37°C with shaking, and growth was recorded every 10 min in a Synergy H1 plate-reader (BioTek Instruments, Inc.) as absorbance at 600 nm (*A*_600_). Maximum growth rate was defined as the maximum value of slopes calculated as ln-transformed measurements over five time points, i.e. 40 min. Additionally, bacterial growth was monitored by measuring optical density (OD) at 600 nm with a spectrophotometer at specific time points.

### Flow cytometry

#### FACS Calibur

Flow cytometer FACS Calibur (BD, CA, USA) is equipped with argon laser with excitation at 488 nm. For each sample 100 000 events were acquired at low speed (generally, 1800–2000 events/s) and measured with the following ‘height’ settings—FSC-H (forward scatter): E01, SSC-H (side scatter): 349 V, FL1-H: 813 V; log mode; primary threshold on SSC.

#### LSR Fortessa

Flow cytometer LSR Fortessa (BD, CA, USA) is equipped with lasers with excitation at 488 nm (blue laser) and 561 nm (yellow–green laser), required to detected GFP fluorescence (BP filter 530/30 nm) and mCherry fluorescence (BP filter 610/20 nm), respectively. For each sample 50 000 events were acquired at low speed (generally, <1000 events/s) and measured with the following ‘height’ settings—FSC-H (forward scatter): 380 V, SSC-H (side scatter): 220 V, FITC-H: 430 V (GFP detection), PE-Texas Red-H: 590 V (mCherry detection); log mode; compensation FITC-PE Texas Red 0.2; threshold: SSC at 200 V and FSC at 200 V.

### Time-lapse microscopy

All images were acquired with a Nikon Ti-Eclipse microscope equipped with a Lumencore light source and enclosed into a custom-made incubation box with a temperature controller (Reinach, Life Imaging Services, Switzerland). The microscope was equipped with a perfect focus system (PFS), and images were acquired every 5 min using a 100 × 1.4 NA oil immersion objective lens and a cooled Hamamatsu EMCCD C9100-02 with a pixel size of 0.08 μm/pixel connected to the microscope with a 0.7× c-mount adapter. To image mCherry fluorescent protein, we used the green LED (549 ± 15 nm) with an intensity of 320 mW and an exposure time of 250 ms. The emission filter was from Chroma (TexasRed HYQ LP 596, BP641/75).

### Image analysis

Microscopy images were analyzed with the Matlab-based package *Schnitzcells* (37). We analyzed single-cell growth rate, mCherry fluorescence, cell length and cell cycle duration of a bottom cell in each microfluidic channel. Single-cell growth was measured as the cell elongation rate and computed by exponential fitting the plot of cell length versus time, measured from cell birth until division (38). Cell length and mCherry fluorescence were measured just before cell division event. Normalized fluorescence was defined as total fluorescence divided by the cell area (37), and it is proportional to the concentration of fluorescent protein molecules in the cell.

### Northern blot analysis

To verify MazF ability to cleave its own mRNA, single colonies of MG1655 harboring either pBAD-*mazF* or pBAD-*mazF*ΔACA were inoculated and grown overnight in selective LB media. Plasmid pBAD-*mazF*_E24A_ in the MG1655Δ*mazF* background was used as a control for mRNA stability and MazF-independent cleavage. Ectopically expressed MazF_E24A_ protein is enzymatically inactive, and thus cannot cleave RNA (39). Strain BW27784 without any plasmid was used as an additional negative control.

Overnight cultures were diluted in 50 ml of LB medium supplemented with chloramphenicol, and their growth was monitored by measuring optical density at 600 nm (OD_600_). At OD_600_ = 0.2–0.3, *mazF* expression was induced by addition of l-arabinose to the final concentration of 0.2%. 30 min (*mazF* mRNA detection) or 60 min (*mCherry* mRNA detection) after induction, the cells were harvested and the pellet was frozen in liquid nitrogen. RNA was extracted using TRIzol^®^ reagent (Thermo Scientific) as described by the manufacturer protocol. Upon isopropanol precipitation, 7.5 μg of total RNA extract was separated in a denaturing polyacrylamide gel (5% or 10%), and transferred to an Amersham Hybond-XL (GE Healthcare) nylon membrane using the Trans-Blot Semi-Dry Transfer Cell (Bio-Rad). Prior hybridization, the membrane was UV crosslinked twice and stained with a solution of Methylene Blue (0.5 M sodium phosphate buffer pH 5.2, 0.04% Methylene Blue) for detection of the ladder (RiboRuler Low Range RNA Ladder, Thermo Scientific) and rRNA that serves as loading control. Subsequently, the membrane was hybridized to the [^32^P]-labeled DNA oligonucleotide, as listed in Supplementary Table S2.

To produce *in vitro* size markers, PCR products of defined length were amplified from pBAD-*mazF* using forward primer F47, and selected reverse primers designed to anneal on the *mazF* coding sequence at desired positions. As primer F47 contains T7 promoter sequence, the PCR product was employed as a template for *in vitro* transcription using T7 RNA Polymerase (Thermo Scientific). DNA template was digested with DNase I (Roche).

### Primer extension

For primer extension analysis, 5 pmol of 5′-[^32^P]-labeled L56 primer (Supplementary Table S2) were annealed to 6 μg of total RNA in Annealing Buffer (50 mM Tris, 60 mM NaCl, 10 mM DTT) by heating to 80°C for 3 min. Reactions were snap frozen and slowly thawed on ice. Primer extension reactions were performed using the AMV reverse transcriptase (Promega) by incubation at 42°C for 30 min in RT buffer (50 mM Tris, 60 mM NaCl, 10 mM DTT, 8 mM MgCl_2_, 1 mM dNTPs, 5 U AMV). For preparation of the ladder lanes, *mazF* coding sequence and its 5′ untranslated region (UTR) were first amplified from pBAD-*mazF* using suitable primers, and then sequenced using radiolabeled 5′-[^32^P]-labeled L56 primer and DNA Cycle Sequencing Kit (JENA Bioscience). The samples were separated on 8% polyacrylamide 7 M urea gel.

## RESULTS

### The antitoxin MazE controls growth reduction during *mazF* overexpression and enables fast exit from stress

**Figure 1.**
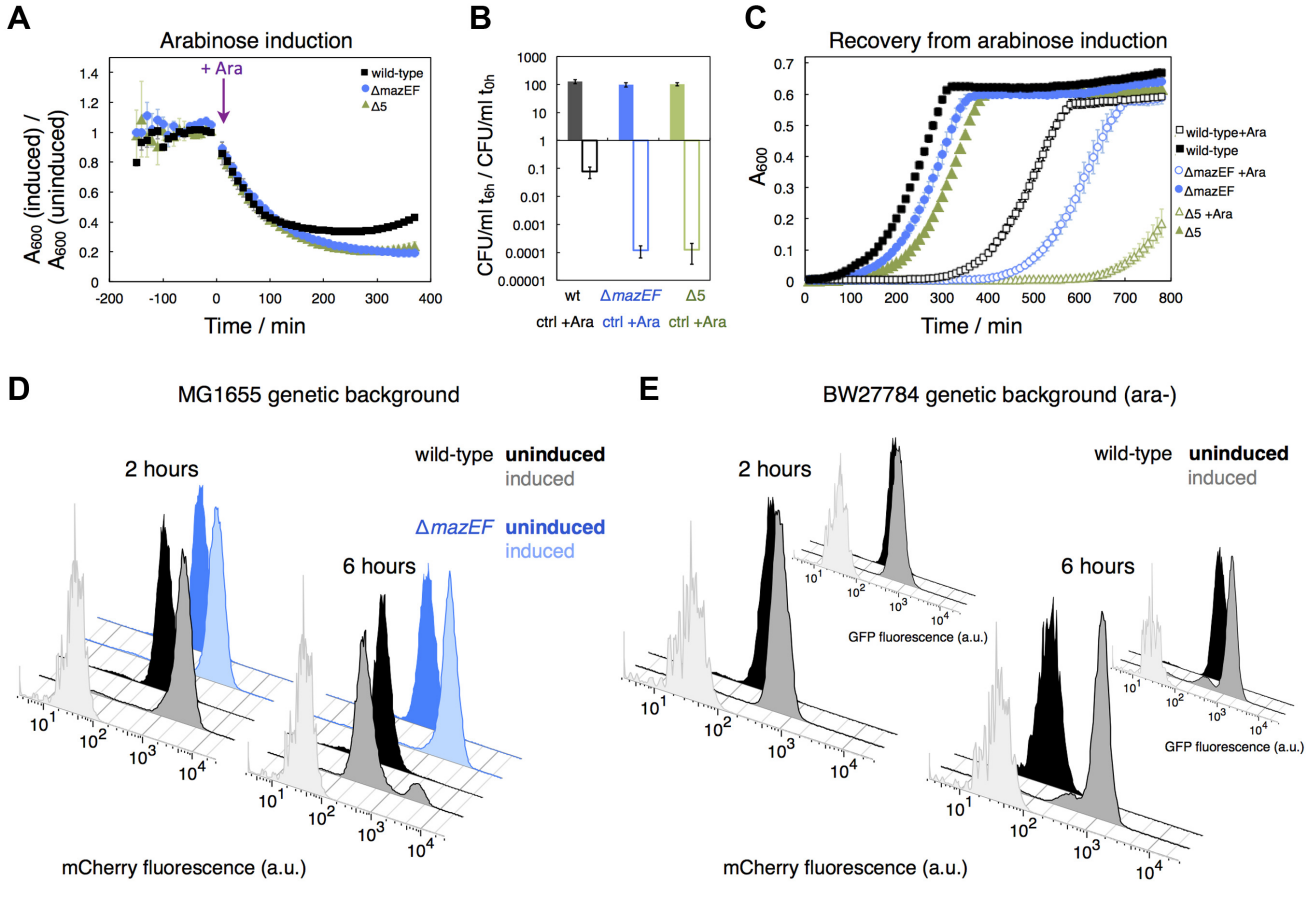
Influence of the antitoxin MazE on growth and constitutive reporter fluorescence during *mazF* overexpression. (**A**) We monitored growth of strain MG1655 (black), and isogenic strains Δ*mazEF* (blue) and Δ5 (five deleted TA loci) (green) harboring plasmids pBAD-*mazF* and pZS*-mCherry. 0.1% Ara was added to one half of exponentially growing cultures to induce *mazF* overexpression; the uninduced half served as a control. Growth was measured as absorbance at 600 nm (*A*_600_), and plotted is the ratio of the A_600_ measurements of induced cultures and uninduced (control) cultures, calculated per replicate. (**B**) We calculated the changes in CFU (colony-forming units) after 6 h in induced (empty bars) and uninduced cultures (filled bars) relative to the exponential cultures. Both mutant strains, Δ*mazEF* (empty blue bar) and Δ5 (empty green bar), exhibited lower CFU count compared to the wild-type strain (empty black bar) after 6 h of arabinose induction. (**C**) Six hours after arabinose induction, induced and uninduced cultures were washed, and allowed to recover in fresh media. We defined the period to exit the stress-phase as time required for bacterial populations to reach their maximum growth rate relative to the exit of uninduced cultures. The wild-type strain recovered from stress after 4.5 hours, the isogenic Δ*mazEF* and Δ5 strains recovered after 5.6 hours and more than 9 hours, respectively (*N* = 3 replicates, except two replicates for induced Δ5 cultures). (**D**) We analyzed the effect of *mazF* overexpression on constitutive fluorescence encoded on plasmid pZS*-mCherry using flow cytometry. The results obtained for uninduced cultures are depicted in black and blue, and induced cultures in gray and light blue for the wild-type and Δ*mazEF* strain, respectively. Two hours after arabinose induction, induced populations of the wild-type and Δ*mazEF* strains exhibited increased fluorescence levels. After 6 h, induced populations of the wild-type strain exhibited bimodal distribution of mCherry fluorescence. Here plotted is one replicate per genotype, originating from the same exponential culture. Light grey distributions in panels (**D**) and (**E**) depict measurements of the reporterless strains MG1655 and BW27784, respectively, harboring plasmid pBAD-*mazF*. (**E**) Flow cytometry analysis of strain TB212, a BW27784 derivative deficient in L-arabinose metabolism, with a chromosomally encoded constitutive *mCherry* reporter, harboring pZS*-612 with a constitutive *gfp* reporter gene devoid of ACA sites. Two hours after induction of *mazF* expression, populations exhibited increased levels of mCherry and GFP fluorescence. After 6 h, bimodal patterns of mCherry fluorescence were detected, which correlate with the bimodal patterns of GFP fluorescence. Here, one replicate is presented, for further results see Supplementary Figure S7.

In this study, we investigated how autoregulation of *mazEF* expression affects growth of *E. coli*, in particular entry into stress and the recovery process. First, we established that the antitoxin MazE rescues bacterial growth during ectopic *mazF* overexpression from plasmid pBAD-*mazF*. Growth of the wild-type strain MG1655 was reduced during approximately the first 2 h after induction of *mazF* expression with 0.1% L-arabinose (Ara) (Figure 1A, Supplementary Figure S1). After this initial inhibition, growth recovered which can be attributed to MazF-dependent de-repression of the *mazEF* operon due to conditional cooperativity as suggested in (19), and the onset of neutralization of MazF by newly produced antitoxin MazE (5). This behavior was absent in an isogenic Δ*mazEF* strain and a Δ5 strain devoid of *mazEF, relBEF, chpB, yefM-yoeB* and *dinJ-yafQ* (40), which all belong to the RNA-degrading type II TA systems. Furthermore, both mutant strains exhibited a stronger decline in colony-forming units (CFU) after 6 h of *mazF* overexpression when compared to the wild-type strain (Figure 1B). Thus, growth modulation during prolonged *mazF* overexpression occurs in two phases: an initial transient growth cessation caused by large amount of free toxin, followed by antitoxin-dependent growth rescue.

Next, we specifically tested the dynamics of recovery from stress caused by *mazF* overexpression, and whether the recovery is mediated by the presence of the native antitoxin MazE (Δ*mazEF* strain) and other type II TA systems (Δ5 strain) (Figure 1C). We followed the recovery of the respective *E. coli* strains after 6 h of *mazF* overexpression, and defined the exit period as the time required for bacterial populations to reach maximum growth rate. The wild-type strain MG1655 recovered significantly faster in comparison to the Δ*mazEF* strain (4.5 h versus 5.6 h on average). Recovery of the Δ5 strain was drastically delayed, as the strain required >9 h to resume growth after release of the toxin stress (Supplementary Table S3). Thus, the antitoxin MazE mediates the exit from stress caused by *mazF* overexpression and the transition to normal growth by neutralization of MazF activity through direct interaction (5). Furthermore, our results suggest that the presence of other type II TA loci is likewise important for recovery from stress caused by *mazF* overexpression, which is in line with previous studies describing cross-talk between different TA systems (41,42), although the exact nature of this cross-talk still remains elusive. Together, the differences in the recovery times between the wild-type and deletion strains are a result of short-term effects (direct antitoxin neutralization), long-term growth arrest (viable but non-culturable cells), and possibly, cell death.

### Prolonged *mazF* overexpression fosters a high degree of cell-to-cell growth heterogeneity

We next used flow cytometry to test whether *mazF* overexpression leads to cell-to-cell growth rate heterogeneity, as our population measurements of stress and stress recovery might mask the presence of different growth rates or states at the single-cell level. To this end, we monitored the fluorescence of a constitutively expressed *mCherry* driven by the right phage λ promoter P_R,_ as a proxy for growth rate. Analysis of the expression of a constitutively expressed (i.e. unregulated) gene can be used to distinguish growth-rate dependence from the effects of gene regulation, as indicated in (25,26,43). In the absence of the phage λ repressor cI, P_R_ is not specifically regulated (44), and the level of mCherry fluorescence will depend on the constitutive production of the mCherry fluorescent protein and its degradation rate. Given that mCherry is only subjected to dilution but not degradation by proteases, differences in mCherry fluorescence reflect changes in growth rate (26,43). Similar fluorescent reporter systems have been previously used to assess growth states of cells within a population by flow cytometry (45). In our experiments, slow growth or cessation of growth will lead to accumulation of stable mCherry and an increase in fluorescence, while fast growth will lead to dilution of mCherry and a decrease in fluorescence. During *mazF* overexpression, mCherry is less diluted through growth due to growth rate reduction. However, the production rate of mCherry is most likely impeded as MazF besides mRNAs also degrades rRNA precursors (46,47), and generates non-stop translation complexes (48), thereby affecting overall translation. Nonetheless, several recent studies have shown that MazF-induced cells maintain a certain degree of transcriptional and translational capability (49,50). Therefore, during MazF overproduction, the level of mCherry fluorescence will increase when growth rate is reduced more strongly than protein production rate. In contrast, constitutive reporter fluorescence will decrease when a translational inhibitor is employed to restrict growth (Supplementary Figure S2), as previously shown in (43).

Flow cytometry analysis performed 2 h after *mazF* overexpression revealed that mCherry fluorescence increases unimodally, concomitant with a reduction in population growth (Figure 1D), without formation of subpopulations of different fluorescent intensities. Interestingly, 6 h after *mazF* overexpression, we observed high cell-to-cell variation in mCherry fluorescence. Bacterial cultures were characterized by bimodal distributions in mCherry fluorescence, which is suggestive of subpopulations of cells growing with different growth rates. We did not observe bimodality in the Δ*mazEF* (Figure 1D) and Δ5 strains (Supplementary Figure S3), which suggests that induction of the native *mazEF* operon and neutralization of MazF by the MazE antitoxin led to growth heterogeneity within a population. We note that the Δ5 strain exhibited increased variation in mCherry fluorescence even in the absence of *mazF* overexpression (Supplementary Figure S3), pointing towards an inherently high degree of growth rate variability.

To test whether the observed bimodality in mCherry fluorescence was based on variability in arabinose induction, we repeated the above experiments using derivatives of strain BW27784 that constitutively transports L-arabinose but is devoid of L-arabinose metabolism (32) (Figure 1E). Because arabinose is metabolized by the MG1655 derivatives, less arabinose remains to ectopically induce *mazF* expression after 6 hours of induction. We thus detected a higher left mode of the mCherry fluorescence distribution in the MG1655 strain (Figure 1D), on average 89% of the population, indicating a larger fraction of fast-growing cells. The BW27784-derived strain exhibited a higher right mode of the mCherry fluorescence distribution (Figure 1E), on average 89% of the population, indicating a larger fraction of non- or slow-growing cells.

In general, cultures induced with Ara concentrations ranging from 0.0001 to 0.36% showed bimodal mCherry fluorescence distributions after 5.5–6.5 h of *mazF* overexpression from pBAD-*mazF* (Supplementary Figures S4 and S5). We excluded that the observed variation in fluorescence arose from genetic variability through mutations in the plasmid-based expression system as determined by sequencing (Supplementary Information – Sequence S1). Moreover, multimodal fluorescence distributions were detected when cultures were induced repeatedly with L-arabinose (Supplementary Figure S4), or when different fluorescent reporter genes or inducible expression systems were employed (Supplementary Figure S5). Simultaneous analysis of fluorescence encoded by a chromosomally integrated *mCherry* reporter gene (the *mCherry* gene contains ACA sequence motifs, and is cleaved by MazF, Supplementary Figure S6) and a plasmid-based *gfp* reporter gene cured of ACA sites showed strong correlation in reporter fluorescence (Figure 1E, Supplementary Figure S7), indicating that the observed fluorescence distributions are not a direct consequence of *mCherry* mRNA cleavage by MazF. We also confirmed the results from a previous study (Supplementary Figure S8), which indicated heterogeneity in growth resumption after removal of the inducer of the *mazF* expression (41).

We furthermore observed that the extent of variation in mCherry fluorescence was dependent on the induction level of *mazF* expression; low l-arabinose concentrations (1 × 10^−7^%) caused neither growth arrest nor multimodal patterns of mCherry fluorescence (Supplementary Figure S4). Similarly, induction with 0.1% Ara from a single-copy chromosomally encoded P_BAD_-*mazF* system (Supplementary Figure S9) resulted in growth adaptation, a decrease in growth yield by 20% (similarly shown in (29)), and unimodal distributions of mCherry fluorescence (Supplementary Figure S10).

### MazF-dependent cleavage of the *mazF* mRNA alleviates the extent of cell-to-cell heterogeneity during arabinose induction

**Figure 2.**
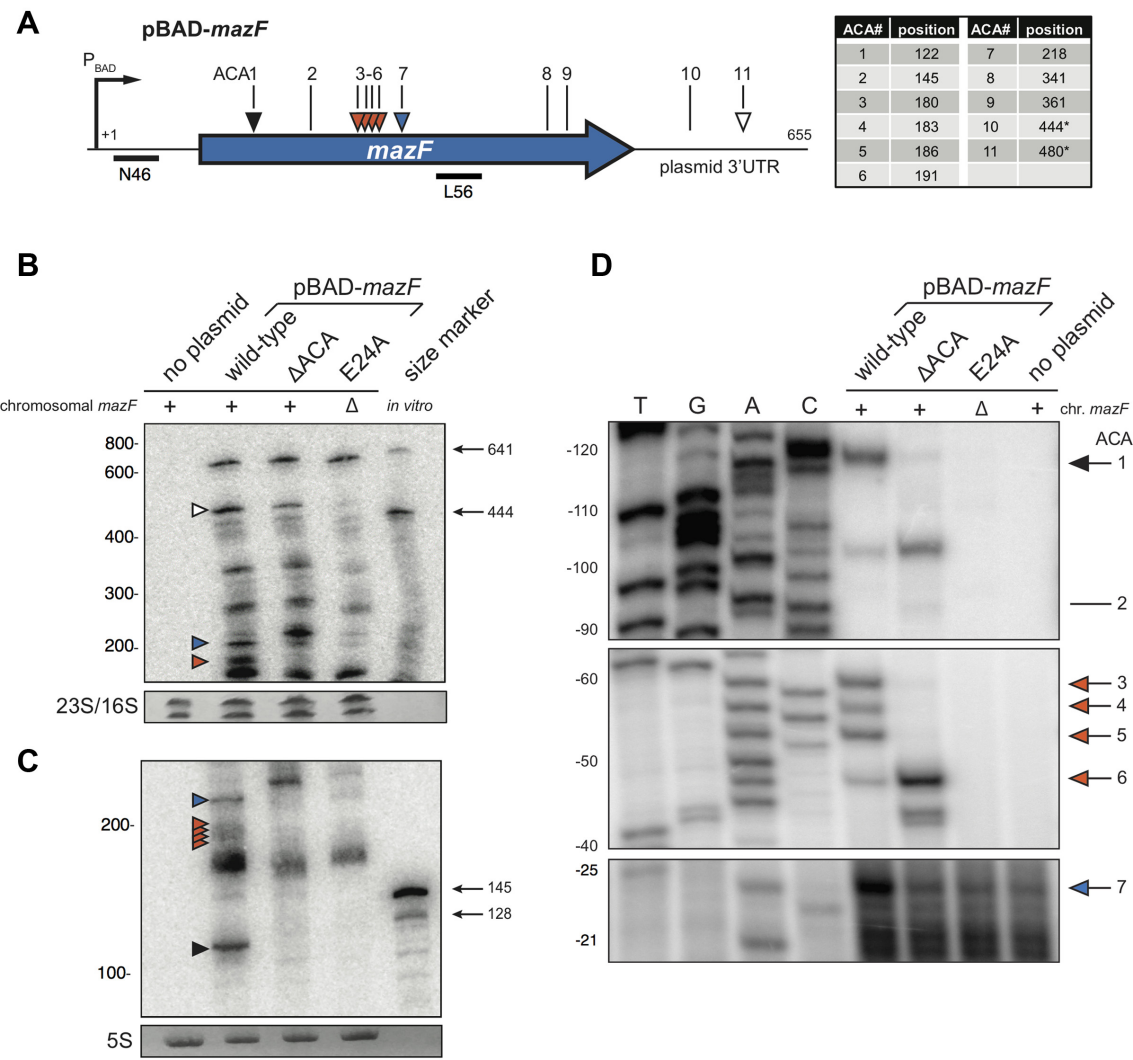
Cleavage of the *mazF* transcript *in vivo*. (**A**) A schematic showing the *mazF* gene. The promoter position and the ACA sites are indicated and their corresponding positions relative to the transcriptional start site are listed in the table on the right. The MazF-dependent cleavage sites experimentally verified in panels (**B**–**D**) are marked with arrows. ACA sites that are not part of the coding region, i.e. plasmid 3′ untranslated region (UTR), are marked with asterisks in the table. The annealing positions of probes N46 used for Northern blot analysis shown in (**B**) and (**C**), and L56 used for primer extension shown in (**D**) are indicated by bars. (**B**–**D**) Northern blot and primer extension analysis of total RNA extracted 30 min after induction with 0.2% Ara. Total RNA was separated on (**B**) 5% and (**C**) 10% denaturing polyacrylamide gel prior to blotting and hybridization with probe N46. Sizes depicted to the left side of the blots correspond to an RNA ladder. Methylene Blue staining of 23S, 16S and 5S rRNA served as loading control. Lanes depict from left to right: BW27784 without plasmid, MG1655 pBAD-*mazF* (plasmid-encoded wild-type *mazF*), MG1655 pBAD-*mazF*ΔACA (*mazF* coding sequence devoid of ACA sites), MG1655Δ*mazF* pBAD-*mazF*_E24A_ (*mazF*_E24A_ encoding an inactive MazF_E24A_), and the last lane are *in vitro* transcribed size markers. The white arrow depicts MazF-mediated cleavage within the 3′UTR transcribed from the pBAD plasmid backbone. Blue, orange and black arrows highlight MazF-mediated cleavage of the *mazF* transcript absent in the *mazF*ΔACA and *mazF*_E24A_ transcripts. (**D**) Cleavage sites at ACA sequences were mapped by primer extension using primer L56.

In line with a previous study suggesting that MazF degrades its own mRNA that contains multiple ACA sites *in vitro* (4), we hypothesized that *mazEF* mRNA abundance is subjected to an additional level of autoregulation through MazF-dependent cleavage *in vivo*. To investigate this hypothesis, we designed a *mazF* gene that is devoid of ACA sites (*mazF*ΔACA), while preserving the amino acid sequence of the wild-type MazF toxin. To test whether MazF cleaves its own transcript *in vivo*, we performed northern blotting on total RNA extracted 30 min after arabinose induction to determine cleavage patterns of *mazF, mazF*ΔACA and *mazF*_E24A_ transcripts (Figure 2A, Supplementary Figures S11 and S12). The *mazF*_E24A_ gene encodes an enzymatically inactive protein MazF_E24A_, thus the cleavage pattern of the *mazF*_E24A_ transcript indicates intrinsic *mazF* mRNA stability independent of *mazF* expression. Our analysis showed that *mazF*ΔACA and *mazF*_E24A_ transcripts yielded different cleavage patterns than the wild-type *mazF* transcript (Figure 2B), since in particular low-molecular weight fragments of MazF-specific degradation products were absent (Figure 2C). To pinpoint the cleavage positions with nucleotide resolution, we performed primer extension on the same samples, and confirmed that the observed pattern was due to MazF-dependent cleavage at ACA sites (Figure 2D). Taken together, this analysis highlights the MazF-dependent degradation of the *mazF* mRNA *in vivo*.

**Figure 3.**
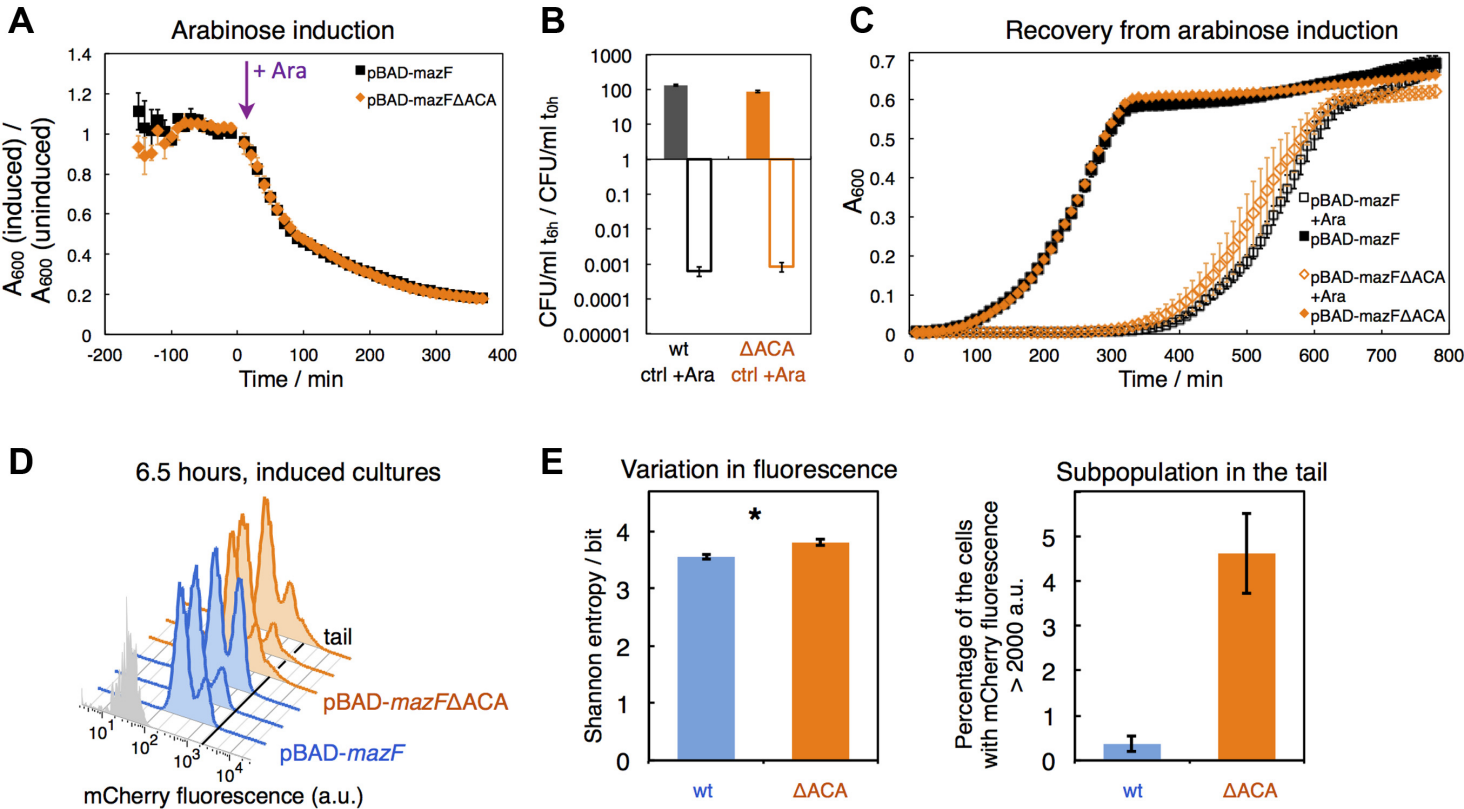
Population growth and constitutive mCherry fluorescence during *mazF* and *mazF*ΔACA overexpression. (**A**) We recorded growth of strain TB212 harboring either plasmid pBAD-*mazF*, or plasmid pBAD-*mazF*ΔACA. The experimental setup is identical as in Figure 1A. Population growth was similarly impaired during *mazF* and *mazF*ΔACA overexpression. (**B**) Changes in CFU were determined after 6 hours in induced (empty bars) and control (filled bars) cultures compared to the exponential cultures, and there were no differences in CFU counts between *mazF* (empty black bar) and *mazF*ΔACA overexpression (empty orange bar). (**C**) Six hours after *mazF* or *mazF*ΔACA overexpression all cultures were washed and re-suspended in fresh media. The experimental setup is identical as in Figure 1C. No significant differences in population growth were detected upon recovery from arabinose induction, regardless of *mazF* or *mazF*ΔACA overexpression (*N* = 3 biological replicates). (**D**) Bimodal distributions of mCherry fluorescence were measured 6 h after arabinose induction. Measurements of induced TB212 cultures harboring plasmid pBAD-*mazF* are shown in blue, and harboring plasmid pBAD-*mazF*ΔACA in orange. Light grey distributions depict measurements of the reporterless strain MG1655 pBAD-*mazF*. (**E**) The variation in mCherry fluorescence, computed as Shannon entropy, was significantly higher after 6.5 hours of *mazF*ΔACA overexpression (average entropy = 3.80 bits) than *mazF* overexpression (average entropy = 3.56 bits) supported by *t*-test (*P* = 0.04, *N* = 3 replicates). (The difference in the variation in mCherry fluorescence is significant as well when comparing squared coefficients of variations; average SCV = 0.0090 for *mazF*ΔACA overexpression, average SCV = 0.0076 for *mazF* overexpression; *P* = 0.018.) We also quantified the percentage of cells that display mCherry fluorescence above the threshold of 2000 a.u. indicated with a black line in panel (**D**), which corresponds to the percentage of the cells in tails.

Since it has been previously hypothesized that stabilizing the *mazF* transcript by curing it from ACA sequence motifs leads to higher toxin levels (30,49), we investigated whether *mazF* mRNA cleavage by MazF affects population growth. Bacterial growth was measured during and after excessive overexpression from either plasmid pBAD-*mazF* or from plasmid pBAD-*mazF*ΔACA. However, we did not observe differences in population growth (Figure 3A), colony formation after arabinose induction (Figure 3B), and length of the recovery phase (Figure 3C). Nevertheless, using flow cytometry to test for changes in population heterogeneity, we found that the absence of MazF-dependent cleavage of the *mazF* transcript amplified cell-to-cell heterogeneity in mCherry fluorescence during arabinose induction (Figure 3D). We observed more cells with higher fluorescent values 6.5 h after *mazF*ΔACA overexpression than after *mazF* overexpression, manifested as longer right tails in mCherry fluorescence distributions (Figure 3E, Supplementary Table S4), which we interpreted as a larger fraction of slow-growing cells. Again, the variation in fluorescence was not attributed to mutations in the plasmid-based expression systems P_BAD_-*mazF* and P_BAD_-*mazF*ΔACA (Supplementary Information—Sequence S1, Sequence S2). Thus, MazF-mediated degradation of the *mazF* transcript is a potential autoregulatory feature controlling MazF levels, which may affect the extent of growth rate heterogeneity between single cells in clonal bacterial populations during *mazF* expression.

### Increasing growth rate heterogeneity underlies increasing variation in mCherry fluorescence during *mazF* overexpression in a microfluidic device

**Figure 4.**
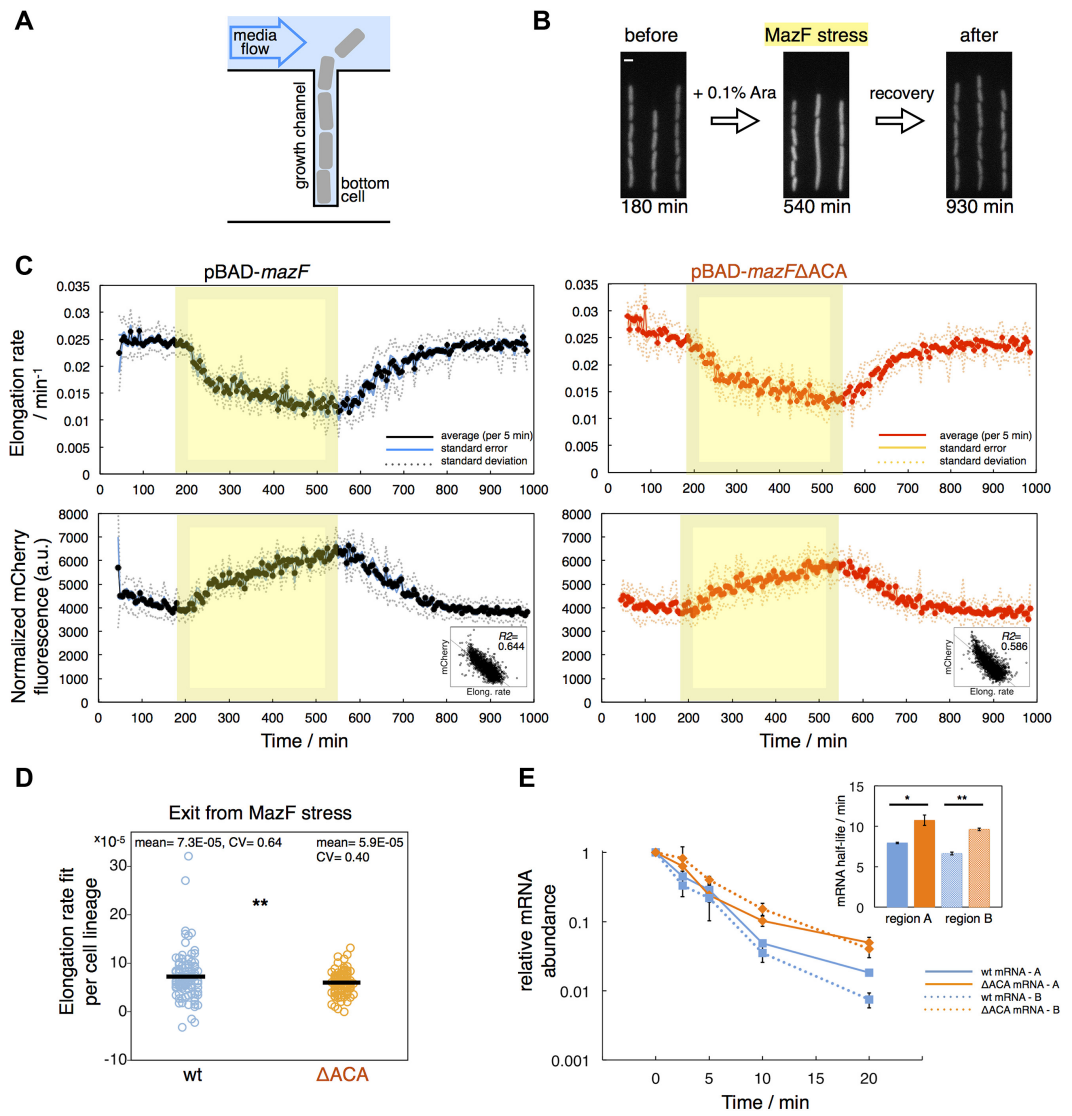
Cell elongation rate and mCherry fluorescence during arabinose induction in a microfluidic device, and recovery from stress. (**A**) A schematic depiction of the microfluidic device used (38). We analyzed 110 bottom cells of strain TB212 that harbor pBAD-*mazF*, and 98 bottom cells that harbor pBAD-*mazF*ΔACA. (**B**) Strain TB212 was grown for 3 hours to allow for steady-state growth (time 0–180 min). *mazF* or *mazF*ΔACA overexpression was induced by adding 0.1% Ara for a period of 6 hours (time 185–540 min). After switching to media without arabinose, the cells were monitored during the recovery phase (time 545–990 min). White scale bar indicates length of 2 μm. (**C**) During arabinose induction (yellow window), the cell elongation rate decreased and λP_R_-*mCherry* reporter fluorescence increased due to the slow down of growth and mCherry accumulation. Average values over a 5 minute-window are presented in black and red circles, for *mazF* and *mazF*ΔACA overexpression, respectively. Inserts: Differences in the mCherry fluorescence reflect changes in the cell elongation rate; Spearman's rho = –0.761, *P*-value = 0.00, *R*^2^ = 0.644 for the experiment with the strain harboring pBAD-*mazF* (*N* = 2709 cells) and rho = –0.730, *P*-value = 0.00, *R*^2^= 0.586 for pBAD-*mazF*ΔACA (*N* = 2546 cells). (**D**) Recovery from stress is dependent on MazF-mediated cleavage of the *mazF* mRNA. A line was fitted through the cell elongation rate values per each cell lineage, during *t* = 545–725 min to infer exit from stress. Single cells recovered from stress caused by *mazF* overexpression 23% faster than from stress caused by *mazF*ΔACA overexpression (Mann-Whitney test, *P* = 0.014; Kolmogorov–Smirnov test, *P* = 0.006). (**E**) Stability of the A (ACA-free) and B (contains one ACA site in the wild-type transcript) regions of the *mazF* and *mazF*ΔACA transcripts was determined by quantitative reverse transcription PCR over five time points. mRNA abundance was quantified relative to the *cysG* transcript, and normalized to the zero time point (see Supplementary Methods); error bars present standard deviation. Insert: The half-life of *mazF* mRNA was on average 1.4 times shorter than the half-life of *mazF*ΔACA mRNA, supported by *t*-tests (*P* = 0.047 for the A region, *P* = 0.0002 for the B region, *N* = 3 biological replicates); error bars present standard error of the mean.

To describe the differences in constitutive mCherry fluorescence arising from *mazF* and *mazF*ΔACA overexpression in detail, we employed time-lapse microscopy in microfluidic devices (38) to directly observe single-cell growth (measured as cell elongation rate), mCherry fluorescence, and cell length (Figure 4, Supplementary Figure S13). In these microfluidic devices cells are captured in blunt-end growth-channels that are open to a feed channel where newborn cells are removed by media flow (Figure 4A and B). Arabinose induction resulted in increased formation of filamentous cells and large variation in cell length in general (Figure 4B, middle panel), which has been previously reported to occur during overexpression of other type II TA systems (27). To quantitatively compare results from flow cytometry and microfluidic experiments, we analyzed all cells from static images obtained after 3 and 6 h of arabinose induction, and found that the cell-to-cell variation in mCherry fluorescence was higher during *mazF*ΔACA overexpression compared to *mazF* overexpression, similar to the flow cytometry analysis (Supplementary Figure S14).

We further analyzed time-lapse data of bottom cells in each microfluidic channel (Figure 4A) before, during and after induction from either pBAD-*mazF* (Supplementary Movie S1) or from pBAD-*mazF*ΔACA (Supplementary Movie S2). mCherry fluorescence and cell elongation rate were significantly correlated throughout the experiment (Figure 4C, Supplementary Figure S13B), corroborating that differences in single-cell growth rate underlie differences in mCherry fluorescence. As indicated by our results from population-level measurements (Figures 1A and 3A), single-cell growth was reduced but not arrested during arabinose induction, and reduction occurred in at least two steps (Figure 4C, upper panels). Initially, the cell elongation rate decreased rapidly, followed by a slower decrease towards the end of the arabinose induction period, resembling an adaptation phase. Moreover, towards the end of the *mazF* overexpression period, the variation in cell elongation rate increased (Supplementary Figure S13C), implying that the bacterial population consisted of cells growing with different rates.

We also tested whether there are differences in how single cells recover from *mazF* and *mazF*ΔACA overexpression after removal of the inducer. Although we did not observe differences in population growth between *mazF* and *mazF*ΔACA overexpression (Figure 3A and C), our single-cell analysis showed that there are subtle differences during the exit from stress (Figure 4D). The cells recovered 23% faster after induction from pBAD-*mazF* than after induction from *mazF*ΔACA. Quantitative reverse transcription PCR showed decreased stability of a *mazF* mRNA region containing one ACA site (dashed lines in Figure 4E). Moreover, cleavage at ACA sites reduced *mazF* mRNA half-life in general (full lines in Figure 4E). Together, these results indicate that MazF-dependent *mazF* transcript cleavage mediates faster removal of functional *mazF* mRNAs from the cells during recovery from stress.

### Cell length measured before division fluctuates during *mazF* overexpression

**Figure 5.**
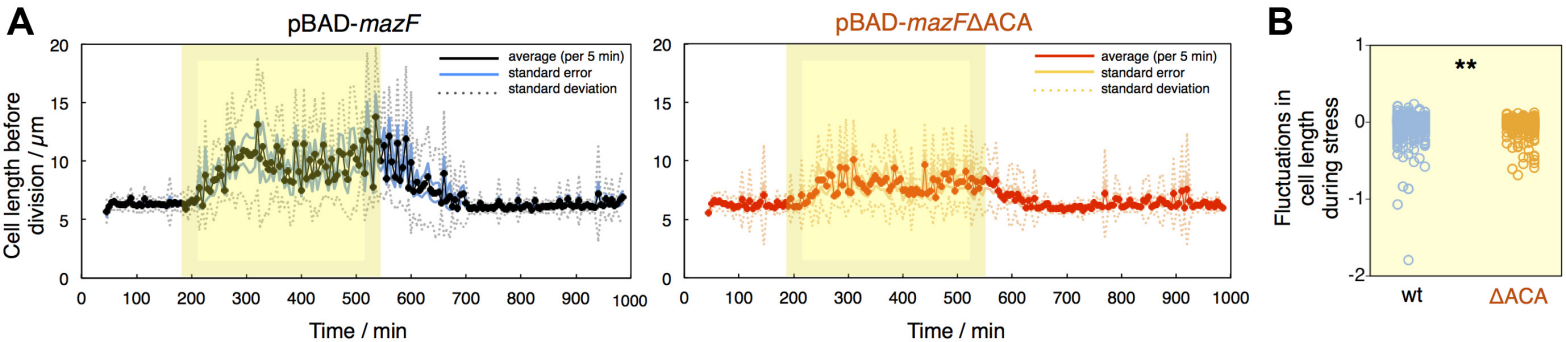
Fluctuations in cell length in response to *mazF* and *mazF*ΔACA overexpression. (**A**) Cell length measured before division fluctuates during *mazF* overexpression (yellow window; the same experiment as in Figure 4). To evaluate whether there are long-term effects of *mazF* expression, we applied Sign test for related samples for cells just before arabinose induction (one cell division prior the stress i.e. 180th min of the experiment) and during recovery from stress (cell division between 800th-830th min). We found no statistical significance, meaning that the cells recovered completely; *P* = 0.294 (0.614) for induction from pBAD-*mazF* (pBAD-*mazF*ΔACA). (**B**) We calculated slopes for the plots of the cell length measured before division vs. time between two consecutive division events in a cell lineage during arabinose induction, and analyzed the differences in fluctuations during *mazF* (blue circles) and *mazF*ΔACA (orange circles) overexpression. When the slope value is negative, the parameter's value decreased in the following division, and *vice versa*. The results indicate that the fluctuations in cell length are more drastic during induction from pBAD-*mazF*; we measured a significant difference between the distributions of measured fluctuations, but not in their mean levels, during *mazF* and *mazF*ΔACA overexpression (** stands for *P*< 0.01 Kolmogorov–Smirnov test, *P* = 0.0014; Mann–Whitney test, *P* = 0.301).

By using microfluidic-based time-lapse microscopy we were able to quantify not only phenotypic heterogeneity across cells in a clonal population at a given time point, but also temporal variability, i.e. variation in phenotypic traits of the individual cells in time. We next analyzed differences in length of cells harboring pBAD-*mazF* or pBAD-*mazF*ΔACA, and found that cell length measured just before division fluctuated during arabinose induction (Figure 5A). However, the extent of fluctuations was larger during *mazF* overexpression compared to *mazF*ΔACA overexpression (Figure 5B), suggesting that the fluctuations were a direct consequence of MazF-dependent *mazF* mRNA degradation. Upon *mazF* expression, cellular mRNAs including *mazF* mRNA are cleaved, which leads to decreased MazF production and temporary recovery, which is further promoted by the MazE antitoxin neutralizing MazF. Since *mazF* is constantly ectopically expressed, MazF levels increase, which again affects cellular processes. This temporal variability in length of individual cells might further imply that proteins involved in coordination of cell elongation and division, and impairment of cell wall synthesis, are encoded by mRNAs that are prone to MazF-dependent degradation and sensitive to MazF levels (46,51,52). During recovery from stress, there were no phenotypic differences between cells harboring pBAD-*mazF* or pBAD-*mazF*ΔACA (Supplementary Figure S15). Furthermore, comparing cell length in individual cell lineages before arabinose induction and during recovery from stress (Figure 5A) we found no differences, indicating that there are no long-term effects of the stress caused by *mazF* and *mazF*ΔACA overexpression on single-cell physiology.

### Autoregulation through *mazEF* mRNA cleavage triggers fluctuations in the MazF level

**Figure 6.**
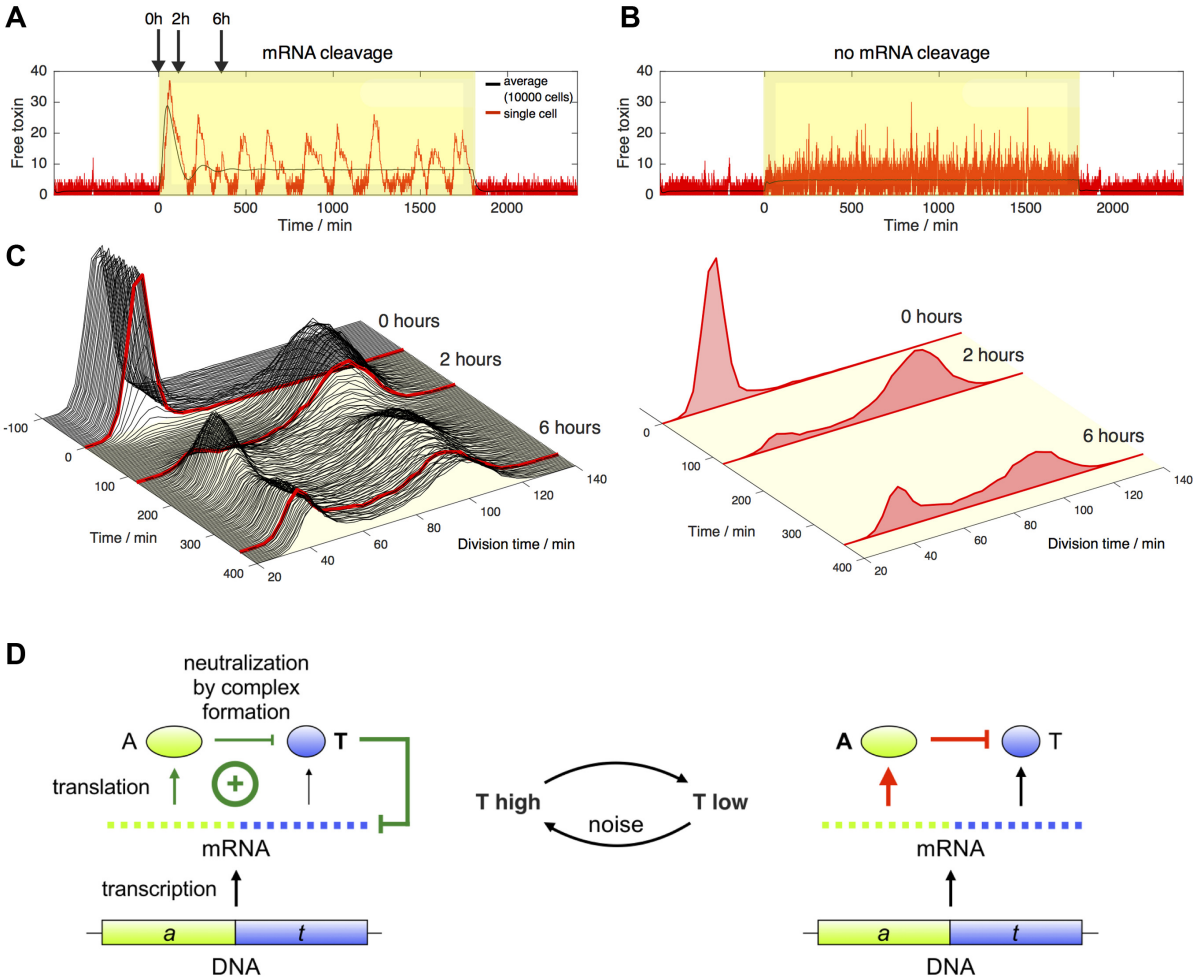
Numerical simulations of the regulation of the *mazEF* toxin–antitoxin module expression and its influence on cell division times. (**A** and **B**) During a period of stress (yellow window), the degradation of the MazE antitoxin was increased by a factor 3 in the model. The graphs in red show the free toxin level in a single cell (**A**) including or (**B**) excluding cleavage of the *mazEF* mRNA by the MazF toxin. The averages over 10 000 cells are indicated in black. The results suggests that MazF-dependent *mazEF* mRNA cleavage amplified fluctuations in the MazF level in single cells during stress (red line), and increased initial spike in the MazF level upon entry to stress for the entire simulated population of cells (average black line). (**C**) Left: Three-dimensional plot showing the distribution of the division times before and after the onset of stress. The simulation was run for 10 000 cells carrying the *mazEF* toxin–antitoxin module including mRNA cleavage by the MazF toxin. Right: Histograms showing the distribution of the division times at the onset of stress, and after 2 and 6 h of stress. The histograms correspond to the lines shown in red in the left panel. (**D**) Schematic representation of the post-transcriptional regulation of the *mazEF* operon. Both the antitoxin A and the toxin T are translated from a polycistronic mRNA. The antitoxin can inhibit the activity of the toxin by binding to it. Noise can induce a switch from low to high toxin levels. In this case, the toxin can additionally cleave the mRNA and as such prevent the production of both toxin and antitoxin. These interactions form a positive feedback loop, as indicated in green on the left hand side.

In order to understand the influence of the *mazEF* mRNA cleavage on *mazF* expression, and the resulting fluctuations in cell length, we simulated *mazEF* expression dynamics in individual cells, based on a previously established model (18) (Supplementary Table S5). Consistent with our finding that MazF cleavage reduces the half-life of its own mRNA (Figure 4E), we extended the model so that it included MazF-dependent *mazEF* mRNA cleavage as an autoregulatory feature (Supplementary Figure S16), which was also implemented in a recent computational model of cellular resource allocation (52). We compared the MazF levels between two settings, which included or excluded *mazEF* mRNA cleavage, over a time course that comprised exposure to stress. This episode of stress was either simulated by an increase in the toxin production rate (which corresponds to the experimental approach used in this study, Supplementary Figure S17), or by an increase in the antitoxin degradation rate (which would be a realistic effect *in vivo*, given that cellular proteases are upregulated during nutritional stress (6,8), Figure 6).

We continued to explore the dynamics of *mazEF* expression in stressful conditions defined through enhanced degradation of the MazE antitoxin. The behavior for the simulated *mazEF* module including and excluding *mazEF* mRNA cleavage is very similar in the absence of stress, manifesting as a very low, close to zero level of the free MazF toxin. However, during an episode of stress (yellow shaded regions in Figure 6), we observed increased fluctuations in the free MazF level in the simulations where the *mazEF* mRNA cleavage is included in the model (red lines in Figure 6A compared to B). These simulations were based on cleavage of the entire *mazEF* mRNA (see Supplementary Figure S18 for the full simulation); nonetheless, we also performed simulations with ‘differential’ mRNA cleavage, as the *mazE* coding sequence has two ACA sites and the *mazF* coding sequence has nine ACA sites (30), thus the *mazF*-encoding part of the mRNA is 4.5 times more likely to be cleaved (Supplementary Figure S19). Similarly to the cleavage of the entire *mazEF* mRNA (Figure 6A, Supplementary Figure S18), differential mRNA cleavage resulted in increased fluctuations in the MazF level, however, with higher average MazE levels and lower average MazF levels (Supplementary Figure S19).

Overall, the fluctuations in MazF levels arising from *mazEF* mRNA cleavage potentially reflect experimentally measured fluctuations in cell length (Figure 5). These large fluctuations were absent in the simulations when the *mazEF* mRNA was not degraded by MazF, indicating that MazF-dependent *mazEF* mRNA cleavage is indeed an autoregulatory feature controlling the *mazEF* expression dynamics. Moreover, in an initial response to stress, all cells respond quasi-synchronously by an increase in the toxin levels, leading to a peak in the average toxin level (black lines in Figure 6A compared to B). This leads to all cells increasing their division time in that initial phase (Figure 6C, distribution of division times after 2 h of stress), which corresponds to the behavior observed in Figure 1D. After a few hours, the cells still have similar spikes in the toxin levels, but these no longer occur synchronously. Therefore, there are now two types of cells in the simulated population analyzed after 6 hours of stress (Figure 6C): fast-growing and slow-growing cells, leading to the bimodality seen in Figure 1D.

While the regulation at the transcriptional level is a negative regulation, where the binding of toxin–antitoxin complexes to the operator inhibits the production of mRNA at moderate toxin to antitoxin ratios, the post-transcriptional regulation contains a positive feedback loop, as previously included in the model for post-transcriptional regulation of the *relBE* operon (16). The addition of a positive feedback loop in the autoregulation of *mazEF* operon by including mRNA cleavage by MazF in our model can explain increased fluctuations in the free toxin levels observed in Figure 6A. A high enough stochastic increase in the free toxin level can lead to an increased degradation of mRNA, which in turn causes a decrease in the production of toxin and antitoxin. Since the toxin is more stable than the antitoxin, this will reduce the level of antitoxin in the cell, and finally ensure a further increase in the free toxin level (Figure 6D). We further studied the robustness of this behavior by exploring how the fluctuating free toxin levels change with the model parameters (Supplementary Figure S20). Omitting negative regulation at the transcriptional level does not qualitatively change the system dynamics (Supplementary Figure S20E), however it gives rise to higher free toxin levels (Supplementary Figure S20F-H). These results suggest that the two-level regulation of *mazEF* expression generates controlled bursts in the amount of free MazF toxin in single cells during stress.

## DISCUSSION

In this study we investigated how autoregulation of *mazEF* expression at the transcriptional and post-transcriptional level affects growth of *E. coli* populations and single cells during ectopic *mazF* expression, as well as during recovery after stress caused by *mazF* expression. Our data show that *mazF* overexpression promotes cell-to-cell growth heterogeneity in isogenic populations of *E. coli*. Bacterial growth is transiently restricted upon *mazF* overexpression, however during extended stress the MazE antitoxin is required for adaptation to *mazF* expression, which leads to growth rate heterogeneity, and the onset of recovery of growth in general.

In particular, we experimentally demonstrated the impact of post-transcriptional autoregulation of *mazF* expression on the phenotypic heterogeneity across cells in a clonal population, as well as on the variability in phenotypic traits of the individual cells in time. First, MazF-dependent cleavage of the *mazF* transcript might serve as a mechanism to alleviate the extent of cell-to-cell growth rate heterogeneity elicited by MazF activation, as measured by decreased variation in constitutive reporter fluorescence (Figure 3D). Second, *mazF* transcript cleavage generates temporal variability in length of individual cells during ectopic *mazF* overexpression as indicated by microfluidic-based time-lapse microscopy (Figure 5). This finding was further corroborated with the model that indicates an increased variability in *mazEF* expression dynamics in the presence of MazF-dependent mRNA cleavage, resulting in the stochastic pulsed excitation of MazF levels in single cells (Supplementary Figure S17). The model suggests that such fluctuations in the MazF level can also occur during nutritional stress or other adverse conditions when proteolysis of MazE is the main process that promotes MazF activation (6–8).

Moreover, the results of numerical simulations indicate that the initial spike in the MazF concentration in response to stress conditions is higher when the system is autoregulated through *mazEF* mRNA cleavage (black lines in Figure 6A and B). This could generally be advantageous when bacteria encounter adverse conditions, as mounting a strong stress response program is favorable to rapidly adapt to the hostile environment. Eventually, when the stressor is no longer present in the environment, escaping timely from the stress response program and resuming growth might be favorable for regrowth of the surviving population, and as such represents an efficient stress exit strategy. Overall, our study supports that both autoregulatory features, MazE-dependent repression (Figure 1C) and MazF-dependent *mazF* mRNA cleavage (Figure 4D), facilitate exit from the stress caused by *mazF* overexpression.

In general, control of gene expression through direct transcriptional repression has been suggested to reduce phenotypic heterogeneity (53), and to positively affect the response time to environmental perturbations (54). Besides this negative feedback through repression, positive feedback has also been identified within bacterial regulatory networks. Positive feedback loops can function as effective switching mechanisms, by delaying activation of the regulatory network and thus adjusting cellular response to stimuli (55). Native autoregulation of the *mazEF* operon contains both types of feedback: negative regulation through direct antitoxin neutralization, negative regulation at the transcriptional level, and a positive feedback loop at the post-transcriptional level.

MazF is not the only sequence-specific type II toxin encoded on the *E. coli* genome. The ribosome-independent toxins MqsR and ChpB cleave at GCU and ACD sites (D is G, A or U, but not C), respectively, while the ribosome-dependent toxin YafQ cleaves AAA codons (9,30). *mqsRA, chpSB*, and *yafQ-dnaJ* transcripts contain sequence-specific cleavage sites for cognate toxins. Specifically, *mqsRA* and *chpSB* transcripts have less toxin-specific cleavage sites in the toxin coding sequence than in the antitoxin coding sequence. On the other hand, the toxin *yafQ* mRNA part harbors seven AAA codons, whereas the antitoxin *dnaJ* mRNA part harbors three AAA codons. Thus, cleavage of toxin–antitoxin mRNA by the cognate toxin might generate different dynamics of toxin expression among type II TA systems in *E. coli* in general.

Lastly, it has been previously argued that ectopic *mazF* overexpression in *E. coli* may invoke artificial physiological responses (9) and, eventually, cell death (6,7,11,36). In our microfluidic setup, arabinose induction of *mazF* expression from a medium-copy plasmid (7) resulted in growth rate reduction at the single-cell level rather than in complete growth arrest. We did however observe death of a minority of cells, representing 4.3% of the total bottom cell count (4 cells lysed and 1 cell stopped dividing during or after *mazF* overexpression). During *mazF*ΔACA overexpression there was neither lysis nor growth arrest of bottom cells. Therefore, cell death was not a predominant phenotype during steady state growth conditions and constant *mazF* overexpression.

To conclude, bacterial populations might employ the MazF-mediated stress response to rapidly adjust and optimize their growth under fluctuating environmental conditions. It is conceivable that the extent of MazF activation upon physiological stress resembles mild ectopic *mazF* expression in laboratory setups (19,56). Even though stressed populations activate MazF at low levels (29,57), the MazF-mediated pathway might be an important strategy for cells to respond to stress rapidly and efficiently, and to optimize growth resumption after the stressor is gone. When bacterial cells encounter long-term adverse conditions in environment or in host, the subtle MazF-mediated response might be complemented by the activation of other TA systems (26,41,42) or stress response mechanisms (58,59) that allow for complex regulation of gene expression and growth, and eventually result in persistence.

## AVAILABILITY

Raw flow cytometry files were deposited to the FlowRepository (http://flowrepository.org) with assigned Repository IDs: FR-FCM-ZYG3 (data for Figure 1D), FR-FCM-ZYG4 (Figure 1E), and FR-FCM-ZYG5 (Figure 3D). All microscopy data are available via doi.org/10.15479/AT:ISTA:74.

## Supplementary Material

Supplementary Data
