# Supplementary material for: Autoregulation of mazEF expression underlies growth heterogeneity in bacterial populations
Source: Nucleic Acids Res. 2018 Feb 8;46(6):2918–31. doi: 10.1093/nar/gky079 (PMC5888573; doi:10.1093/nar/gky079)
Supplement: Supplementary Data [file gky079_supp.zip › Nikolic_Supplementary Information.pdf]

Supplementary Information

Autoregulation of *mazEF* expression underlies growth heterogeneity in bacterial populations

by
Nela Nikolic, Tobias Bergmiller, Alexandra Vandervelde, Tanino G. Albanese, Lendert Gelens, and Isabella Moll

Table of contents

Supplementary Figures
Figures S1-S20

Supplementary Tables
Tables S1-S5

Supplementary Movies
Movies S1 and S2

Sequence Alignments
Sequences S1 and S2

Supplementary Methods

Model Description

Supplementary References

Supplementary Figures

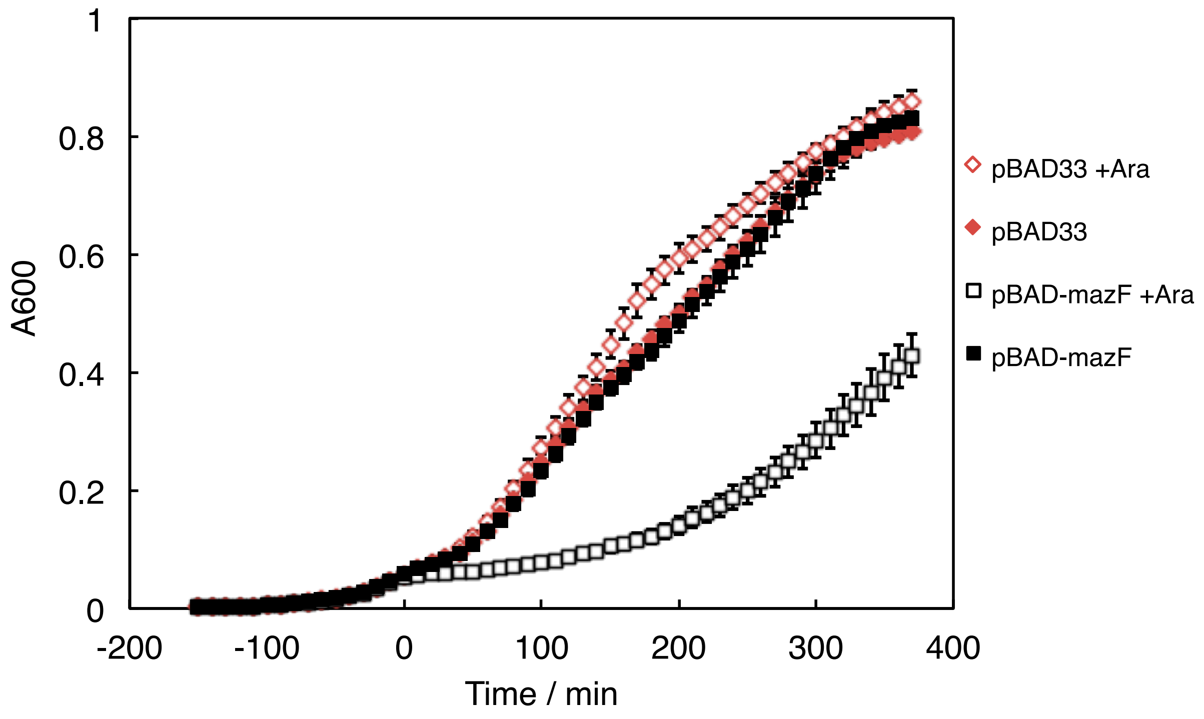

Figure S1. Determining leakiness of the P_{BAD} promoter in MG1655 pBAD-*mazF*.

We monitored growth of strain MG1655 harboring either plasmid pBAD-*mazF* (black squares) or empty vector pBAD33 (dark red diamonds). At t= 0 min, 0.1% Ara was added to half of the exponentially growing cultures to induce *mazF* overexpression (empty markers); the other cultures served as a control (filled markers). Growth was measured in the plate-reader as absorbance at 600 nm (A_{600}) in N= 4 biological replicates. Error bars present standard error of the mean. Comparing the uninduced cultures of the strain harboring either of the two plasmids (filled squares and diamonds), there were no significant differences in their growth during non-stressful conditions. Therefore, possible leaky expression from the P_{BAD} promoter in MG1655 derivatives during non-stressful conditions is negligible.

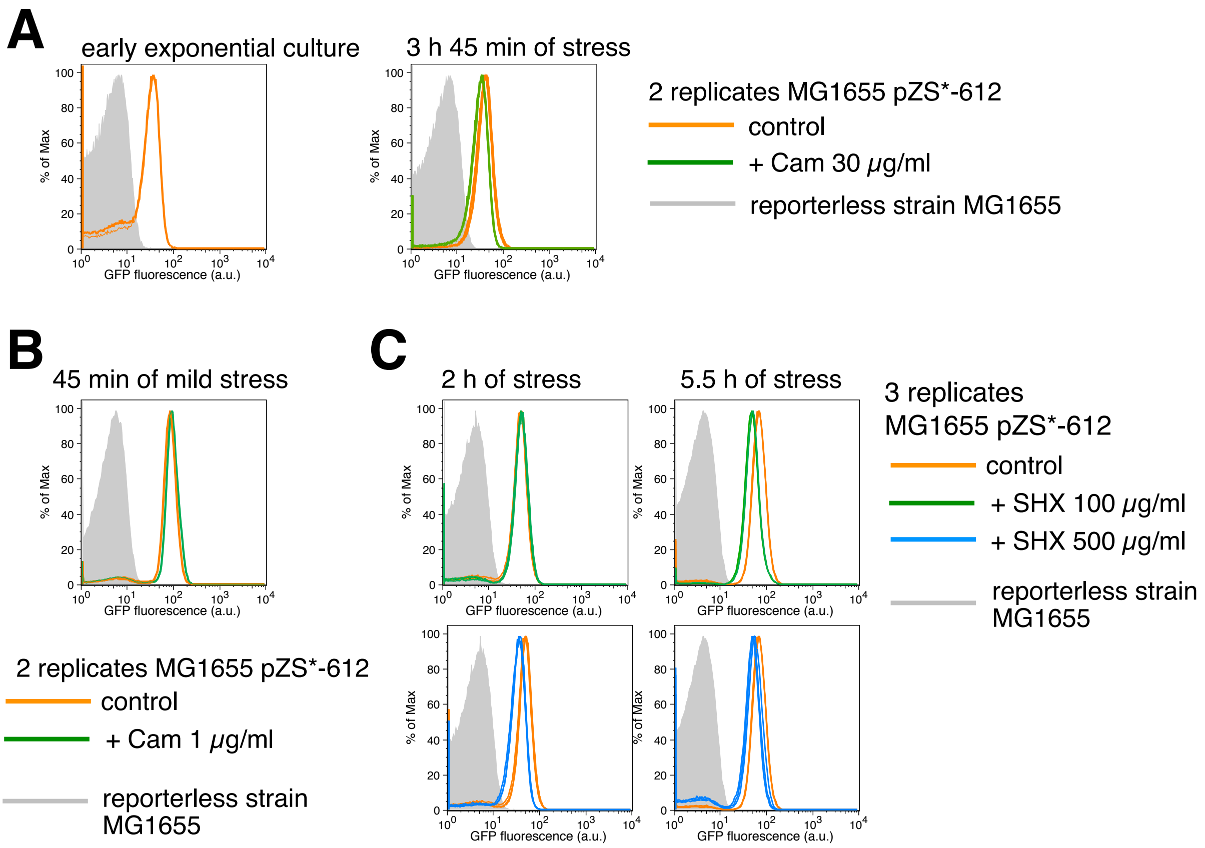

Figure S2. Fluorescence of the constitutive GFP reporter during amino acid starvation and antibiotic treatment.

We investigated how antibiotic treatment and amino acid starvation influence expression of the constitutive *gfp* reporter gene devoid of ACA sites over the course of three experiments. The antibiotic chloramphenicol (Cam) is an inhibitor of protein synthesis. Serine hydroxamate (SHX) is a serine analog that inhibits seryl-tRNA synthetase thereby inducing the ppGpp-dependent stress response and an overall decrease in translation. When growth is modulated by use of translation inhibitors, the fluorescence encoded by constitutively expressed reporter genes decreased with reducing growth rate, as indicated in [Scott et al. 2010]. In comparison, upon *mazF* overexpression we observed increased fluorescence with reducing growth rate (e.g., Figure 1D, Figure 1E, Figure S4B). **(A)** Cam-treatment decreases level of the GFP fluorescence in a homogeneous fashion, **(B)** and mild treatment with Cam does not have a significant impact on the GFP fluorescence. **(C)** Treatment with SHX led to similarly decreased *gfp* expression levels without the formation of distinct subpopulations with different fluorescent intensities.

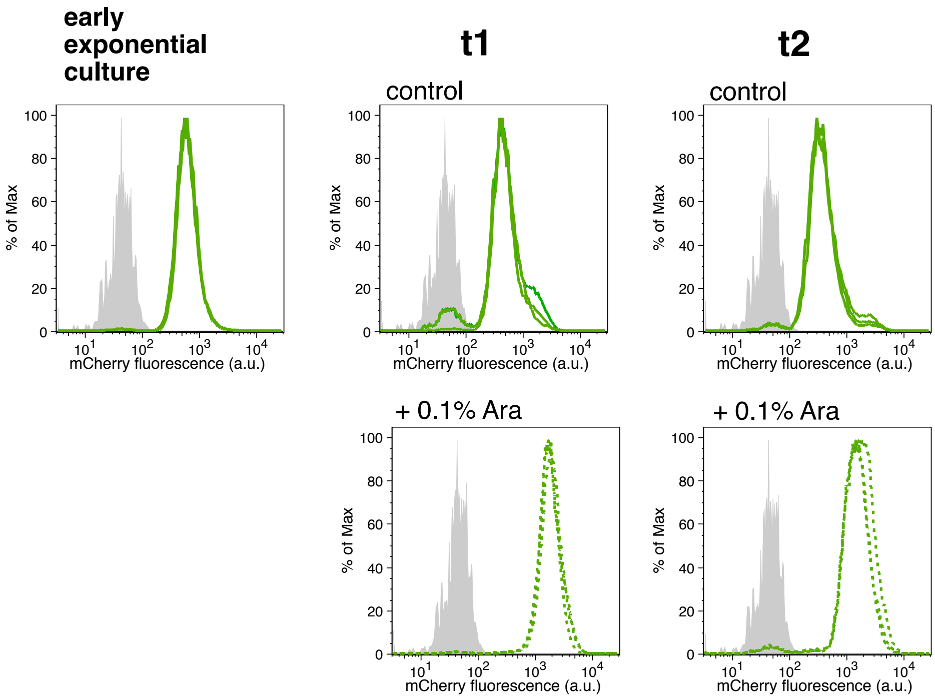

Figure S3. Influence of 5 TA systems on constitutive *mCherry* expression during *mazF* overexpression. We analyzed the effect of *mazF* overexpression on constitutive mCherry fluorescence in the Δ5 strain, which is MG1655 Δ*mazEF* Δ*chpB* Δ*relBEF* Δ*yefM-yoeB* Δ*dinJ-yafQ* (LVM100 [Tsilibaris et al. 2007]). Flow cytometry measurements performed 2 hours (time point t1) and 6 hours (time point t2) after arabinose induction of *mazF* expression showed an increased level of mCherry fluorescence. Interestingly, there was increased variation in the mCherry fluorescence in the absence of *mazF* overexpression, indicating elevated growth heterogeneity in the middle (t1) and late (t2) exponential phase (N= 3 biological replicates). Grey distributions in the flow cytometry plots depict reporterless strain MG1655 harboring plasmid pBAD-*mazF*, same measurements as shown in Figure 1D.

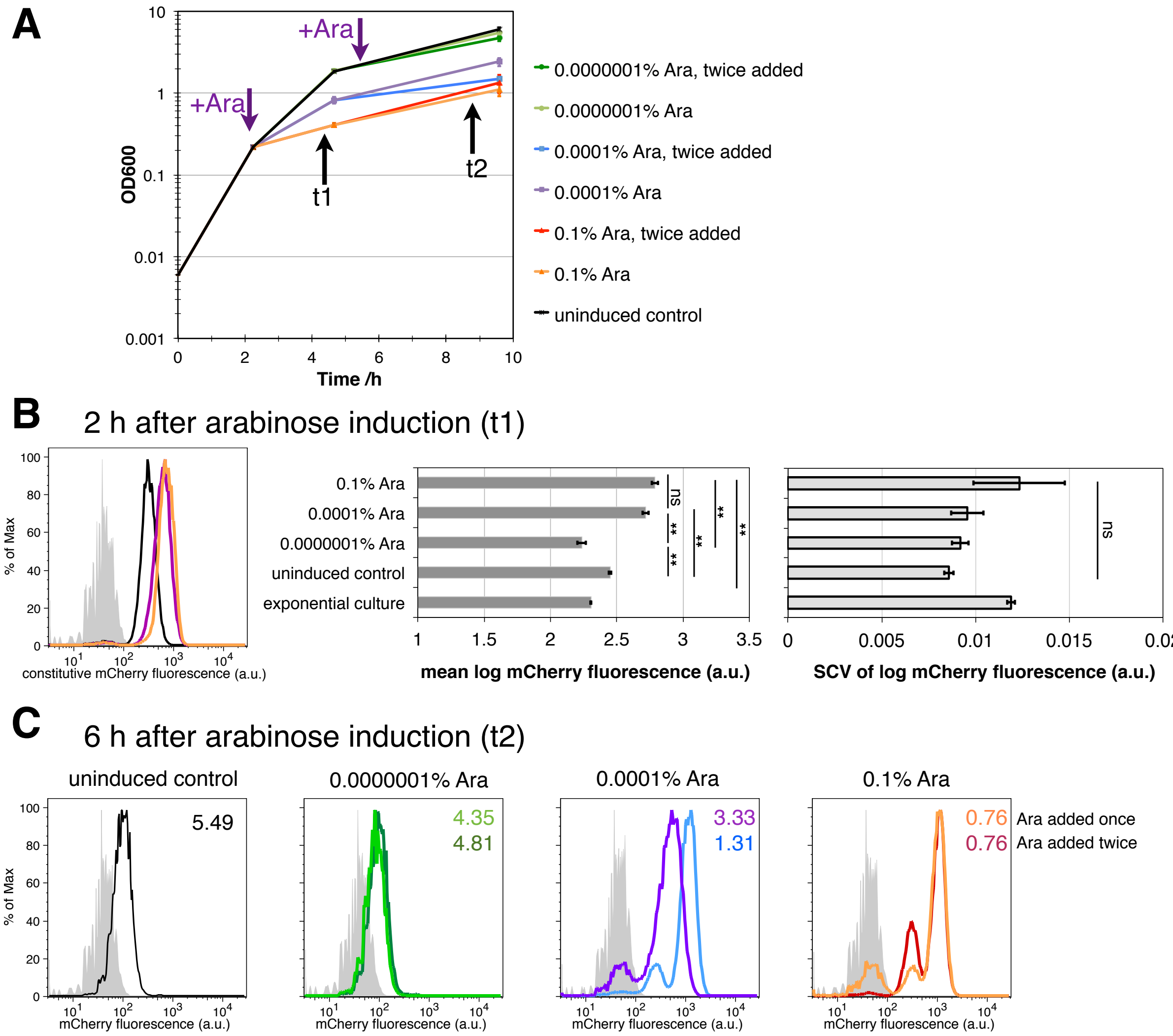

Figure S4. Effect of *mazF* overexpression on bacterial growth and constitutive gene expression, after repeated addition of arabinose.

(A) Exponentially growing cultures of TB212 strain harboring plasmid pBAD-*mazF* were equally divided into flasks, one serving as a control, and arabinose (Ara) was added to the other flasks in reported concentrations. Increased inducer concentrations led to stronger reductions in growth. Indicated cultures were induced a second time with the same arabinose concentration. Flow cytometry measurements were done 2 hours (time point t1) and 6 hours (time point t2) after arabinose induction for four replicates. **(B)** Flow cytometry analysis 2 hours after arabinose induction; measurements of the culture induced with 0.1% Ara is shown in orange, induced with 0.0001% Ara in purple, and uninduced culture is shown in black. Light grey distributions depict measurements of reporterless strain BW27784 pBAD-*mazF*. The *mCherry* expression increased in a homogeneous fashion: increased inducer concentrations led to increased mean *mCherry* expression levels. The increase in the mean level of mCherry fluorescence was significant for all used concentrations of the inducer except for the difference between 0.1% Ara vs. 0.0001% Ara induction (2-tailed, paired *t*-test; p= 0.0017 for uninduced vs. 0.1% Ara, p= 0.0007 for uninduced vs. 0.0001% Ara, p= 0.0048 for uninduced vs. $1*10^{-7}$% Ara, p= 0.2006 for 0.1% Ara vs. 0.0001% Ara, p= 0.0023 for 0.1% Ara vs. $1*10^{-7}$% Ara, p= 0.0001 for 0.0001% Ara vs. $1*10^{-7}$% Ara). Although the trend indicates the higher concentration of inducer added, the higher the variation in reporter expression, we found no statistically significant differences between inducer concentration and variation in reporter

expression (2-tailed, paired *t*-test; p= 0.280 for uninduced vs. 0.1% Ara, p= 0.400 for uninduced vs. 0.0001% Ara, 0.488 for uninduced vs. $1*10^{-7}$% Ara, p= 0.282 for 0.1% Ara vs. 0.0001% Ara, p= 0.336 for 0.1% Ara vs. $1*10^{-7}$% Ara, p= 0.771 for 0.0001% Ara vs. $1*10^{-7}$% Ara). Error bars present standard error of the mean; ns stands for non significant, ** stands for p< 0.01. **(C)** 6 hours after induction of *mazF* expression, the populations induced with 0.1% or 0.0001% of Ara exhibited multimodal patterns of mCherry fluorescence. The populations that were stressed by adding $1*10^{-7}$% of Ara did not exhibit heterogeneous mCherry fluorescence. Here depicted is one replicate. Corresponding optical density (OD_{600}) values are indicated for each flow cytometry measurements. The color code is the same as in panel **(A)**.

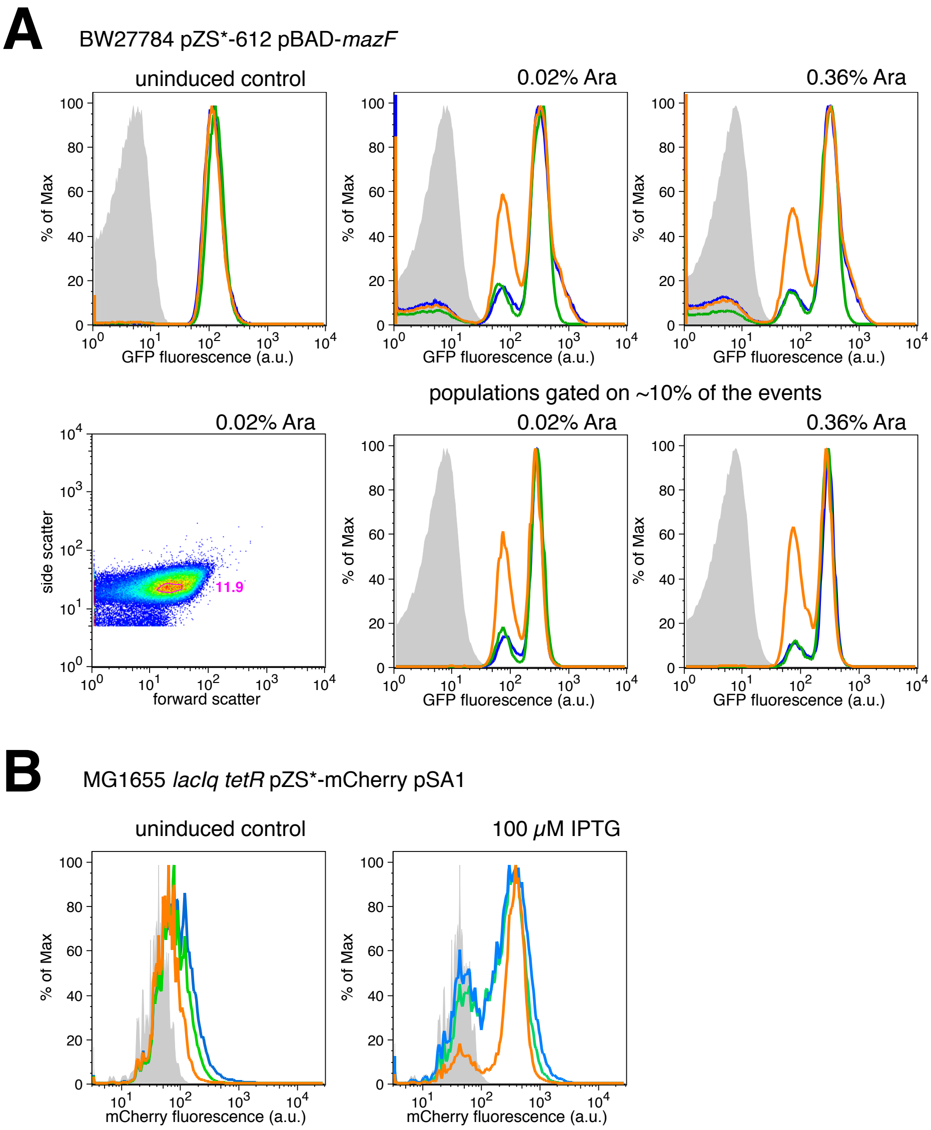

Figure S5. Reporter fluorescence after prolonged *mazF* overexpression.

Exponentially growing cultures were equally divided into flasks: one flask served as control, to the other flasks the inducer was added to ectopically express *mazF*. Three biological replicates per each condition are depicted in green, blue and orange. In each panel, the same replicates measured in different conditions are depicted in the same color. **(A)** The cultures were grown in minimal medium containing 0.18% glucose, without maltose. The samples were analyzed after 5.5 hours with flow cytometer Calibur, and in total 100,000 events were acquired per sample. Multimodal distribution of GFP fluorescence was measured in entire bacterial populations, as well as when the populations were gated on the size parameters, forward scatter and side scatter, and only 10,000-12,000 cells were plotted (here plotted a gate capturing 11.9% of the population). The grey distribution corresponds to the fluorescence of the negative control, BW27784 pBAD-*mazF*. **(B)** *mazF* was ectopically induced with 100 μM IPTG from plasmid pSA1 encoding IPTG-inducible *mazF* gene. The cultures were analyzed after 6 hours with flow cytometer Fortessa, and in total 50,000 events were acquired. We measured mCherry fluorescence from the plasmid-based λP_R-*mCherry* system in strain MG1655 *laclq tetR*. Grey distribution corresponds to the fluorescence of negative control, MG1655 pSA1.

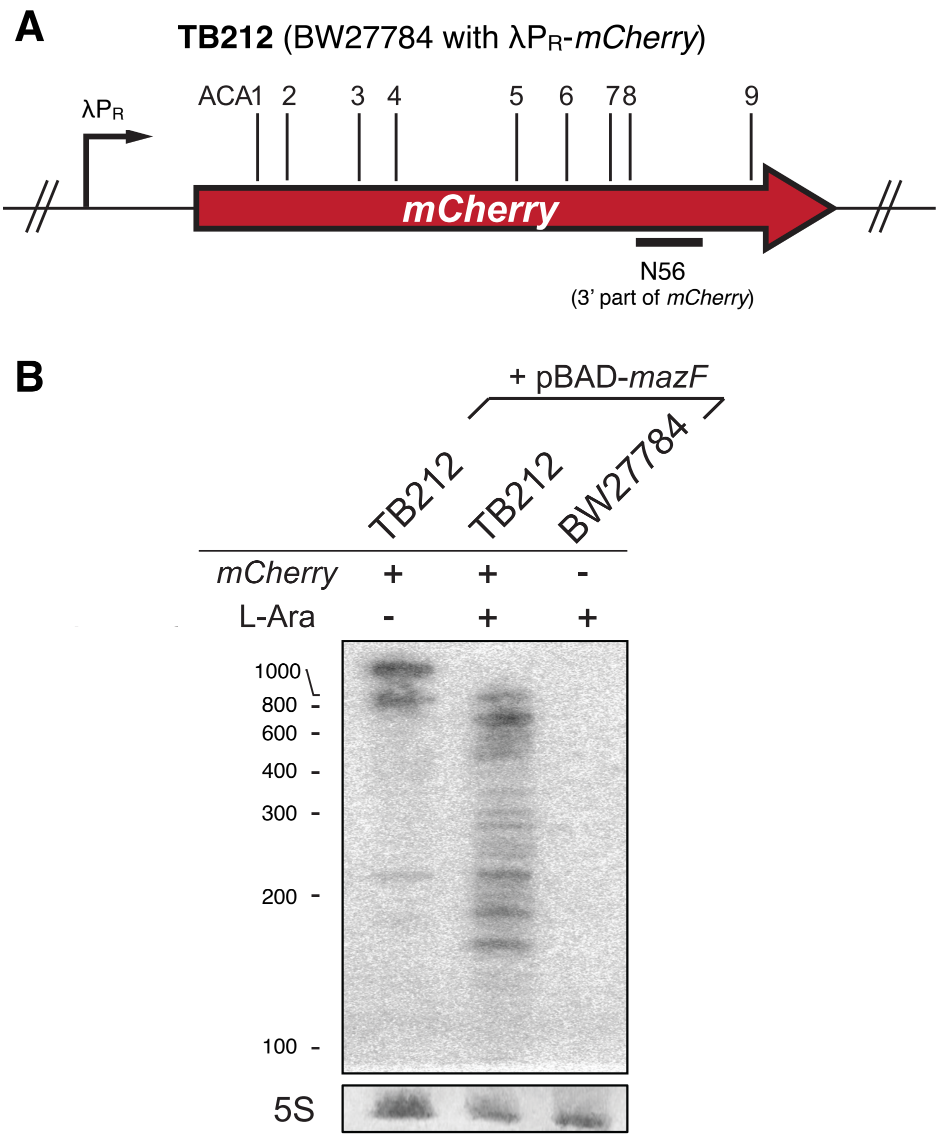

Figure S6. *mazF* overexpression results in *mCherry* transcript degradation.

(A) Schematics of the chromosomally integrated λP_R-*mCherry* locus in strain TB212 (a derivative of BW27784) containing nine ACA sites in the coding sequence. **(B)** Northern blot analysis of the *mCherry* mRNA during *mazF* overexpression. TB212 harboring pBAD-*mazF* was grown in LB medium with chloramphenicol. At an OD_{600} of 0.2, the culture was split in two flasks of which one was induced with 0.2% L-Ara for *mazF* overexpression, and the second was treated with water (uninduced control). Total RNA was extracted 1 hour after induction. BW27784 pBAD-*mazF* was used as a negative control. The samples were hybridized with probe N56.

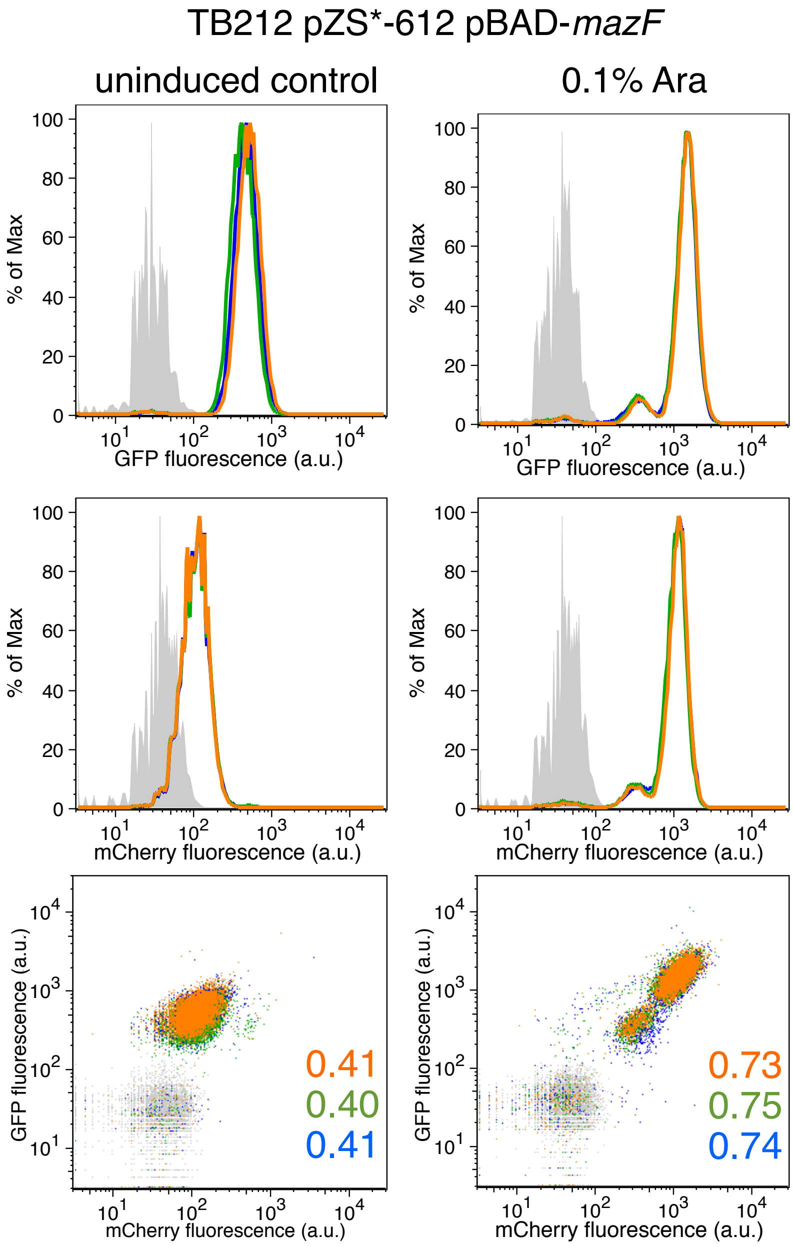

Figure S7. Simultaneous analysis of the chromosomally integrated mCherry reporter and the plasmid-based GFP reporter.

Strain TB212 (a derivative of BW27784 with chromosomally integrated λP_R-*mCherry*) harboring pBAD-*mazF* was additionally transformed with pZS*-612 [Sauert 2015, Oron-Gottesman et al. 2016] (one replicate identical as in Figure 1E). We analyzed in total N= 50,000 events per sample with flow cytometer Fortessa 6 hours after *mazF* expression induced with 0.1% Ara. We observed a strong correlation between the GFP and mCherry signals during *mazF* overexpression (numbers indicated in the flow cytometry plots: Spearman's rho range from 0.73-0.75, p-values= 0), in all three replicates depicted in blue, green and orange.

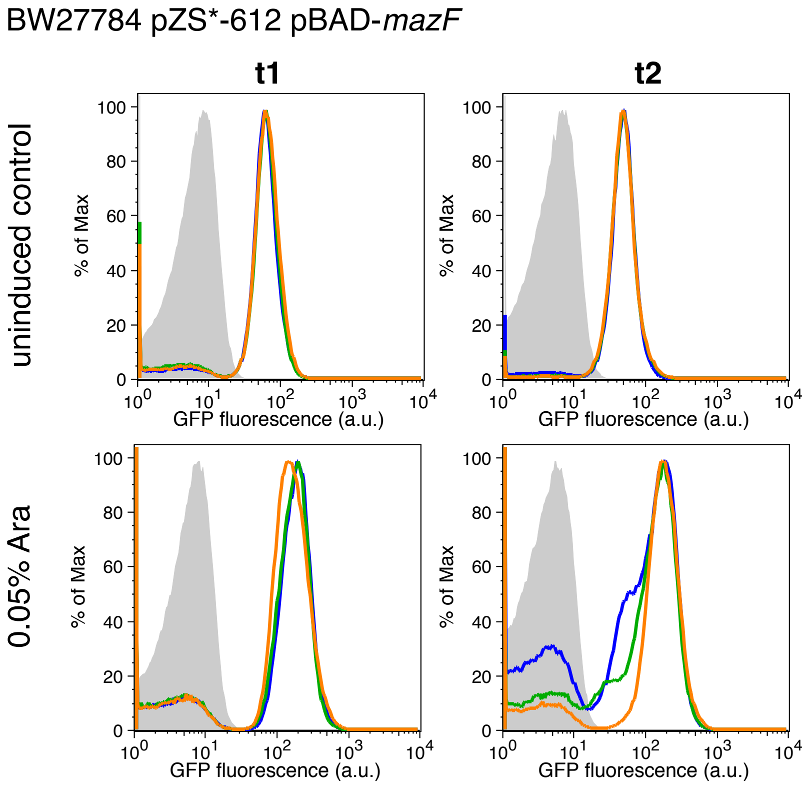

Figure S8. Heterogeneity in the *gfp* reporter gene expression after removal of the inducer. Exponentially growing cultures were equally divided into two culture tubes: one tube served as a control, and arabinose was added to the other tube as the inducer of *mazF* overexpression. The cultures were analyzed with Calibur 2 hours after *mazF* overexpression (time point t1). The induced cultures were then spun down, washed with 1x PBS and resuspended into fresh, prewarmed media. The bacterial populations were analyzed after 1 hour of recovery (time point t2). Three biological replicates (green, blue and orange distribution) were analyzed per each condition. Grey distribution corresponds to the fluorescence of reporterless strain BW27784. An increased variation in the GFP fluorescence was detected 1 hour after removal of arabinose.

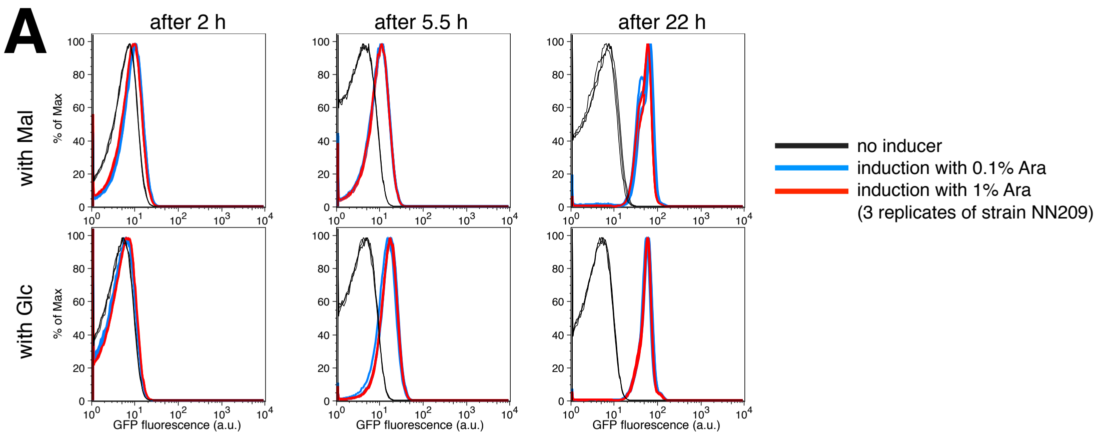

timepoint		media with Mal<br>percentage of the cells with GFP signal	population OD	media with Glc<br>percentage of the cells with GFP signal	population OD
2 h (t1)	1% Ara	14.5 ± 0.20	0.86	2.8 ± 0.06	1.31
	0.1% Ara	18.6 ± 0.06	0.79	2.1 ± 0.01	1.15
	control	4.4 ± 0.18	0.99	1.1 ± 0.03	1.46
5.5 h (t2)	1% Ara	24.7 ± 0.40	3.24	66.8 ± 0.55	3.30
	0.1% Ara	23.7 ± 0.32	3.36	63.8 ± 3.03	3.02
	control	0.5 ± 0.01	3.60	0.6 ± 0.04	3.56
22 h (t3)	1% Ara	97.7 ± 0.13	5.97	97.8 ± 0.21	4.75
	0.1% Ara	96.3 ± 0.54	6.00	97.7 ± 0.25	4.64
	control	4.8 ± 0.64	6.29	1.2 ± 0.04	5.00

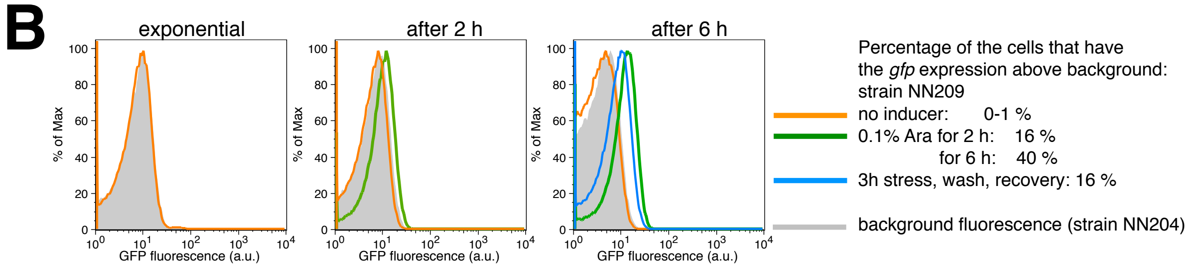

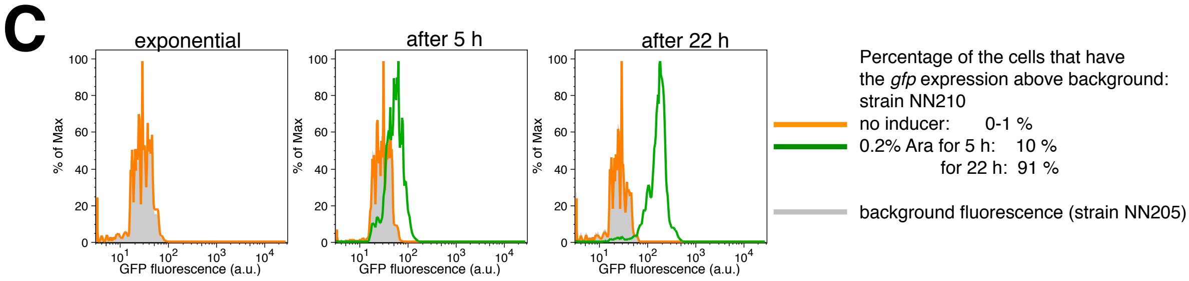

Figure S9. Testing inducibility of the chromosomally integrated P_{BAD} expression system by measuring expression of the P_{BAD}-*gfp* construct.

GFP fluorescence was analyzed with FACS Calibur in the experiments **(A)** and **(B)** or with Fortessa in the experiment **(C)**. Fractions of the cells with GFP signal were calculated for entire measured populations, without gating: in the experiment **(A)** as the number of cells above a general threshold ($\log_{10}$(threshold)= 1.12), for **(B)** and **(C)** as the number of cells above a threshold that is determined by the background fluorescence measured in the same condition.

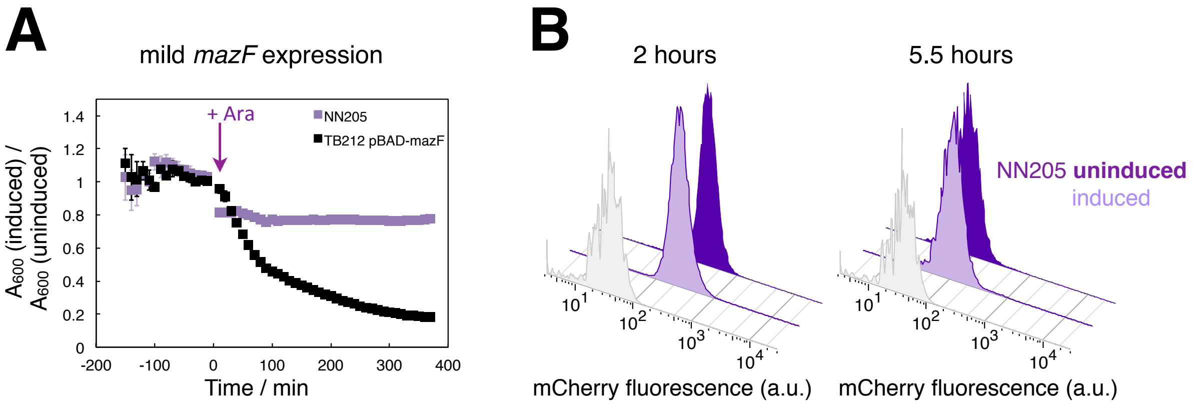

Figure S10. Excessive versus mild induction of *mazF* expression.

(A) We tested how the level of induction of *mazF* expression influences bacterial growth as well as constitutive mCherry fluorescence. For this purpose we recorded growth of strain NN205 (a BW27784 derivative), which has chromosomally integrated P_{BAD}-*mazF* system as well as constitutive *mCherry* reporter gene. We also recorded growth of strain TB212 carrying plasmid pBAD-*mazF* (same data as presented in Figure 3A). *mazF* expression was induced by adding 0.1% Ara after 2.5 hours of the exponential growth in a plate-reader. In comparison to the growth reduction when *mazF* was excessively expressed from plasmid pBAD-*mazF* (black line), mild arabinose induction led to growth adaptation and a decrease of the maximum growth yield by 20% (purple line). **(B)** We measured constitutive mCherry fluorescence of strain NN205, 2 hours and 5.5 hours after mild *mazF* expression, and observed no bimodal distribution of mCherry signal in induced cultures. Here plotted is one replicate, originating from the same exponential culture. Grey distributions in the flow cytometry plots depict reporterless strain BW27784 pBAD-*mazF*.

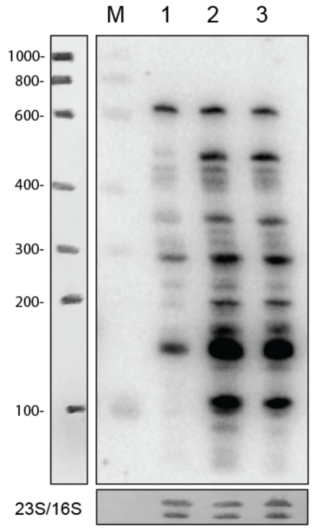

Figure S11. Similar degradation profiles of the *mazF* mRNA in MG1655 and BW27784 genetic backgrounds.

Ectopic *mazF* expression was induced with 0.2% Ara, and the probe used for Northern blot was N46 (see Table S2), specific for detection of the 5' untranslated region (UTR) of mRNA under synthetic P_{BAD} promoter in pBAD plasmids. *mazF*$_{E24A}$ overexpression [Tripathi et al. 2014] in the MG1655Δ*mazF* background [Müller et al. 2016] depicted in lane 1 serves as a control for MazF-independent *mazF* mRNA cleavage, as encoded MazF$_{E24A}$ is enzymatically inactive. Northern blot analysis of the *mazF* mRNA overexpressed from pBAD-*mazF* in the BW27784 genetic background [Khlebnikov et al. 2001] is depicted in lane 2, and in the MG1655 genetic background [Blattner et al. 1997] in lane 3. Samples in lanes 1 and 3 are the same RNA samples used for the blots in Figure 2. Methylene Blue staining of 23S and 16S rRNA serves as loading control, and lane M depicts size marker. RNA ladder is additionally represented as sizes on the left side of the blot. There were no differences in the *mazF* mRNA profile between the BW27784 and MG1655 strains.

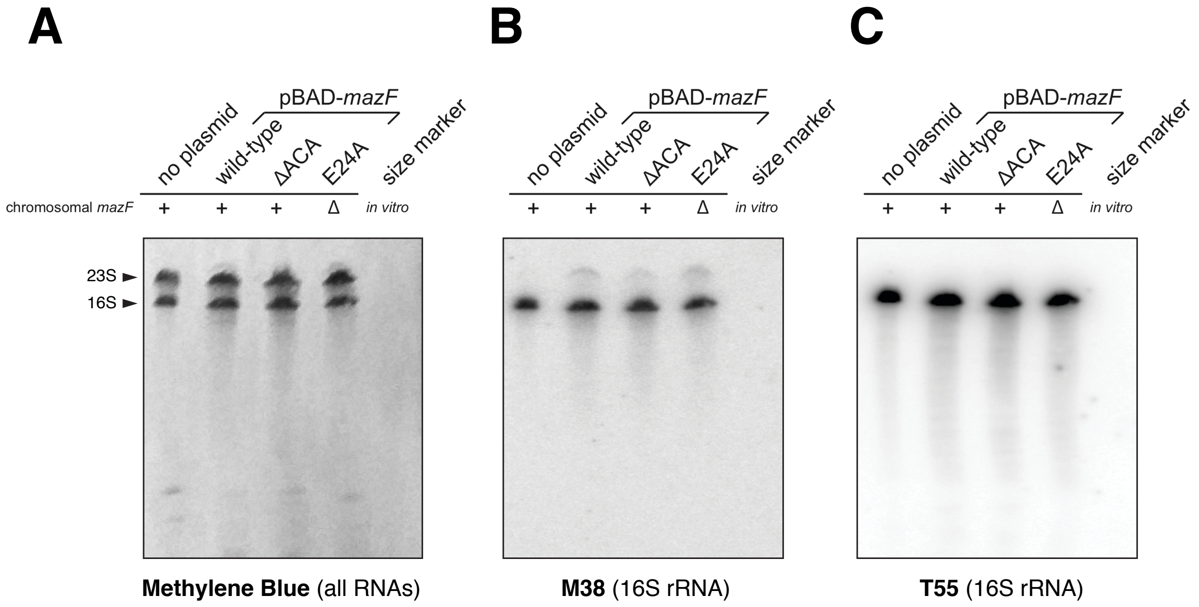

Figure S12. Effect of *mazF* overexpression on rRNA stability.
Shown is the full blot of the rRNA loading control presented in Figure 2B. rRNA was detected by **(A)** Methylene Blue staining, or by re-hybridization with probes **(B)** M38, and **(C)** T55 (probe 16S_P1 from [Mets et al. 2017]) for specific detection of 16S RNA. There were no specific rRNA degradation products during *mazF* overexpression.

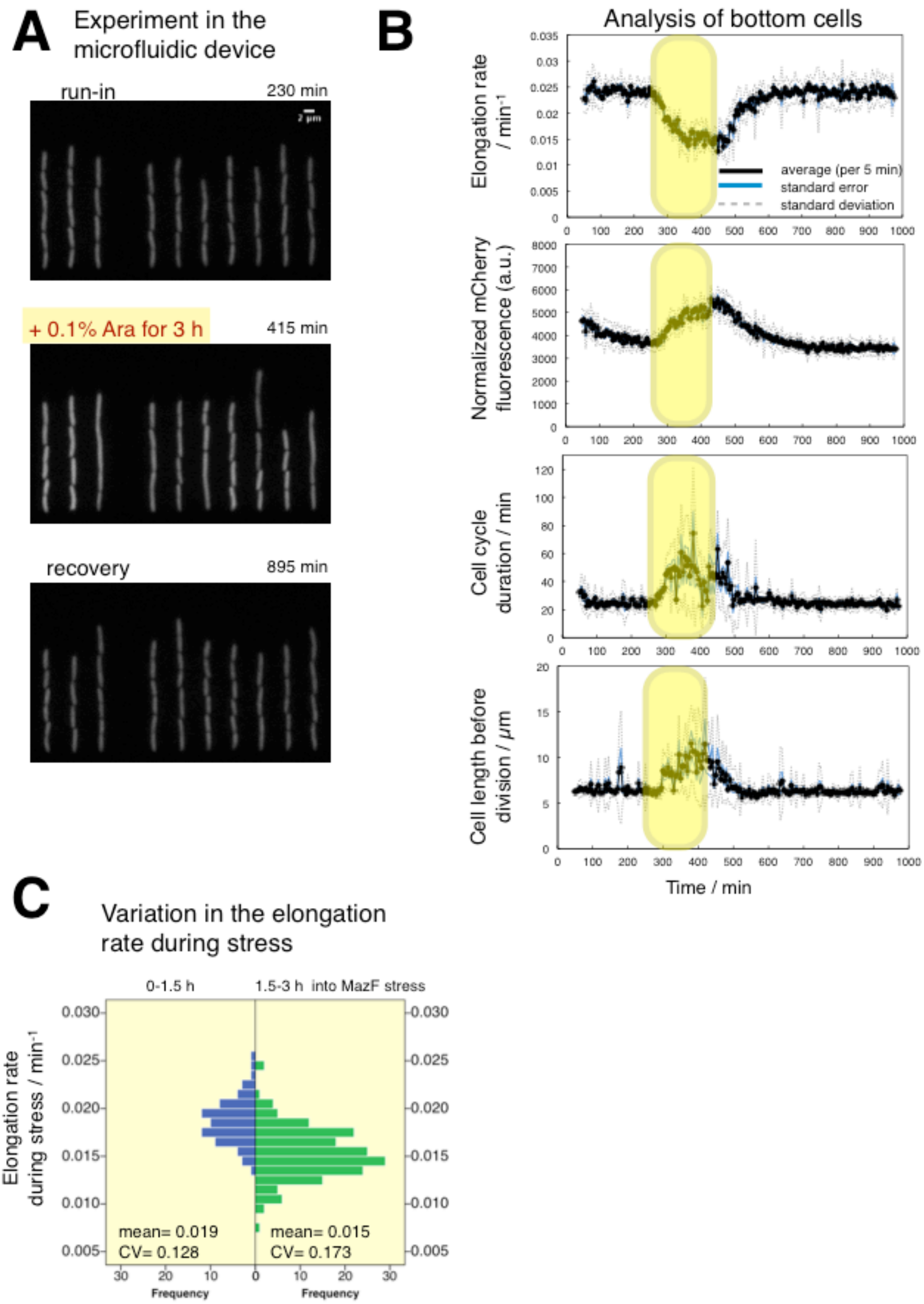

Figure S13. Time-lapse microscopy in a microfluidic device shows bacterial physiology before and during 3 hours of *mazF* overexpression, as well as recovery after stress caused by *mazF* overexpression.

(A) Cells of strain TB212 harboring pBAD-*mazF* were growing 4 hours to ensure steady-state conditions (time 0-240 min), then *mazF* expression was induced by switching to media with 0.1% Ara (time 245-420 min). The cells divided 3 or 4 times during 3 hours of the *mazF* expression. Recovery was monitored for 9 hours (time 425-1040 min). We analyzed 90 cells in the bottom of each microfluidic channel. We did not observe cell lysis during this experiment, and additionally 3 cells stopped dividing, which is 3.2% of the total bottom cell count. **(B)** A 3-hour window of ectopic *mazF* expression depicted in yellow indicates growth rate reduction. Single-cell growth is measured as the cell elongation rate and computed by exponential fitting the plot of cell length versus time, measured from cell birth until division. mCherry fluorescence increased due to the slow down of growth. Here plotted is 'normalized mCherry fluorescence', which is total fluorescence divided by the cell length. In all graphs the black line corresponds to sliding window average per 5 minutes of the time-lapse experiment. The blue line is standard error of the mean, and grey dashed line depicts standard deviation. Differences in the normalized mCherry fluorescence reflected changes in the cell elongation rate very well, Spearman's rho= -0.526, p-value= 0.00; R^2= 0.423; N= 2,462 cells. To determine if there are long-term effects of ectopic *mazF* expression, we applied Sign test for related samples for cells just before arabinose induction (one cell division prior the stress (i.e. 240th min of the experiment) and during recovery from stress (the cells' division between 800th-840th min). The differences were not statistically significant, p= 0.752 for cell length, p= 0.229 for cell cycle duration. **(C)** The bacterial population exhibited increased variation in the elongation rate during arabinose induction. The first stage, corresponding to first 90 minutes of stress (N= 69 cell analyzed, time 245-330 min), is characterized by rapid decrease in growth rate. In the second stage, corresponding to the remaining 90 minutes of stress period, the cells on average continued to grow slower, however, the growth rate between cells was more variable (N= 171 cells analyzed, time 335-420 min): variation in the elongation rate was 1.35 times higher in the second stage of arabinose induction than in the first stage.

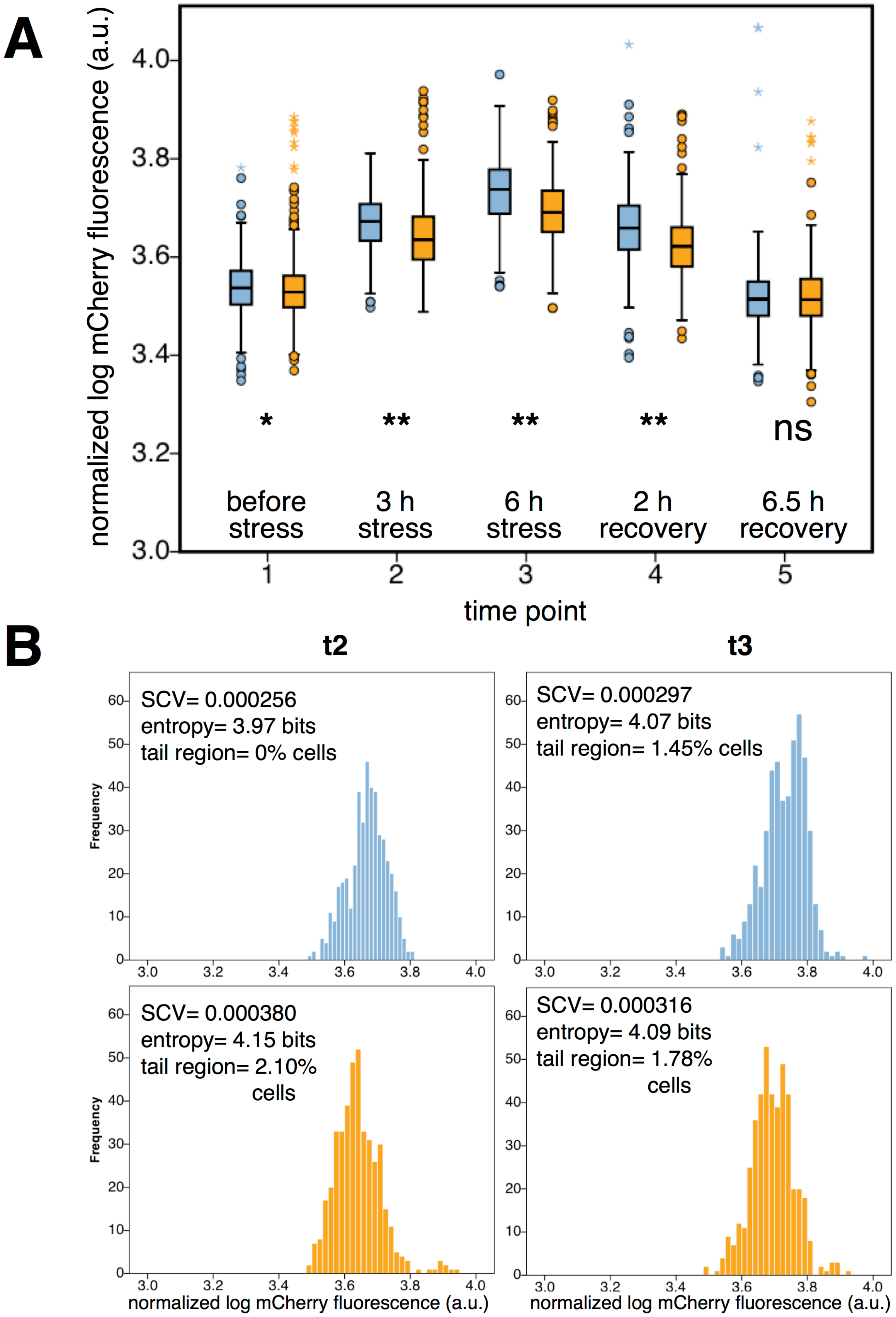

Figure S14. Analysis of different time points in the microfluidic experiment before, during and after arabinose induction.

We analyzed different frames (static images) of the microfluidic experiment. We collected five frames 36, 72, 108, 132 and 186 from each recorded position, that correspond to time of 180 min, 360 min, 540 min, 660 min and 930 min into the experiment, respectively. **(A)** There was no difference in the mCherry fluorescence between bacterial populations harboring pBAD-*mazF* (depicted in blue) and pBAD-*mazF*ΔACA (depicted in orange) in the full recovery phase (Mann-Whitney U test, p-value= 0.734; Kolmogorov-Smirnov test, p-value= 0.549). Slight differences were present in the beginning of the experiment, measured before arabinose induction (Mann-Whitney U test, p-value= 0.028; Kolmogorov-Smirnov test, p-value= 0.036). After 3 hours of arabinose induction, differences in the mCherry fluorescence were significant between conditions of *mazF* and *mazF*ΔACA overexpression (Mann-Whitney U test, p-value= 0.00; Kolmogorov-Smirnov test, p-value= 0.00). The differences were significant also after 6 hours of arabinose induction, as well as 2 hours into the recovery phase (Mann-Whitney U test, p-value= 0.00; Kolmogorov-Smirnov test, p-value= 0.00 – for both time points). **(B)** We observed multimodal patterns of mCherry fluorescence during arabinose induction. The variation (SCV) in mCherry fluorescence and the entropy (40 bins, range 3.4-4.0) were higher upon *mazF*ΔACA overexpression (orange) than upon *mazF* overexpression (blue). Also, a larger fraction of the population in the tail region (corresponding to the cells with $\log_{10}$ mCherry fluorescence > 3.85) was measured upon *mazF*ΔACA overexpression than upon *mazF* overexpression. The flow cytometry results show more distinctive patterns of multimodal mCherry fluorescence after 6 hours of arabinose induction than the results of the microfluidic experiments. One possible reason for this behavior is that cultures analyzed with flow cytometry were already in late exponential phase or at the onset to the stationary phase at the point of analysis (Figure S4A). During that growth phase, bacterial cultures exhibit changes in gene expression and an overall decrease in growth rate as a consequence of nutrient deprivation [Battesti et al. 2011]. Cells grow at steady state in microfluidic devices and do not experience shortage of nutrients [Wang et al. 2010].

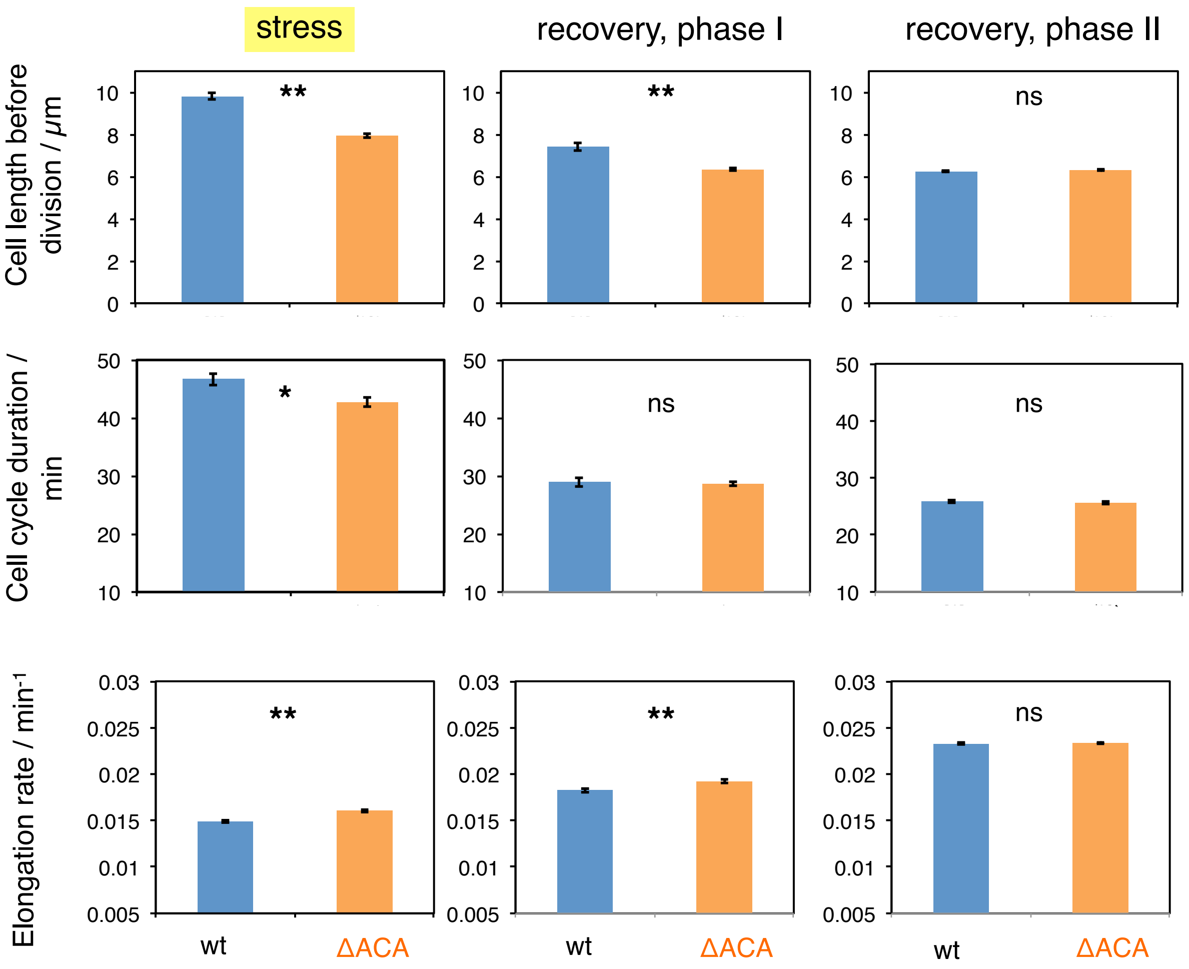

Figure S15. Phenotypic differences between the cells harboring pBAD-*mazF* and the cells harboring pBAD-*mazF*ΔACA.

We divided the dataset into 3 groups: (1) cells during arabinose induction (time 185-540 min, excluding the cells that experienced the switch to stress); cells in the first recovery phase i.e. exiting from stress (time 545-695 min, excluding the cells that experienced the switch to non-stressful conditions); (3) cells in the second recovery phase (700 min until the end) – and asked whether there are differences in the measured phenotypic traits if *mazF* was induced from plasmid pBAD-*mazF* (blue columns) or from plasmid pBAD-*mazF*ΔACA (orange columns). Error bars present standard error of the mean; ns stands for non significant, * stands for $p< 0.05$, ** stands for $p< 0.01$. During arabinose induction there were significant differences in cell length, elongation rate and cell cycle duration (Mann-Whitney U test, p-values 0.0, 0.0 and 0.01006, respectively). During the first recovery phase i.e. exit from stress caused by arabinose induction some differences were still present (Mann-Whitney U test, p-values 0.0, 0.0014 and 0.343, for cell length, elongation rate and cell cycle duration, respectively). The differences were completely absent in the second recovery phase (Mann-Whitney U test, p-values 0.083, 0.912 and 0.714, for cell length, elongation rate and cell cycle duration, respectively).

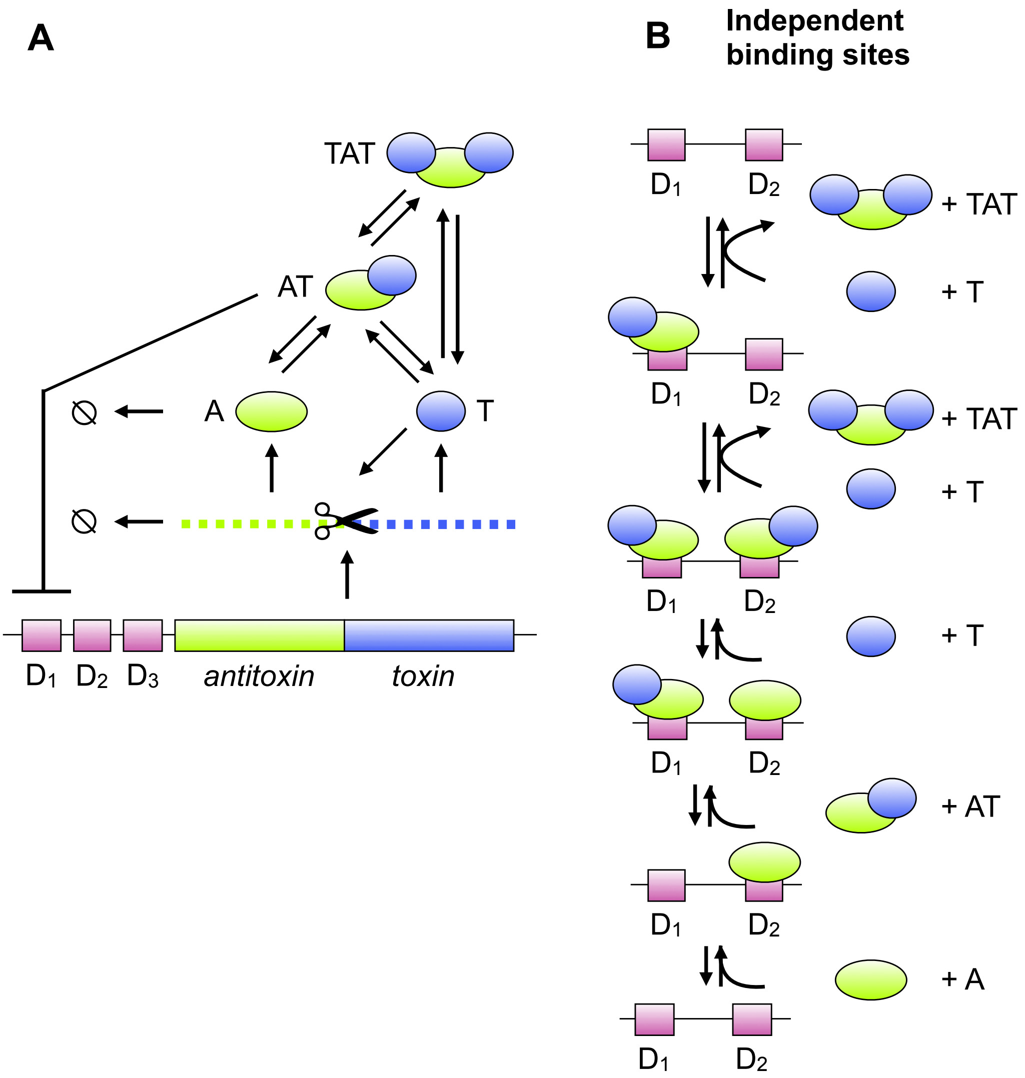

Figure S16. Model for the *mazEF* toxin-antitoxin module with three antitoxin-binding sites on the operator.

(A) The *mazEF* operon consists of a promoter/operator region, followed by the genes for the antitoxin and the toxin. After transcription of the polycistronic mRNA, the toxin and the antitoxin are translated. These proteins can form two non-toxic complexes, AT (transcriptional repressor) and TAT (de-represses TA transcription). The degradation of antitoxin and mRNA (represented by ⃠) is faster than the degradation of toxin and both complexes AT and TAT, in which case the degradation rate corresponds to dilution by cell division. **(B)** Molecular mechanism of conditional cooperativity in the 'independent binding sites' model used in this study. Two AT complexes can bind independently next to each other. Addition of a third toxin to form a TAT complex leads to release of this entity from the operator and de-repression [Gelens et al. 2013].

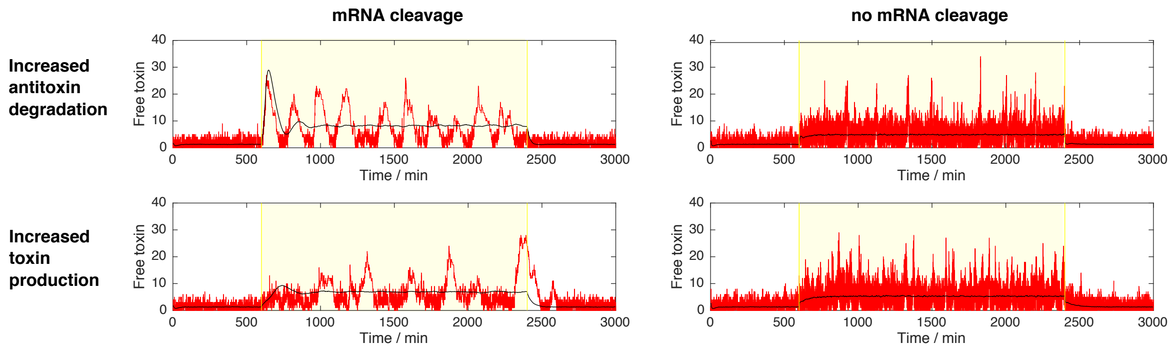

Figure S17. Numerical simulation of regulation of the *mazEF* toxin-antitoxin module expression in different stressors.

The system was simulated for 3,000 minutes per individual cell. The graphs in red show the results for the toxin level in a single cell. The average over 1,000 cells is indicated in black. Yellow window indicates the period of stress. In the first scenario (upper panels), the stressor was defined through enhanced degradation of the MazE antitoxin, which was in the model increased by a factor 3. In the second scenario (lower panels), we defined the stressor as elevated MazF production (similarly to our experimental setup), which was increased by a factor 5.

Regardless of the source of stress, MazF-dependent *mazEF* mRNA cleavage 1) triggered enlarged fluctuations in the MazF level in single cells during stress (red line), and 2) augmented initial spike in the MazF level upon entry to stress for the simulated population (average black line).

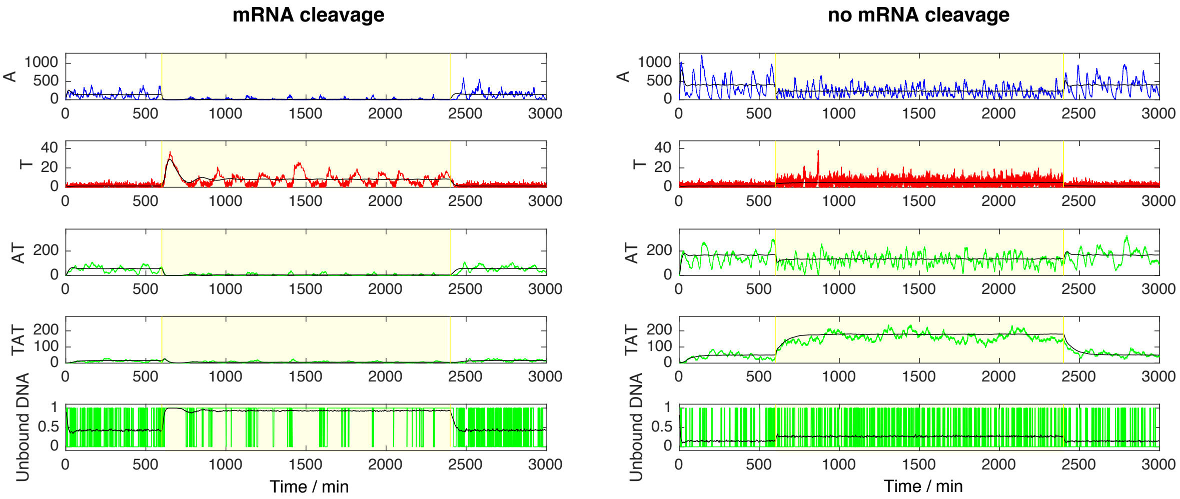

Figure S18. Numerical simulation of the *mazEF* toxin-antitoxin module, either including or excluding cleavage of the *mazEF* mRNA by MazF.

During the period of stress (indicated in yellow) the degradation of antitoxin was increased by a factor 3. The system was simulated for 3,000 minutes per individual cell. The graphs show the results for the levels of free antitoxin '**A'**, free toxin '**T'**, complex antitoxin-toxin '**AT'** (repressing complex) and complex toxin-antitoxin-toxin '**TAT'** (de-repressing complex), and the free operator DNA in a single cell '**Unbound DNA'**. The average values over 1,000 cells are indicated in black. The results of a single-cell simulation are shown in blue (A), red (T), and green (for AT, TAT and Unbound DNA).

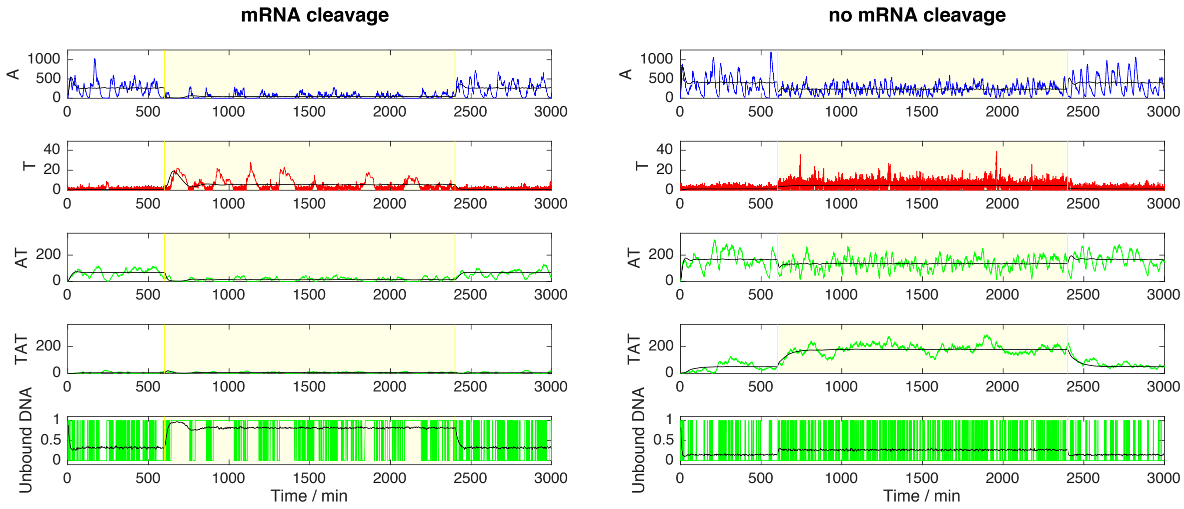

Figure S19. Numerical simulation of the *mazEF* toxin-antitoxin module, either including or excluding 'differential' cleavage of the *mazEF* mRNA by MazF.

During the period of stress (indicated in yellow) the degradation of antitoxin was increased by a factor 3. In these simulations *mazEF* mRNA is differentially cleaved: probability to cleave the *mazF* mRNA part is 4.5 times higher than the *mazE* mRNA part, given that the *mazF* coding sequence contains 9 ACA sites, and the *mazE* coding sequence contains 2 ACA sites [Yamaguchi and Inouye 2013]. The system was simulated for 3,000 minutes per individual cell. The graphs show the results for the levels of free antitoxin '**A**', free toxin '**T**', complex antitoxin-toxin '**AT**' (repressing complex) and complex toxin-antitoxin-toxin '**TAT**' (de-repressing complex), and the free operator DNA in a single cell '**Unbound DNA**'. The average values over 1,000 cells are indicated in black, and the results of a single-cell simulation are shown in blue (A), red (T), and green (for AT, TAT and Unbound DNA). These conditions of differential *mazEF* mRNA cleavage yielded higher levels of antitoxin and lower levels of toxin during stress, in comparison to the simulations presented in Figure S18 and Figure 6. However, the fluctuations in the level of toxin MazF persisted even during differential *mazEF* mRNA cleavage.

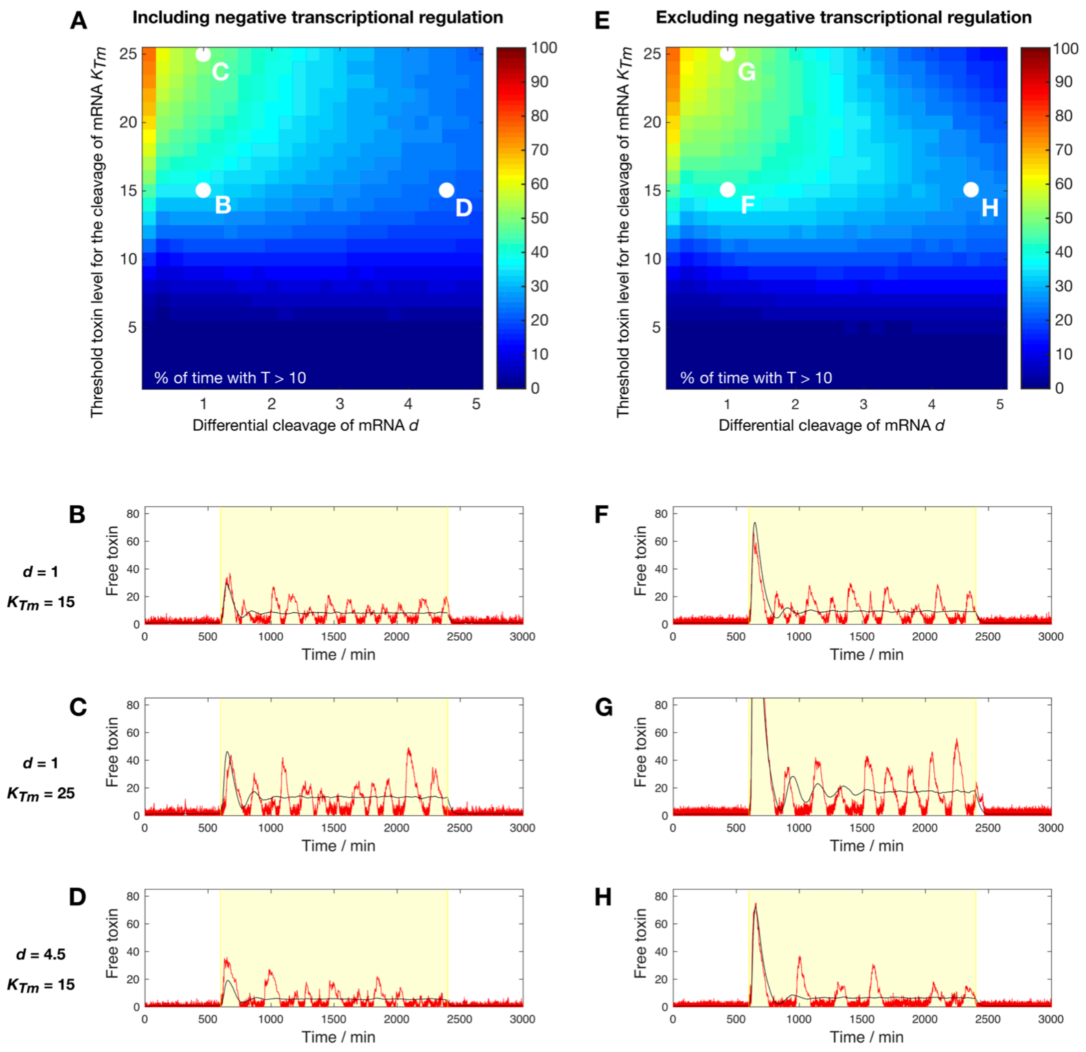

Figure S20. Influence of the negative transcriptional regulation, the threshold toxin level for the cleavage of mRNA and the differential cleavage of mRNA on the free toxin level.

(A) and **(E)** Parameter scan for K_{Tm} (the threshold toxin level for the cleavage of mRNA) versus d (the differential cleavage of toxin mRNA compared to antitoxin mRNA), showing the percentage of time that the toxin level is higher than 10 during a simulation of 5,000 hours, in which the degradation of antitoxin was increased by a factor 3, in **(A)** the full model or **(E)** the model excluding negative transcriptional regulation. **(B-D)** and **(F-H)** Individual simulations of the *mazEF* toxin-antitoxin module, including cleavage of the *mazEF* mRNA by MazF, for the parameters indicated by the white dots. During the period of stress (indicated in yellow) the degradation of antitoxin was increased by a factor 3. The system was simulated for 3,000 minutes per individual cell. The graphs show the results for the free toxin level in a single cell. The average over five hundred cells is indicated in black.

In general, this analysis shows that a higher threshold for mRNA degradation leads to higher free toxin levels (larger bursts of free toxin), while a higher relative cleavage of the part of the mRNA coding for the toxin leads to lower free toxin levels. The absence of negative transcriptional regulation in **(F-H)** gives rise to higher free toxin levels, while the overall dynamics (bursty toxin excitations) remains the same.

Supplementary Tables

Table S1. Strains and plasmids used in this study.

Strain or plasmid name	Relevant characteristics	Source or reference
MG1655	Wild-type *E. coli* K-12 F-, *λ-, ilvG-, rfb-50, rph-1*	[Blattner et al. 1997], #103
TOP10	Strain for plasmid propagation	#497
DH5α	Strain for plasmid propagation	#423
BW25142	Strain for CRIM plasmid propagation or propagation of R6K gamma origin plasmids in high copy number; F-, *Δ(araD-araB)567, ΔlacZ4787(::rrnB-3), Δ(phoB-phoR)580, λ-, galU95, ΔuidA4::pir-116, recA1, endA9(del-ins)::*frt, *rph-1, Δ(rhaD-rhaB)568, hsdR514*	[Haldimann and Wanner 2001], #1055
BW27784	used for arabinose-inducible expression; *Δ(araD-araB)567, ΔlacZ4787(::rrnB-3), λ-, Δ(araH-araF)570(::FRT), ΔaraEp-532::*frt, *ϕPcp18araE533, Δ(rhaD-rhaB)568, hsdR514*	[Khlebnikov et al. 2000], #1056
TB212	BW27784 *att*P21::λP_R-*mCherry*::frt; constitutive *mCherry* expression	[Bergmiller et al. 2017], #1058
TB164	MG1655 *att*λ::(*lacIq tetR* SpecR)	made by Tobias Bergmiller
MG1655 *lacIq tetR*	MG1655 *att*λ::(*lacIq tetR* SpecR); P1-transduced [Miller JH 1972] TB164 into MG1655 #103	This study, #1347
MG1655 *ΔmazF*	P1-transduced KEIO collection Δ*mazF* strain into MG1655, and removed Kan-cassette with Flp recombinase	[Müller et al. 2016], #888
MG1655 LVM	Wild-type *E. coli* K-12 from LVM Lab	[Tsilibaris et al. 2007], gift from Laurence Van Melderen, #1287
LVM100	MG1655 Δ*mazEF* Δ*relBEF* Δ*chpB* Δ*yefM-yoeB* Δ*dinJ-yafQ*	[Tsilibaris et al. 2007], gift from Laurence Van Melderen, #1288
LVM101	MG1655 Δ*mazEF*	[Tsilibaris et al. 2007], gift from Laurence Van Melderen, #1289
NN204-cat	BW27784 *att*λ::P_{BAD}-*mazF*::frt-*cat*	This study, #1081
NN205-cat	TB212 *att*λ::P_{BAD}-*mazF*::frt-*cat*	This study, #1082
NN209-cat	BW27784 *att*λ::P_{BAD}-Em*gfp*ΔACA::frt-*cat*	This study, #1110
NN210-cat	TB212 *att*λ::P_{BAD}-Em*gfp*ΔACA::frt-*cat*	This study, #1111
NN204	BW27784 *att*λ::P_{BAD}-*mazF*::frt	This study, #1090
NN205	TB212 *att*λ::P_{BAD}-*mazF*::frt	This study, #1091
NN209	BW27784 *att*λ::P_{BAD}-Em*gfp*ΔACA::frt	This study, #1122
NN210	TB212 *att*λ::P_{BAD}-Em*gfp*ΔACA::frt	This study, #1123
pAH120-frt-chlor	pAH120 Δ*bla*::frt-*cat*-frt	made by Tobias Bergmiller, Guet Strain Collection, #1067
pINT-ts	helper plasmid for integration in *att*λ	[Haldimann and Wanner 2001], #1068
pCP20	AmpR, CamR, yeast Flp recombinase activity; *flp, bla, cat, rep101ts*	[Cherepanov and Wackernagel 1995], #1064
pZS*-mCherry	KanR, SC101* ori, λP_R-*mCherry*; constitutive *mCherry* expression	Guet Strain Collection, #1234
pZS*12-GFP	AmpR, SC101* ori	Guet Strain Collection, #1059
pMS2_612	AmpR, pUH-C w/o 2nd operator; constitutive Em*gfp*ΔACA expression	[Sauert 2015], #884
pZS*-612	AmpR, SC101* ori, HindIII/XhoI digestion of insert from pMS2_612 to pZS*12-GFP; constitutive Em*gfp*ΔACA expression	This study, #1061
pNN204	CamR, P_{BAD}-*mazF*; backbone CRIM plasmid pAH120-frt-cat, PstI/(BamHI-BglII disrupted)	This study, #1070
pNN209	CamR, P_{BAD}-Em*gfp*ΔACA; backbone CRIM plasmid pAH120-frt-cat, PstI/(BamHI-BglII disrupted)	This study, #1071
pNN204-ZS*	AmpR, P_{BAD}-*mazF*; backbone pZS*12-GFP, XhoI/HindIII	This study, #1075
pNN209-ZS*	AmpR, P_{BAD}-Em*gfp*ΔACA; backbone pZS*12-GFP, XhoI/HindIII; the same construction as P_{BAD}-*mazF*, then *mazF* swapped via KpnI/HindIII with Em*gfp*ΔACA	This study, #1076
pSA1-*mazF*	AmpR, P_{T5}-*mazF, lacIq*; IPTG-inducible *mazF* expression	[Amitai et al. 2009], #691
pBAD33	CamR, p15A ori, w/o ribosome binding site; Ara-inducible expression of a gene of interest	#681
pBAD-*mazF*	CamR, p15A ori, pBAD33 backbone, P_{BAD}-*mazF*; Ara-inducible *mazF* expression	[Amitai et al. 2004], #692
pBAD-*mazF*ΔACA	CamR, p15A ori, pBAD33 backbone, *mazF* w/o ACA sites synthesized by IDT; Ara-inducible *mazF*ΔACA expression	This study, #1275
pBAD-$mazF_{E24A}$	CamR, p15A ori, pBAD33 backbone, MazF(E24A) is an inactive version of MazF; Ara-inducible *mazF*(E24A) expression	This study, made according to [Tripathi et al. 2014], #1180

(# is the position in the Moll Strain Collection)

Table S2. List of primers and probes for RNA biochemistry and qPCR.

Name	Sequence	Used for
N46	5'-GCTAGCCCAAAAAAACGGG-3'	*mazF* mRNA detection (Figure 2BC)
F47	5'-<u>TAATACGACTCACTATTAGGC</u>GTTTTTTTGGGCTAGC-3'	*In vitro* transcription, T7 promoter sequence underlined [Coleman et al. 2004] (Figure 2BC)
L56	5'-GACCGGATAAAACAACTTCGAACG-3'	*mazF* mRNA primer extension (Figure 2D)
N56	5'-CGCTCATATTGCTCTACGATCG-3'	*mCherry* mRNA detection (Figure S6)
M38	5'-GGTAACGTCAATGAGCAAAGG-3'	16S rRNA detection (Figure S12)
T55	5'-GCTGCCTCCCGTAGGAGT-3'	16S rRNA detection, probe 16S_P1 from [Mets et al. 2017] (Figure S12)
mazF_for1	5'-CTCTAGAGGAGTGAAACGAT-3'	*mazF* mRNA half-life, amplification of the A region without ACA sites (Figure 4E)
mazF_rev1	5'-TCAACCCAAATCAGATCGC-3'	*mazF* mRNA half-life, amplification of the A region without ACA sites (Figure 4E)
mazF_for2	5'-GGTATGTGTCTGTGTGTTCCTT-3'	*mazF* mRNA half-life, amplification of the B region around 7th ACA site [Venturelli et al. 2017] (Figure 4E)
mazF_rev2	5'-CCTTTCTTCGTTGCTCCTCTT-3'	*mazF* mRNA half-life, amplification of the B region around 7th ACA site [Venturelli et al. 2017] (Figure 4E)
cysG_fwd	5'-TCTACGACCGTCTGGTTTCT-3'	*cysG* mRNA half-life [Venturelli et al. 2017] (Figure 4E)
cysG_rev	5'-CGCAGCAGGATCTGGTTAAT-3'	*cysG* mRNA half-life [Venturelli et al. 2017] (Figure 4E)
pBAD	5'-FAM-GCCGATACGTACCCGATATGG-BHQ1-3'	qPCR probe for the *mazF* transcript; probe for the amplified A region (Figure 4E)
mazF	5'-FAM-CGTGATGGCGTAGCGTTAGCTGAT-BHQ1-3'	qPCR probe for the *mazF* transcript; probe for the amplified B region around 7th ACA site (Figure 4E)
cysG	5'-FAM-TATGAATCTGGTACGCCGCGATGC-BHQ1-3'	qPCR probe for the *cysG* transcript [Venturelli et al. 2017] (Figure 4E)

Table S3. Maximum growth rate and recovery time from stress.

	Maximum growth rate / per h		Doubling time / min	
	control	recovery after arabinose induction	control	recovered cultures
wild-type	*1.69 ± 0.057	1.40 ± 0.075	24.7 ± 0.82	29.9 ± 1.65
Δ*mazEF*	1.54 ± 0.044	1.51 ± 0.034	27.1 ± 0.80	27.6 ± 0.63
Δ5	1.40 ± 0.022	1.24 ± 0.048	29.7 ± 0.45	33.7 ± 0.88

	Exit from lag-phase / min		
	control	after arabinose induction	~(after arabinose induction - control)
wild-type	76.7 ± 2.72	346.7 ± 11.86	270.0
Δ*mazEF*	123.3 ± 7.20	456.7 ± 16.56	333.4
Δ5	140.0 ± 12.47	680.0 ± 21.21	540.0

* mean ± standard error of the mean

Table S4. Shannon entropy as the measure of fluorescence heterogeneity, and quantification of the fraction of the population in tail region, both for mCherry fluorescence encoded on the chromosome and for GFP fluorescence encoded on plasmid pZS*-612.

			Shannon entropy / bit		Percentage of the population in the *tail	
Time point	Strain	Replicate	mCherry	GFP	mCherry	GFP
exponential phase	TB212 pBAD-*mazF* pZS*-612	# 1	[1]3.181	[2]3.002	0.008	0.006
		# 2	3.120	2.954	0.022	0.020
		# 3	3.046	2.894	0.004	0.016
		# 4	3.062	2.923	0.004	0.016
	TB212 pBAD-*mazF*ΔACA pZS*-612	# 1	[1]3.191	[2]3.036	0.014	0.010
		# 2	3.084	2.920	0.014	0.008
		# 3	3.127	2.971	0.010	0.008
		# 4	3.086	2.978	0.002	0.000
6 h 15 min after induction with 0.1% Ara	TB212 pBAD-*mazF* pZS*-612	# 1	[3]3.425	[4]3.745	0.034	6.228
		# 2	3.441	3.783	0.050	6.892
		# 3	3.471	3.758	0.124	5.836
		# 4	3.280	3.723	0.018	3.546
	TB212 pBAD-*mazF*ΔACA pZS*-612	# 1	[3]4.007	[4]4.244	5.412	19.520
		# 2	4.046	4.201	9.306	23.566
		# 3	3.940	4.178	6.596	27.150
		# 4	3.815	4.212	2.514	19.284

* Fluorescence values > 2,000 a.u.
[1] exponential phase, differences in Shannon entropy in mCherry fluorescence not significant, N= 4, *t*-test, p-value= 0.639
[2] exponential phase, differences in Shannon entropy in GFP fluorescence not significant, N= 4, *t*-test, p-value= 0.355
[3] after Ara induction, differences in Shannon entropy in mCherry fluorescence significant, N= 4, *t*-test, p-value= 0.0001936
[4] after Ara induction, differences in Shannon entropy in GFP fluorescence significant, N= 4, *t*-test, p-value= 3.20E-07

Table S5. Parameter set for modeling *mazEF* regulation.

Parameter	Description	***mazEF*** **module**	Units
**Main model parameters**			
ρ_U	Unbound mRNA transcription rate	0.121	s^{-1}
ρ_B	Bound mRNA transcription rate	0	s^{-1}
β_A	Antitoxin translation rate	0.122	s^{-1}
β_T	Toxin translation rate	0.009	s^{-1}
V	Volume factor	$3.612 \cdot 10^{8}$	m^3
$d_{m,0}$	mRNA decay rate in the absence of MazF	0.00203	s^{-1}
d_c	Decay rate due to cell cycle dilution	$2.888 \cdot 10^{-4}$	s^{-1}
d_A	Antitoxin decay rate	$8 \cdot d_c$	s^{-1}
F	Decay of antitoxin inside the complex	$0.1 \cdot d_A$	s^{-1}
α_{TH}	Binding of antitoxin and toxin through the high affinity site	$1.32 \cdot 10^{5}$	$M^{-1}s^{-1}$
θ_{TH}	Unbinding of antitoxin and toxin through the high affinity site	$2.78 \cdot 10^{-4}$	s^{-1}
α_{AT}	Binding of complex AT to binding site on the operator	4620	$M^{-1}s^{-1}$
θ_{AT}	Unbinding of complex AT from a binding site on the operator	0.00231	s^{-1}
α_A	Binding of antitoxin to a binding site on the operator	4620	$M^{-1}s^{-1}$
θ_A	Unbinding of antitoxin from a binding site on the operator	0.0231	s^{-1}
K_{Tm}	Threshold toxin level for the cleavage of mRNA	15	
n_m	Hill factor describing the sharpness of the transition around K_T for the cleavage of mRNA	3	
$d_{m,max}$	Maximal mRNA cleavage rate in the presence of MazF	10	s^{-1}
**Parameters used to generate histograms of the division times based on the free toxin levels**			
$t_{D,0}$	Cell division time in the absence of MazF	2400	s
K_{TD}	Threshold toxin level for the influence on the cell division time	5	
n_D	Hill factor describing the sharpness of the transition around K_T for the influence on the cell division time	5	
$t_{D,max}$	Maximal cell division time in the presence of MazF	6000	s
D	Noise level	0.1	

Supplementary Movies

Movie S1. Time-lapse of single cells during *mazF* overexpression. (Scale bar corresponds to 5 µm.)

Movie S2. Time-lapse of single cells during *mazF*ΔACA overexpression.

Sequence Alignments

Sequence S1. Sequence alignment of P_{BAD}-*mazF* with sequenced plasmids from single colonies.
After 6 hours of induction from pBAD-*mazF*, dilution series of cultures were plated and incubated overnight at 37°C. Colonies were counted and these results are reported in Figure 1B and Figure 3B. 'MG' stands for MG1655, and 'TB' stands for TB212 – both strains harboring pBAD-*mazF*. Colonies 'A', 'B', 'D' were then picked and inoculated overnight in LB supplemented with 15 µg/ml chloramphenicol, and mini-prep was performed the following day. Colonies 'C' were formed on the plates after additional overnight incubation at room temperature. Samples are named as Strain-#Replicate-#DilutionSeriesPlate-Colony, and sequenced by using standard reverse primer BAD-HisA-R GTTTTATCAGACCGCTTCTG (LGC Genomics).

```
*

PBADmazF_rev    1 ------------------------------CTACCCAATCAGTACGTTAATTTTGGCTTTAATGAGTTGTAATTCCTCTG
MG_1_1_A        1 CTTCTCTCATCCGCCAAAACAGCCAAGCTTCTACCCAATCAGTACGTTAATTTTGGCTTTAATGAGTTGTAATTCCTCTG
MG_1_1_B        1 -TTCTCTCATCCGCCAAAACAGCCAAGCTTCTACCCAATCAGTACGTTAATTTTGGCTTTAATGAGTTGTAATTCCTCTG
MG_1_1_C        1 CTTCTCTCATCCGCCAAAACAGCCAAGCTTCTACCCAATCAGTACGTTAATTTTGGCTTTAATGAGTTGTAATTCCTCTG
MG_2_1_A        1 CTTCTCTCATCCGCCAAAACAGCCAAGCTTCTACCCAATCAGTACGTTAATTTTGGCTTTAATGAGTTGTAATTCCTCTG
MG_2_1_B        1 ----------------------------------------------------------------AGTTGTAATTCCTCTG
MG_2_1_C        1 ----------------------------------------------------------------AGTTGTAATTCCTCTG
MG_3_1_A        1 CTTCTCTCATCCGCCAAAACAGCCAAGCTTCTACCCAATCAGTACGTTAATTTTGGCTTTAATGAGTTGTAATTCCTCTG
MG_3_1_B        1 --------------------------GCTTCTACCCAATCAGTACGTTAATTTTGGCTTTAATGAGTTGTAATTCCTCTG
MG_3_1_C        1 CTTCTCTCATCCGCCAAAACAGCCAAGCTTCTACCCAATCAGTACGTTAATTTTGGCTTTAATGAGTTGTAATTCCTCTG
TB_2_3_A        1 CTTCTCTCATCCGCCAAAACAGCCAAGCTTCTACCCAATCAGTACGTTAATTTTGGCTTTAATGAGTTGTAATTCCTCTG
TB_2_3_B        1 --TCTCTCATCCGCCAAAACAGCCAAGCTTCTACCCAATCAGTACGTTAATTTTGGCTTTAATGAGTTGTAATTCCTCTG
TB_2_3_C        1 CTTCTCTCATCCGCCAAAACAGCCAAGCTTCTACCCAATCAGTACGTTAATTTTGGCTTTAATGAGTTGTAATTCCTCTG
TB_2_3_D        1 CTTCTCTCATCCGCCAAAACAGCCAAGCTTCTACCCAATCAGTACGTTAATTTTGGCTTTAATGAGTTGTAATTCCTCTG
TB_3_3_C        1 CTTCTCTCATCCGCCAAAACAGCCAAGCTTCTACCCAATCAGTACGTTAATTTTGGCTTTAATGAGTTGTAATTCCTCTG
TB_4_3_A        1 -TTCTCTCATCCGCCAAAACAGCCAAGCTTCTACCCAATCAGTACGTTAATTTTGGCTTTAATGAGTTGTAATTCCTCTG
TB_4_3_B        1 CTTCTCTCATCCGCCAAAACAGCCAAGCTTCTACCCAATCAGTACGTTAATTTTGGCTTTAATGAGTTGTAATTCCTCTG
TB_4_3_C        1 CTTCTCTCATCCGCCAAAACAGCCAAGCTTCTACCCAATCAGTACGTTAATTTTGGCTTTAATGAGTTGTAATTCCTCTG

PBADmazF_rev   51 GGGCAACTGTTCCTTTCTTCGTTGCTCCTCTTGCCCGCCAGGCGATACTTTTTACCTGATCAGCTAACGCTACGCCATCA
MG_1_1_A       81 GGGCAACTGTTCCTTTCTTCGTTGCTCCTCTTGCCCGCCAGGCGATACTTTTTACCTGATCAGCTAACGCTACGCCATCA
MG_1_1_B       80 GGGCAACTGTTCCTTTCTTCGTTGCTCCTCTTGCCCGCCAGGCGATACTTTTTACCTGATCAGCTAACGCTACGCCATCA
MG_1_1_C       81 GGGCAACTGTTCCTTTCTTCGTTGCTCCTCTTGCCCGCCAGGCGATACTTTTTACCTGATCAGCTAACGCTACGCCATCA
MG_2_1_A       81 GGGCAACTGTTCCTTTCTTCGTTGCTCCTCTTGCCCGCCAGGCGATACTTTTTACCTGATCAGCTAACGCTACGCCATCA
MG_2_1_B       17 GGGCAACTGTTCCTTTCTTCGTTGCTCCTCTTGCCCGCCAGGCGATACTTTTTACCTGATCAGCTAACGCTACGCCATCA
MG_2_1_C       17 GGGCAACTGTTCCTTTCTTCGTTGCTCCTCTTGCCCGCCAGGCGATACTTTTTACCTGATCAGCTAACGCTACGCCATCA
MG_3_1_A       81 GGGCAACTGTTCCTTTCTTCGTTGCTCCTCTTGCCCGCCAGGCGATACTTTTTACCTGATCAGCTAACGCTACGCCATCA
MG_3_1_B       55 GGGCAACTGTTCCTTTCTTCGTTGCTCCTCTTGCCCGCCAGGCGATACTTTTTACCTGATCAGCTAACGCTACGCCATCA
MG_3_1_C       81 GGGCAACTGTTCCTTTCTTCGTTGCTCCTCTTGCCCGCCAGGCGATACTTTTTACCTGATCAGCTAACGCTACGCCATCA
TB_2_3_A       81 GGGCAACTGTTCCTTTCTTCGTTGCTCCTCTTGCCCGCCAGGCGATACTTTTTACCTGATCAGCTAACGCTACGCCATCA
TB_2_3_B       79 GGGCAACTGTTCCTTTCTTCGTTGCTCCTCTTGCCCGCCAGGCGATACTTTTTACCTGATCAGCTAACGCTACGCCATCA
TB_2_3_C       81 GGGCAACTGTTCCTTTCTTCGTTGCTCCTCTTGCCCGCCAGGCGATACTTTTTACCTGATCAGCTAACGCTACGCCATCA
TB_2_3_D       81 GGGCAACTGTTCCTTTCTTCGTTGCTCCTCTTGCCCGCCAGGCGATACTTTTTACCTGATCAGCTAACGCTACGCCATCA
TB_3_3_C       81 GGGCAACTGTTCCTTTCTTCGTTGCTCCTCTTGCCCGCCAGGCGATACTTTTTACCTGATCAGCTAACGCTACGCCATCA
TB_4_3_A       80 GGGCAACTGTTCCTTTCTTCGTTGCTCCTCTTGCCCGCCAGGCGATACTTTTTACCTGATCAGCTAACGCTACGCCATCA
TB_4_3_B       81 GGGCAACTGTTCCTTTCTTCGTTGCTCCTCTTGCCCGCCAGGCGATACTTTTTACCTGATCAGCTAACGCTACGCCATCA
TB_4_3_C       81 GGGCAACTGTTCCTTTCTTCGTTGCTCCTCTTGCCCGCCAGGCGATACTTTTTACCTGATCAGCTAACGCTACGCCATCA

PBADmazF_rev  131 CGTTCCTGACCGGATAAAACAACTTCGAACGGATATCCTTTTGATTGCGTTGTACAAGGAACACACAGACACATACCTGT
MG_1_1_A      161 CGTTCCTGACCGGATAAAACAACTTCGAACGGATATCCTTTTGATTGCGTTGTACAAGGAACACACAGACACATACCTGT
MG_1_1_B      160 CGTTCCTGACCGGATAAAACAACTTCGAACGGATATCCTTTTGATTGCGTTGTACAAGGAACACACAGACACATACCTGT
MG_1_1_C      161 CGTTCCTGACCGGATAAAACAACTTCGAACGGATATCCTTTTGATTGCGTTGTACAAGGAACACACAGACACATACCTGT
MG_2_1_A      161 CGTTCCTGACCGGATAAAACAACTTCGAACGGATATCCTTTTGATTGCGTTGTACAAGGAACACACAGACACATACCTGT
MG_2_1_B       97 CGTTCCTGACCGGATAAAACAACTTCGAACGGATATCCTTTTGATTGCGTTGTACAAGGAACACACAGACACATACCTGT
MG_2_1_C       97 CGTTCCTGACCGGATAAAACAACTTCGAACGGATATCCTTTTGATTGCGTTGTACAAGGAACACACAGACACATACCTGT
MG_3_1_A      161 CGTTCCTGACCGGATAAAACAACTTCGAACGGATATCCTTTTGATTGCGTTGTACAAGGAACACACAGACACATACCTGT
MG_3_1_B      135 CGTTCCTGACCGGATAAAACAACTTCGAACGGATATCCTTTTGATTGCGTTGTACAAGGAACACACAGACACATACCTGT
MG_3_1_C      161 CGTTCCTGACCGGATAAAACAACTTCGAACGGATATCCTTTTGATTGCGTTGTACAAGGAACACACAGACACATACCTGT
TB_2_3_A      161 CGTTCCTGACCGGATAAAACAACTTCGAACGGATATCCTTTTGATTGCGTTGTACAAGGAACACACAGACACATACCTGT
TB_2_3_B      159 CGTTCCTGACCGGATAAAACAACTTCGAACGGATATCCTTTTGATTGCGTTGTACAAGGAACACACAGACACATACCTGT
TB_2_3_C      161 CGTTCCTGACCGGATAAAACAACTTCGAACGGATATCCTTTTGATTGCGTTGTACAAGGAACACACAGACACATACCTGT
TB_2_3_D      161 CGTTCCTGACCGGATAAAACAACTTCGAACGGATATCCTTTTGATTGCGTTGTACAAGGAACACACAGACACATACCTGT
TB_3_3_C      161 CGTTCCTGACCGGATAAAACAACTTCGAACGGATATCCTTTTGATTGCGTTGTACAAGGAACACACAGACACATACCTGT
TB_4_3_A      160 CGTTCCTGACCGGATAAAACAACTTCGAACGGATATCCTTTTGATTGCGTTGTACAAGGAACACACAGACACATACCTGT
TB_4_3_B      161 CGTTCCTGACCGGATAAAACAACTTCGAACGGATATCCTTTTGATTGCGTTGTACAAGGAACACACAGACACATACCTGT
TB_4_3_C      161 CGTTCCTGACCGGATAAAACAACTTCGAACGGATATCCTTTTGATTGCGTTGTACAAGGAACACACAGACACATACCTGT

PBADmazF_rev  211 TTTGTTGTTGTACATGAAAGGACTCAGGACAACAGCTGGACGATGTCCAGCTTGCTCGCTACCTTTTGTCGGGTCAAAAT
MG_1_1_A      241 TTTGTTGTTGTACATGAAAGGACTCAGGACAACAGCTGGACGATGTCCAGCTTGCTCGCTACCTTTTGTCGGGTCAAAAT
MG_1_1_B      240 TTTGTTGTTGTACATGAAAGGACTCAGGACAACAGCTGGACGATGTCCAGCTTGCTCGCTACCTTTTGTCGGGTCAAAAT
MG_1_1_C      241 TTTGTTGTTGTACATGAAAGGACTCAGGACAACAGCTGGACGATGTCCAGCTTGCTCGCTACCTTTTGTCGGGTCAAAAT
MG_2_1_A      241 TTTGTTGTTGTACATGAAAGGACTCAGGACAACAGCTGGACGATGTCCAGCTTGCTCGCTACCTTTTGTCGGGTCAAAAT
MG_2_1_B      177 TTTGTTGTTGTACATGAAAGGACTCAGGACAACAGCTGGACGATGTCCAGCTTGCTCGCTACCTTTTGTCGGGTCAAAAT
MG_2_1_C      177 TTTGTTGTTGTACATGAAAGGACTCAGGACAACAGCTGGACGATGTCCAGCTTGCTCGCTACCTTTTGTCGGGTCAAAAT
MG_3_1_A      241 TTTGTTGTTGTACATGAAAGGACTCAGGACAACAGCTGGACGATGTCCAGCTTGCTCGCTACCTTTTGTCGGGTCAAAAT
MG_3_1_B      215 TTTGTTGTTGTACATGAAAGGACTCAGGACAACAGCTGGACGATGTCCAGCTTGCTCGCTACCTTTTGTCGGGTCAAAAT
MG_3_1_C      241 TTTGTTGTTGTACATGAAAGGACTCAGGACAACAGCTGGACGATGTCCAGCTTGCTCGCTACCTTTTGTCGGGTCAAAAT
TB_2_3_A      241 TTTGTTGTTGTACATGAAAGGACTCAGGACAACAGCTGGACGATGTCCAGCTTGCTCGCTACCTTTTGTCGGGTCAAAAT
TB_2_3_B      239 TTTGTTGTTGTACATGAAAGGACTCAGGACAACAGCTGGACGATGTCCAGCTTGCTCGCTACCTTTTGTCGGGTCAAAAT
TB_2_3_C      241 TTTGTTGTTGTACATGAAAGGACTCAGGACAACAGCTGGACGATGTCCAGCTTGCTCGCTACCTTTTGTCGGGTCAAAAT
TB_2_3_D      241 TTTGTTGTTGTACATGAAAGGACTCAGGACAACAGCTGGACGATGTCCAGCTTGCTCGCTACCTTTTGTCGGGTCAAAAT
TB_3_3_C      241 TTTGTTGTTGTACATGAAAGGACTCAGGACAACAGCTGGACGATGTCCAGCTTGCTCGCTACCTTTTGTCGGGTCAAAAT
TB_4_3_A      240 TTTGTTGTTGTACATGAAAGGACTCAGGACAACAGCTGGACGATGTCCAGCTTGCTCGCTACCTTTTGTCGGGTCAAAAT
TB_4_3_B      241 TTTGTTGTTGTACATGAAAGGACTCAGGACAACAGCTGGACGATGTCCAGCTTGCTCGCTACCTTTTGTCGGGTCAAAAT
TB_4_3_C      241 TTTGTTGTTGTACATGAAAGGACTCAGGACAACAGCTGGACGATGTCCAGCTTGCTCGCTACCTTTTGTCGGGTCAAAAT
```

```
PBADmazF_rev  291 CAACCCAAATCAGATCGCCCATATCGGGTACGTATCGGCTTACCATCGTTTCACTCCTCTAGAGGATCCCCGGGTACCGA
MG_1_1_A      321 CAACCCAAATCAGATCGCCCATATCGGGTACGTATCGGCTTACCATCGTTTCACTCCTCTAGAGGATCCCCGGGTACCGA
MG_1_1_B      320 CAACCCAAATCAGATCGCCCATATCGGGTACGTATCGGCTTACCATCGTTTCACTCCTCTAGAGGATCCCCGGGTACCGA
MG_1_1_C      321 CAACCCAAATCAGATCGCCCATATCGGGTACGTATCGGCTTACCATCGTTTCACTCCTCTAGAGGATCCCCGGGTACCGA
MG_2_1_A      321 CAACCCAAATCAGATCGCCCATATCGGGTACGTATCGGCTTACCATCGTTTCACTCCTCTAGAGGATCCCCGGGTACCGA
MG_2_1_B      257 CAACCCAAATCAGATCGCCCATATCGGGTACGTATCGGCTTACCATCGTTTCACTCCTCTAGAGGATCCCCGGGTACCGA
MG_2_1_C      257 CAACCCAAATCAGATCGCCCATATCGGGTACGTATCGGCTTACCATCGTTTCACTCCTCTAGAGGATCCCCGGGTACCGA
MG_3_1_A      321 CAACCCAAATCAGATCGCCCATATCGGGTACGTATCGGCTTACCATCGTTTCACTCCTCTAGAGGATCCCCGGGTACCGA
MG_3_1_B      295 CAACCCAAATCAGATCGCCCATATCGGGTACGTATCGGCTTACCATCGTTTCACTCCTCTAGAGGATCCCCGGGTACCGA
MG_3_1_C      321 CAACCCAAATCAGATCGCCCATATCGGGTACGTATCGGCTTACCATCGTTTCACTCCTCTAGAGGATCCCCGGGTACCGA
TB_2_3_A      321 CAACCCAAATCAGATCGCCCATATCGGGTACGTATCGGCTTACCATCGTTTCACTCCTCTAGAGGATCCCCGGGTACCGA
TB_2_3_B      319 CAACCCAAATCAGATCGCCCATATCGGGTACGTATCGGCTTACCATCGTTTCACTCCTCTAGAGGATCCCCGGGTACCGA
TB_2_3_C      321 CAACCCAAATCAGATCGCCCATATCGGGTACGTATCGGCTTACCATCGTTTCACTCCTCTAGAGGATCCCCGGGTACCGA
TB_2_3_D      321 CAACCCAAATCAGATCGCCCATATCGGGTACGTATCGGCTTACCATCGTTTCACTCCTCTAGAGGATCCCCGGGTACCGA
TB_3_3_C      321 CAACCCAAATCAGATCGCCCATATCGGGTACGTATCGGCTTACCATCGTTTCACTCCTCTAGAGGATCCCCGGGTACCGA
TB_4_3_A      320 CAACCCAAATCAGATCGCCCATATCGGGTACGTATCGGCTTACCATCGTTTCACTCCTCTAGAGGATCCCCGGGTACCGA
TB_4_3_B      321 CAACCCAAATCAGATCGCCCATATCGGGTACGTATCGGCTTACCATCGTTTCACTCCTCTAGAGGATCCCCGGGTACCGA
TB_4_3_C      321 CAACCCAAATCAGATCGCCCATATCGGGTACGTATCGGCTTACCATCGTTTCACTCCTCTAGAGGATCCCCGGGTACCGA

PBADmazF_rev  371 GCTCGAATTCGCTAGCCCAAAAAAACGGGTATGGAGAAACAGTAGAGAGTTGCGATAAAAAGCGTCAGGTAGGATCCGCT
MG_1_1_A      401 GCTCGAATTCGCTAGCCCAAAAAAACGGGTATGGAGAAACAGTAGAGAGTTGCGATAAAAAGCGTCAGGTAGGATCCGCT
MG_1_1_B      400 GCTCGAATTCGCTAGCCCAAAAAAACGGGTATGGAGAAACAGTAGAGAGTTGCGATAAAAAGCGTCAGGTAGGATCCGCT
MG_1_1_C      401 GCTCGAATTCGCTAGCCCAAAAAAACGGGTATGGAGAAACAGTAGAGAGTTGCGATAAAAAGCGTCAGGTAGGATCCGCT
MG_2_1_A      401 GCTCGAATTCGCTAGCCCAAAAAAACGGGTATGGAGAAACAGTAGAGAGTTGCGATAAAAAGCGTCAGGTAGGATCCGCT
MG_2_1_B      337 GCTCGAATTCGCTAGCCCAAAAAAACGGGTATGGAGAAACAGTAGAGAGTTGCGATAAAAAGCGTCAGGTAGGATCCGCT
MG_2_1_C      337 GCTCGAATTCGCTAGCCCAAAAAAACGGGTATGGAGAAACAGTAGAGAGTTGCGATAAAAAGCGTCAGGTAGGATCCGCT
MG_3_1_A      401 GCTCGAATTCGCTAGCCCAAAAAAACGGGTATGGAGAAACAGTAGAGAGTTGCGATAAAAAGCGTCAGGTAGGATCCGCT
MG_3_1_B      375 GCTCGAATTCGCTAGCCCAAAAAAACGGGTATGGAGAAACAGTAGAGAGTTGCGATAAAAAGCGTCAGGTAGGATCCGCT
MG_3_1_C      401 GCTCGAATTCGCTAGCCCAAAAAAACGGGTATGGAGAAACAGTAGAGAGTTGCGATAAAAAGCGTCAGGTAGGATCCGCT
TB_2_3_A      401 GCTCGAATTCGCTAGCCCAAAAAAACGGGTATGGAGAAACAGTAGAGAGTTGCGATAAAAAGCGTCAGGTAGGATCCGCT
TB_2_3_B      399 GCTCGAATTCGCTAGCCCAAAAAAACGGGTATGGAGAAACAGTAGAGAGTTGCGATAAAAAGCGTCAGGTAGGATCCGCT
TB_2_3_C      401 GCTCGAATTCGCTAGCCCAAAAAAACGGGTATGGAGAAACAGTAGAGAGTTGCGATAAAAAGCGTCAGGTAGGATCCGCT
TB_2_3_D      401 GCTCGAATTCGCTAGCCCAAAAAAACGGGTATGGAGAAACAGTAGAGAGTTGCGATAAAAAGCGTCAGGTAGGATCCGCT
TB_3_3_C      401 GCTCGAATTCGCTAGCCCAAAAAAACGGGTATGGAGAAACAGTAGAGAGTTGCGATAAAAAGCGTCAGGTAGGATCCGCT
TB_4_3_A      400 GCTCGAATTCGCTAGCCCAAAAAAACGGGTATGGAGAAACAGTAGAGAGTTGCGATAAAAAGCGTCAGGTAGGATCCGCT
TB_4_3_B      401 GCTCGAATTCGCTAGCCCAAAAAAACGGGTATGGAGAAACAGTAGAGAGTTGCGATAAAAAGCGTCAGGTAGGATCCGCT
TB_4_3_C      401 GCTCGAATTCGCTAGCCCAAAAAAACGGGTATGGAGAAACAGTAGAGAGTTGCGATAAAAAGCGTCAGGTAGGATCCGCT

PBADmazF_rev  451 AATCTTATGGATAAAAATGCTATGGCATAGCAAAGTGTGACGCCGTGCAAATAATCAATGTGGACTTTTCTGCCGTGATT
MG_1_1_A      481 AATCTTATGGATAAAAATGCTATGGCATAGCAAAGTGTGACGCCGTGCAAATAATCAATGTGGACTTTTCTGCCGTGATT
MG_1_1_B      480 AATCTTATGGATAAAAATGCTATGGCATAGCAAAGTGTGACGCCGTGCAAATAATCAATGTGGACTTTTCTGCCGTGATT
MG_1_1_C      481 AATCTTATGGATAAAAATGCTATGGCATAGCAAAGTGTGACGCCGTGCAAATAATCAATGTGGACTTTTCTGCCGTGATT
MG_2_1_A      481 AATCTTATGGATAAAAATGCTATGGCATAGCAAAGTGTGACGCCGTGCAAATAATCAATGTGGACTTTTCTGCCGTGATT
MG_2_1_B      417 AATCTTATGGATAAAAATGCTATGGCATAGCAAAGTGTGACGCCGTGCAAATAATCAATGTGGACTTTTCTGCCGTGATT
MG_2_1_C      417 AATCTTATGGATAAAAATGCTATGGCATAGCAAAGTGTGACGCCGTGCAAATAATCAATGTGGACTTTTCTGCCGTGATT
MG_3_1_A      481 AATCTTATGGATAAAAATGCTATGGCATAGCAAAGTGTGACGCCGTGCAAATAATCAATGTGGACTTTTCTGCCGTGATT
MG_3_1_B      455 AATCTTATGGATAAAAATGCTATGGCATAGCAAAGTGTGACGCCGTGCAAATAATCAATGTGGACTTTTCTGCCGTGATT
MG_3_1_C      481 AATCTTATGGATAAAAATGCTATGGCATAGCAAAGTGTGACGCCGTGCAAATAATCAATGTGGACTTTTCTGCCGTGATT
TB_2_3_A      481 AATCTTATGGATAAAAATGCTATGGCATAGCAAAGTGTGACGCCGTGCAAATAATCAATGTGGACTTTTCTGCCGTGATT
TB_2_3_B      479 AATCTTATGGATAAAAATGCTATGGCATAGCAAAGTGTGACGCCGTGCAAATAATCAATGTGGACTTTTCTGCCGTGATT
TB_2_3_C      481 AATCTTATGGATAAAAATGCTATGGCATAGCAAAGTGTGACGCCGTGCAAATAATCAATGTGGACTTTTCTGCCGTGATT
TB_2_3_D      481 AATCTTATGGATAAAAATGCTATGGCATAGCAAAGTGTGACGCCGTGCAAATAATCAATGTGGACTTTTCTGCCGTGATT
TB_3_3_C      481 AATCTTATGGATAAAAATGCTATGGCATAGCAAAGTGTGACGCCGTGCAAATAATCAATGTGGACTTTTCTGCCGTGATT
TB_4_3_A      480 AATCTTATGGATAAAAATGCTATGGCATAGCAAAGTGTGACGCCGTGCAAATAATCAATGTGGACTTTTCTGCCGTGATT
TB_4_3_B      481 AATCTTATGGATAAAAATGCTATGGCATAGCAAAGTGTGACGCCGTGCAAATAATCAATGTGGACTTTTCTGCCGTGATT
TB_4_3_C      481 AATCTTATGGATAAAAATGCTATGGCATAGCAAAGTGTGACGCCGTGCAAATAATCAATGTGGACTTTTCTGCCGTGATT

PBADmazF_rev  531 ATAGACACTTTTGTTACGCGTTTTTGTCATGGCTTT--------------------------------------------
MG_1_1_A      561 ATAGACACTTTTGTTACGCGTTTTTGTCATGGCTTTGGTCCCGCTTTGTTACAGAATGCTTTTAATAAGCGGGGTTACCG
MG_1_1_B      560 ATAGACACTTTTGTTACGCGTTTTTGTCATGGCTTTGGTCCCGCTTTGTTACAGAATGCTTTTAATAAGCGGGGTTACCG
MG_1_1_C      561 ATAGACACTTTTGTTACGCGTTTTTGTCATGGCTTTGGTCCCGCTTTGTTACAGAATGCTTTTAATAAGCGGGGTTACCG
MG_2_1_A      561 ATAGACACTTTTGTTACGCGTTTTTGTCATGGCTTTGGTCCCGCTTTGTTACAGAATGCTTTTAATAAGCGGGGTTACCG
MG_2_1_B      497 ATAGACACTTTTGTTACGCGTTTTTGTCATGGCTTTGGTCCCGCTTTGTTACAGAATGCTTTTAATAAGCGGGGTTACCG
MG_2_1_C      497 ATAGACACTTTTGTTACGCGTTTTTGTCATGGCTTTGGTCCCGCTTTGTTACAGAATGCTTTTAATAAGCGGGGTTACCG
MG_3_1_A      561 ATAGACACTTTTGTTACGCGTTTTTGTCATGGCTTTGGTCCCGCTTTGTTACAGAATGCTTTTAATAAGCGGGGTTACCG
MG_3_1_B      535 ATAGACACTTTTGTTACGCGTTTTTGTCATGGCTTTGGTCCCGCTTTGTTACAGAATGCTTTTAATAAGCGGGGTTACCG
MG_3_1_C      561 ATAGACACTTTTGTTACGCGTTTTTGTCATGGCTTTGGTCCCGCTTTGTTACAGAATGCTTTTAATAAGCGGGGTTACCG
TB_2_3_A      561 ATAGACACTTTTGTTACGCGTTTTTGTCATGGCTTTGGTCCCGCTTTGTTACAGAATGCTTTTAATAAGCGGGGTTACCG
TB_2_3_B      559 ATAGACACTTTTGTTACGCGTTTTTGTCATGGCTTTGGTCCCGCTTTGTTACAGAATGCTTTTAATAAGCGGGGTTACCG
TB_2_3_C      561 ATAGACACTTTTGTTACGCGTTTTTGTCATGGCTTTGGTCCCGCTTTGTTACAGAATGCTTTTAATAAGCGGGGTTACCG
TB_2_3_D      561 ATAGACACTTTTGTTACGCGTTTTTGTCATGGCTTTGGTCCCGCTTTGTTACAGAATGCTTTTAATAAGCGGGGTTACCG
TB_3_3_C      561 ATAGACACTTTTGTTACGCGTTTTTGTCATGGCTTTGGTCCCGCTTTGTTACAGAATGCTTTTAATAAGCGGGGTTACCG
TB_4_3_A      560 ATAGACACTTTTGTTACGCGTTTTTGTCATGGCTTTGGTCCCGCTTTGTTACAGAATGCTTTTAATAAGCGGGGTTACCG
TB_4_3_B      561 ATAGACACTTTTGTTACGCGTTTTTGTCATGGCTTTGGTCCCGCTTTGTTACAGAATGCTTTTAATAAGCGGGGTTACCG
TB_4_3_C      561 ATAGACACTTTTGTTACGCGTTTTTGTCATGGCTTTGGTCCCGCTTTGTTACAGAATGCTTTTAATAAGCGGGGTTACCG
```

*

All alignments were made with T-Coffee [Notredame et al. 2000], and visualized with BoxShade [Hofmann and Baron 1996].

Sequence S2. Sequence alignment of P_{BAD}-*mazF*ΔACA with sequenced plasmids from single colonies. 6 hours after arabinose induction, dilution series of cultures of strain TB212 pBAD-*mazF*ΔACA were plated and incubated overnight at 37°C. Colonies were counted and these results are reported in Figure 3B. Colonies 'A', 'B', 'D' were then picked and inoculated overnight in LB supplemented with 15 µg/ml chloramphenicol, and mini-prep was performed the following day. Colonies 'C' were formed on the plates after additional overnight incubation at room temperature. Samples are named as Plasmid-#Replicate-#DilutionSeriesPlate-Colony, and sequenced by using standard reverse primer BAD-HisA-R GTTTTATCAGACCGCTTCTG (LGC Genomics). A single mutation was recorded in the multiple cloning site, between SmaI and XbaI sites, in the sample ACA_2_3_A.

```
PBAD-mazFdACA_r    1 CTTCTCTCATCCGCCAAAACAGCCAAGCTTCTACCCAATCAGTACGTTAATTTTGGCTTTAATGAGTTGCAGTTCCTCTG
ACA_1_3_B          1 ----------------------------------------------------------------AGTTGCAGTTCCTCTG
ACA_1_3_C          1 ----------------------------------------------------------------AGTTGCAGTTCCTCTG
ACA_1_3_D          1 CTTCTCTCATCCGCCAAAACAGCCAAGCTTCTACCCAATCAGTACGTTAATTTTGGCTTTAATGAGTTGCAGTTCCTCTG
ACA_2_3_A          1 -TTCTCTCATCCGCCAAAACAGCCAAGCTTCTACCCAATCAGTACGTTAATTTTGGCTTTAATGAGTTGCAGTTCCTCTG
ACA_2_3_B          1 CTTCTCTCATCCGCCAAAACAGCCAAGCTTCTACCCAATCAGTACGTTAATTTTGGCTTTAATGAGTTGCAGTTCCTCTG
ACA_2_3_C          1 -TTCTCTCATCCGCCAAAACAGCCAAGCTTCTACCCAATCAGTACGTTAATTTTGGCTTTAATGAGTTGCAGTTCCTCTG
ACA_4_3_C          1 CTTCTCTCATCCGCCAAAACAGCCAAGCTTCTACCCAATCAGTACGTTAATTTTGGCTTTAATGAGTTGCAGTTCCTCTG
ACA_4_3_D          1 CTTCTCTCATCCGCCAAAACAGCCAAGCTTCTACCCAATCAGTACGTTAATTTTGGCTTTAATGAGTTGCAGTTCCTCTG

PBAD-mazFdACA_r   81 GGGCAACGGTTCCTTTCTTCGTTGCTCCTCTTGCCCGCCAGGCGATACTTTTTACCTGATCAGCTAACGCTACGCCATCA
ACA_1_3_B         17 GGGCAACGGTTCCTTTCTTCGTTGCTCCTCTTGCCCGCCAGGCGATACTTTTTACCTGATCAGCTAACGCTACGCCATCA
ACA_1_3_C         17 GGGCAACGGTTCCTTTCTTCGTTGCTCCTCTTGCCCGCCAGGCGATACTTTTTACCTGATCAGCTAACGCTACGCCATCA
ACA_1_3_D         81 GGGCAACGGTTCCTTTCTTCGTTGCTCCTCTTGCCCGCCAGGCGATACTTTTTACCTGATCAGCTAACGCTACGCCATCA
ACA_2_3_A         80 GGGCAACGGTTCCTTTCTTCGTTGCTCCTCTTGCCCGCCAGGCGATACTTTTTACCTGATCAGCTAACGCTACGCCATCA
ACA_2_3_B         81 GGGCAACGGTTCCTTTCTTCGTTGCTCCTCTTGCCCGCCAGGCGATACTTTTTACCTGATCAGCTAACGCTACGCCATCA
ACA_2_3_C         80 GGGCAACGGTTCCTTTCTTCGTTGCTCCTCTTGCCCGCCAGGCGATACTTTTTACCTGATCAGCTAACGCTACGCCATCA
ACA_4_3_C         81 GGGCAACGGTTCCTTTCTTCGTTGCTCCTCTTGCCCGCCAGGCGATACTTTTTACCTGATCAGCTAACGCTACGCCATCA
ACA_4_3_D         81 GGGCAACGGTTCCTTTCTTCGTTGCTCCTCTTGCCCGCCAGGCGATACTTTTTACCTGATCAGCTAACGCTACGCCATCA

PBAD-mazFdACA_r  161 CGTTCCTGACCGGATAAAACAACTTCGAACGGATATCCTTTTGATTGCGTGGTACAAGGAACACACAGACACATACCGGT
ACA_1_3_B         97 CGTTCCTGACCGGATAAAACAACTTCGAACGGATATCCTTTTGATTGCGTGGTACAAGGAACACACAGACACATACCGGT
ACA_1_3_C         97 CGTTCCTGACCGGATAAAACAACTTCGAACGGATATCCTTTTGATTGCGTGGTACAAGGAACACACAGACACATACCGGT
ACA_1_3_D        161 CGTTCCTGACCGGATAAAACAACTTCGAACGGATATCCTTTTGATTGCGTGGTACAAGGAACACACAGACACATACCGGT
ACA_2_3_A        160 CGTTCCTGACCGGATAAAACAACTTCGAACGGATATCCTTTTGATTGCGTGGTACAAGGAACACACAGACACATACCGGT
ACA_2_3_B        161 CGTTCCTGACCGGATAAAACAACTTCGAACGGATATCCTTTTGATTGCGTGGTACAAGGAACACACAGACACATACCGGT
ACA_2_3_C        160 CGTTCCTGACCGGATAAAACAACTTCGAACGGATATCCTTTTGATTGCGTGGTACAAGGAACACACAGACACATACCGGT
ACA_4_3_C        161 CGTTCCTGACCGGATAAAACAACTTCGAACGGATATCCTTTTGATTGCGTGGTACAAGGAACACACAGACACATACCGGT
ACA_4_3_D        161 CGTTCCTGACCGGATAAAACAACTTCGAACGGATATCCTTTTGATTGCGTGGTACAAGGAACACACAGACACATACCGGT

PBAD-mazFdACA_r  241 TTTATTATTATACATGAAAGGACTCAGGACAACAGCTGGACGATGGCCAGCTTGCTCGCTACCTTTGGTCGGGTCAAAAT
ACA_1_3_B        177 TTTATTATTATACATGAAAGGACTCAGGACAACAGCTGGACGATGGCCAGCTTGCTCGCTACCTTTGGTCGGGTCAAAAT
ACA_1_3_C        177 TTTATTATTATACATGAAAGGACTCAGGACAACAGCTGGACGATGGCCAGCTTGCTCGCTACCTTTGGTCGGGTCAAAAT
ACA_1_3_D        241 TTTATTATTATACATGAAAGGACTCAGGACAACAGCTGGACGATGGCCAGCTTGCTCGCTACCTTTGGTCGGGTCAAAAT
ACA_2_3_A        240 TTTATTATTATACATGAAAGGACTCAGGACAACAGCTGGACGATGGCCAGCTTGCTCGCTACCTTTGGTCGGGTCAAAAT
ACA_2_3_B        241 TTTATTATTATACATGAAAGGACTCAGGACAACAGCTGGACGATGGCCAGCTTGCTCGCTACCTTTGGTCGGGTCAAAAT
ACA_2_3_C        240 TTTATTATTATACATGAAAGGACTCAGGACAACAGCTGGACGATGGCCAGCTTGCTCGCTACCTTTGGTCGGGTCAAAAT
ACA_4_3_C        241 TTTATTATTATACATGAAAGGACTCAGGACAACAGCTGGACGATGGCCAGCTTGCTCGCTACCTTTGGTCGGGTCAAAAT
ACA_4_3_D        241 TTTATTATTATACATGAAAGGACTCAGGACAACAGCTGGACGATGGCCAGCTTGCTCGCTACCTTTGGTCGGGTCAAAAT

PBAD-mazFdACA_r  321 CAACCCAAATCAGATCGCCCATATCGGGTACGTATCGGCTTACCATCGTTTCACTCCTCTAGAGGATCCCCGGGTACCGA
ACA_1_3_B        257 CAACCCAAATCAGATCGCCCATATCGGGTACGTATCGGCTTACCATCGTTTCACTCCTCTAGAGGATCCCCGGGTACCGA
ACA_1_3_C        257 CAACCCAAATCAGATCGCCCATATCGGGTACGTATCGGCTTACCATCGTTTCACTCCTCTAGAGGATCCCCGGGTACCGA
ACA_1_3_D        321 CAACCCAAATCAGATCGCCCATATCGGGTACGTATCGGCTTACCATCGTTTCACTCCTCTAGAGGATCCCCGGGTACCGA
ACA_2_3_A        320 CAACCCAAATCAGATCGCCCATATCGGGTACGTATCGGCTTACCATCGTTTCACTCCTCTAGAGGAGCCCCGGGTACCGA
ACA_2_3_B        321 CAACCCAAATCAGATCGCCCATATCGGGTACGTATCGGCTTACCATCGTTTCACTCCTCTAGAGGATCCCCGGGTACCGA
ACA_2_3_C        320 CAACCCAAATCAGATCGCCCATATCGGGTACGTATCGGCTTACCATCGTTTCACTCCTCTAGAGGATCCCCGGGTACCGA
ACA_4_3_C        321 CAACCCAAATCAGATCGCCCATATCGGGTACGTATCGGCTTACCATCGTTTCACTCCTCTAGAGGATCCCCGGGTACCGA
ACA_4_3_D        321 CAACCCAAATCAGATCGCCCATATCGGGTACGTATCGGCTTACCATCGTTTCACTCCTCTAGAGGATCCCCGGGTACCGA

PBAD-mazFdACA_r  401 GCTCGAATTCGCTAGCCCAAAAAAACGGGTATGGAGAAACAGTAGAGAGTTGCGATAAAAAGCGTCAGGTAGGATCCGCT
ACA_1_3_B        337 GCTCGAATTCGCTAGCCCAAAAAAACGGGTATGGAGAAACAGTAGAGAGTTGCGATAAAAAGCGTCAGGTAGGATCCGCT
ACA_1_3_C        337 GCTCGAATTCGCTAGCCCAAAAAAACGGGTATGGAGAAACAGTAGAGAGTTGCGATAAAAAGCGTCAGGTAGGATCCGCT
ACA_1_3_D        401 GCTCGAATTCGCTAGCCCAAAAAAACGGGTATGGAGAAACAGTAGAGAGTTGCGATAAAAAGCGTCAGGTAGGATCCGCT
ACA_2_3_A        400 GCTCGAATTCGCTAGCCCAAAAAAACGGGTATGGAGAAACAGTAGAGAGTTGCGATAAAAAGCGTCAGGTAGGATCCGCT
ACA_2_3_B        401 GCTCGAATTCGCTAGCCCAAAAAAACGGGTATGGAGAAACAGTAGAGAGTTGCGATAAAAAGCGTCAGGTAGGATCCGCT
ACA_2_3_C        400 GCTCGAATTCGCTAGCCCAAAAAAACGGGTATGGAGAAACAGTAGAGAGTTGCGATAAAAAGCGTCAGGTAGGATCCGCT
ACA_4_3_C        401 GCTCGAATTCGCTAGCCCAAAAAAACGGGTATGGAGAAACAGTAGAGAGTTGCGATAAAAAGCGTCAGGTAGGATCCGCT
ACA_4_3_D        401 GCTCGAATTCGCTAGCCCAAAAAAACGGGTATGGAGAAACAGTAGAGAGTTGCGATAAAAAGCGTCAGGTAGGATCCGCT

PBAD-mazFdACA_r  481 AATCTTATGGATAAAAATGCTATGGCATAGCAAAGTGTGACGCCGTGCAAATAATCAATGTGGACTTTTCTGCCGTGATT
ACA_1_3_B        417 AATCTTATGGATAAAAATGCTATGGCATAGCAAAGTGTGACGCCGTGCAAATAATCAATGTGGACTTTTCTGCCGTGATT
ACA_1_3_C        417 AATCTTATGGATAAAAATGCTATGGCATAGCAAAGTGTGACGCCGTGCAAATAATCAATGTGGACTTTTCTGCCGTGATT
ACA_1_3_D        481 AATCTTATGGATAAAAATGCTATGGCATAGCAAAGTGTGACGCCGTGCAAATAATCAATGTGGACTTTTCTGCCGTGATT
ACA_2_3_A        480 AATCTTATGGATAAAAATGCTATGGCATAGCAAAGTGTGACGCCGTGCAAATAATCAATGTGGACTTTTCTGCCGTGATT
ACA_2_3_B        481 AATCTTATGGATAAAAATGCTATGGCATAGCAAAGTGTGACGCCGTGCAAATAATCAATGTGGACTTTTCTGCCGTGATT
ACA_2_3_C        480 AATCTTATGGATAAAAATGCTATGGCATAGCAAAGTGTGACGCCGTGCAAATAATCAATGTGGACTTTTCTGCCGTGATT
ACA_4_3_C        481 AATCTTATGGATAAAAATGCTATGGCATAGCAAAGTGTGACGCCGTGCAAATAATCAATGTGGACTTTTCTGCCGTGATT
ACA_4_3_D        481 AATCTTATGGATAAAAATGCTATGGCATAGCAAAGTGTGACGCCGTGCAAATAATCAATGTGGACTTTTCTGCCGTGATT

PBAD-mazFdACA_r  561 ATAGACACTTTTGTTACGCGTTTTTGTCATGGCTTT-----------------------------------
ACA_1_3_B        497 ATAGACACTTTTGTTACGCGTTTTTGTCATGGCTTTGGTCCCGCTTTGTTACAGAATGCTTTTAATAAGCGG
ACA_1_3_C        497 ATAGACACTTTTGTTACGCGTTTTTGTCATGGCTTTGGTCCCGCTTTGTTACAGAATGCTTTTAATAAGCGG
ACA_1_3_D        561 ATAGACACTTTTGTTACGCGTTTTTGTCATGGCTTTGGTCCCGCTTTGTTACAGAATGCTTTTAATAAGCGG
ACA_2_3_A        560 ATAGACACTTTTGTTACGCGTTTTTGTCATGGCTTTGGTCCCGCTTTGTTACAGAATGCTTTTAATAAGCGG
ACA_2_3_B        561 ATAGACACTTTTGTTACGCGTTTTTGTCATGGCTTTGGTCCCGCTTTGTTACAGAATGCTTTTAATAAGCGG
ACA_2_3_C        560 ATAGACACTTTTGTTACGCGTTTTTGTCATGGCTTTGGTCCCGCTTTGTTACAGAATGCTTTTAATAAGCGG
ACA_4_3_C        561 ATAGACACTTTTGTTACGCGTTTTTGTCATGGCTTTGGTCCCGCTTTGTTACAGAATGCTTTTAATAAGCGG
ACA_4_3_D        561 ATAGACACTTTTGTTACGCGTTTTTGTCATGGCTTTGGTCCCGCTTTGTTACAGAATGCTTTTAATAAGCGG
```

Supplementary Methods

Plate-reader experiments

Arabinose induction. Overnight cultures were diluted 1 to 1000, and 190 µl of the diluted cultures were put into a 96-well plate. There were two technical replicates per each overnight culture. After 2.5 hours of exponential growth, 10 µl of arabinose-stock was added to one part of exponential cultures, final concentration 0.1% Ara. In the other part of technical replicates, 10 µl of sterile distilled water was added. Growth of the cultures with the final volume of 200 µl was recorded for additional 6 hours.

Recovery after arabinose induction. Exponentially growing cultures were divided into two flasks; in the first part 0.1% arabinose was added to induce *mazF* expression, in the second part 0.1% glucose was added. 6 hours after arabinose induction, the cultures were washed. Induced cultures were washed twice with 1x PBS, uninduced cultures were washed once. The cultures were then resuspended in 1 ml of pre-warmed fresh media supplemented with 10 mM glucose (instead of maltose), and a series of dilutions was made: 10^{-1}, 10^{-2}, and 10^{-3}. 200 µl of each dilution series were put into a 96-well plate and the recovery growth was monitored overnight. For the cultures shown in Figure 1C, the starting population density was adjusted to the same order of magnitude, A_{600}= 0.001-0.008.

Colony count and plasmid sequencing

Overnight cultures were prepared from single colonies belonging to strains MG1655 (wild-type), LVM100 (Δ5) and LVM101 (Δ*mazEF*) harboring pBAD-*mazF*, as well as a BW27784-derived strain TB212 harboring pBAD-*mazF* or pBAD-*mazF*ΔACA. Four overnight cultures per genotype were grown in a final volume of 4 ml, with addition of 0.1% Glc. Overnight cultures were diluted 1 to 1000 in 14 ml of fresh media, and grown for 2 hours 15 minutes until aliquots were taken. 100 µl of exponential cultures from serial dilutions 10^{-3}-10^{-5} were spread on LB agar plates with chloramphenicol. Exponential cultures were then separated in two flasks; in one flask we added arabinose to final concentration of 0.1%, while the other served as a control. After 6 hours, aliquots were taken from stressed and control cultures. 100 µl of stressed cultures from serial dilutions 10^{-1}-10^{-6}, and 100 µl of control cultures from serial dilutions 10^{-6}-10^{-8} were spread on LB plates with chloramphenicol. All plates were incubated for 15 hours at 37°C before calculating the number of colony forming units (CFU).

We picked 1-3 colonies per plate of strains MG1655 pBAD-*mazF* (plates with dilution 10^{-1}), TB212 pBAD-*mazF* and TB212 pBAD-*mazF*ΔACA (plates with dilution 10^{-3}), from 3 replicate plates containing 6 hours-induced cultures. These colonies were of different sizes. The plates were further incubated at room temperature for additional 9 hours (incubation time of 24 hours in total), and one newly formed small colony per plate was picked. All colonies were inoculated overnight in LB medium with chloramphenicol, and mini-prep (Zymo) was preformed the following day. The prepped plasmids were sequenced with the standard primer BAD-HisA-R 5'-GTTTTATCAGACCGCTTCTG-3' (LGC Genomics) to investigate the P_{BAD}-*mazF* region of the plasmids.

Flow cytometry

Fluorescence settings. Fluorescence intensity was measured with the "height" ("-H") settings: FITC-H for GFP detection, and PE-Texas Red-H for mCherry detection. In contrast, using "area" ("-A") settings measures total fluorescence of each recorded event.
Data export. Raw data were exported from FlowJo software version 8.8.7 (Tree Star, Inc.) into the custom *R* script, similar to one used in [Silander et al. 2012]. Fluorescence histograms were made in FlowJo, and normalized by the maximum fluorescence value of the measured sample.

Quantitative reverse transcription PCR (RT-qPCR)

Overnight cultures of the strain MG1655 Δ*mazF* harboring either pBAD-*mazF* or pBAD-*mazF*ΔACA were diluted in 50 ml of LB medium with chloramphenicol, and their growth was monitored by measuring OD_{600}. At OD_{600}= 0.4, *mazF* expression was induced by addition of L-arabinose to the final concentration of 0.2%. 1 hour after induction, transcription was arrested by addition of 50 µg/ml of rifampicin [Selinger et al. 2003]. 500 µl samples were collected immediately before as well as 2.5, 5, 10, and 20 min after rifampicin addition. Samples were immediately stabilized by resuspension in 2 volumes of RNAprotect® Bacteria (QIAgen). RNA was extracted on RNeasy columns (QIAgen) according to the manufacturer instructions (RNAprotect® Bacteria kit, protocol 1, enzymatic lysis). 5 µg of sample were treated with TURBO DNA-free™ Kit (Thermo Scientific), for removal of contaminating DNA. cDNA libraries were prepared with iScript™ cDNA Synthesis Kit (Biorad); reactions without Reverse Transcriptase enzyme were included to assess presence of contaminating DNA. Quantitative reverse transcription PCR (RT-qPCR) was done using probes with 5'-FAM and 3'-BHQ1 (Eurofins) and iTaq Universal Probes Supermix (Biorad). 8 µl primer mix (0.5 µM of each forward and reverse primer, and 0.1 µM probe) were mixed with 2 µl cDNA sample and 10 µl iTaq Supermix and run with a standard protocol on a Biorad CFX96 real-time system. Two sets containing two primers and a probe were designed for specific detection of an ACA-free (A probe) and ACA-containing region (B probe) of the *mazF* transcript, and one set was designed for specific detection of the *cysG* transcript that served as housekeeping control (for probes and primers see Table S2). Each biological replicate was measured in technical triplicates, and each technical replicate was run with the three sets. Cq values of biological replicates were averaged over three technical replicates. ΔCq values were calculated for each biological replicate using *cysG* as the reference [Venturelli et al. 2017]. Relative transcript abundance was calculated as $2^{-\Delta Cq}$, and normalized to the initial measurement at time point t= 0 min. To determine mRNA half-life, $2^{-\Delta Cq}$ values were $\log_{10}$-transformed, and the degradation constant k_d was calculated as the slope of the linear regression line through five time points, with R^2 values of at least 0.9. Finally, mRNA half-life was calculated as $-\ln2/k_d$.

Microfluidic operation

Microfluidic wafers and devices ("mother machine" from [Wang et al. 2010]) were constructed as described previously [Arnoldini et al. 2014, Bergmiller et al. 2017] and were a gift from Daniel J. Kiviet, Eawag and ETH Zurich, Switzerland. In short, growth channels were 1.2 to 1.4 mm wide, 1.1 mm high and 24 mm to 25 mm long. Microfluidic devices were assembled following previously published protocols [Arnoldini et al. 2014, Bergmiller et al. 2017]. Briefly, polydimethylsiloxane (PDMS, Sylgard) was mixed 1:10 with polymerization agent, poured onto the wafer, degassed and

cured overnight at 85°C. Devices were cut out, and outlets were punched using 22ga luer stubs (Instech). Devices were bonded to glass cover slips (24 mm x 50 mm, 0.17 +/- 0.005 mm thickness, from Carl Roth) after exposure to air plasma and incubated for 1 hour on a heat plate at 100°C. Bacterial cultures were grown to a density of approximately OD_{600}= 0.5, in the appropriate medium containing 0.01% Tween to minimize attachment of bacteria to surfaces and to improve loading. Before loading, we concentrated cultures around 50 fold by centrifugation, and injected them into microfluidic devices that were wetted with growth medium beforehand. After approximately 1 hour, we connected thin BPE-T50 (Instech) tubing via 22ga pins to the device for operation, and pumped growth medium with a constant rate of 2 ml/h using syringe pumps (NE-1000). To switch between arabinose-containing growth media, we connected two syringe pumps using a y-connector, and switched between the two pumps using a in-house written script (LabView).

Statistical analysis

Statistics was done in *R*, SPSS, Microsoft Excel and Matlab. Error bars in all graphs present standard error of the mean; except transcript stability depicted in Figure 4E - error bars present standard deviation. All fluorescence data was analyzed in the $\log_{10}$ scale, as the fluorescence profile of exponentially growing culture follows log-normal distribution. Squared coefficient of variation SCV was used as a measure of variation in the reporter fluorescence (which is suitable for instances when variance is small relative to the mean level). When fluorescence distributions were bi- or multimodal we calculated Shannon entropy as a measure of variation, by using package 'entropy' in *R* (maximum likelihood method, 40 bins, range 0.4-4.0). Coefficient of variation CV was used as a measure of variation in the elongation rate in microfluidics experiments. We used non-parametric Spearman's two-tailed test to assess significant correlations between datasets, and calculated R^2 to infer how well is the variation in mCherry fluorescence explained by the variation in cell elongation rate. To evaluate whether the datasets are drawn from the same distribution and have the same medians, we applied Kolmogorov-Smirnov test and Mann-Whitney U test, respectively. We used non-parametric Sign test for related samples to assess the differences in phenotypic traits in the cells before and after arabinose induction. To evaluate differences in flow cytometry datasets we used Student's *t*-tests, 2-tailed, paired (induced vs. uninduced cultures) or heteroscedastic (different strains).

Reporters' construction

The sequence mazF_ACAless was designed according to [Suzuki et al. 2007], ordered from IDT (Integrated DNA Technologies, Inc.), and cloned via XbaI/HindIII into plasmid pBAD-*mazF*.

>construct_*mazF*ΔACA
TCTAGAGGAGTGAAACGatgGTAAGCCGATACGTACCCGATATGGGCGATCTGATTTGGGTTGATTTTGACCCGA
CCAAAGGTAGCGAGCAAGCTGGCCATCGTCCAGCTGTTGTCCTGAGTCCTTTCATGTATAATAATAAAACCGGTA
TGTGTCTGTGTGTTCCTTGTACCACGCAATCAAAAGGATATCCGTTCGAAGTTGTTTTATCCGGTCAGGAACGTG
ATGGCGTAGCGTTAGCTGATCAGGTAAAAAGTATCGCCTGGCGGGCAAGAGGAGCAACGAAGAAAGGAACCGTTG
CCCCAGAGGAACTGCAACTCATTAAAGCCAAAATTAACGTACTGATTGGGtagAAGCTT

Plasmid pBAD-*mazF*$_{E24A}$ was made according to [Tripathi et al. 2014]; MazF(E24A) mutation was introduced on pBAD-*mazF* through inverse PCR using primers J23 MazFE24A_fwd (GTAGCGCGCAAGCTGGACATC) and K23 MazFE24A_rev (CTTTTGTCGGGTCAAAATCAACC).

'NN-cat' strains were made by using CRIM method described in [Haldimann and Wanner 2001]: different reporter constructs were cloned into modified CRIM plasmid pAH120-frt-cat [Haldimann and Wanner 2001], and inserted into its respective attachment site *att*λ. The chloramphenicol resistance marker was removed by using the site-specific Flp-recombinase [Cherepanov and Wackernagel 1995].

>**NN204**_P$_{BAD}$-*mazF*
CTGCAGACTCGAGcgccattcagagaagaaaccaattgtccatattgcatcagacattgccgtcactgcgtcttt
tactggctcttctcgctaaccaaaccggtaaccccgcttattaaaagcattctgtaacaaagcgggaccaaagcc
atgacaaaaacgcgtaacaaaagtgtctataatcacggcagaaaagtccacattgattatttgcacggcgtcaca
ctttgctatgccatagcatttttatccataagattagcggatcctacctgacgctttttatcgcaactctctact
gtttctccatacccgtttttttgggctagcgaattcgagctcggtacccggggatcctctaga**GGAGTGAAACG**a
tgGTAAGCCGATACGTACCCGATATGGGCGATCTGATTTGGGTTGATTTTGACCCGACAAAAGGTAGCGAGCAAG
CTGGACATCGTCCAGCTGTTGTCCTGAGTCCTTTCATGTACAACAACAAAACAGGTATGTGTCTGTGTGTTCCTT
GTACAACGCAATCAAAAGGATATCCGTTCGAAGTTGTTTTATCCGGTCAGGAACGTGATGGCGTAGCGTTAGCTG
ATCAGGTAAAAAGTATCGCCTGGCGGGCAAGAGGAGCAACGAAGAAAGGAACAGTTGCCCCAGAGGAATTACAAC
TCATTAAAGCCAAAATTAACGTACTGATTGGGtagaagcttg

Ribosome binding site: **GGAGTGAAACG**
PCR-amplification of the P$_{BAD}$-*mazF* template from plasmid pBAD-*mazF*
S33 Fwd_PstXho_pBAD CACCTGCAGACTCGAGCGCCATTCAGAGAAGAAACCAA
T33 Rev_BglHind_mazF CACAGATCTCAAGCTTCTACCCAATCAGTACG

>**NN209**_P$_{BAD}$-Em*gfp*ΔACA
CTGCAGACTCGAGcgccattcagagaagaaaccaattgtccatattgcatcagacattgccgtcactgcgtcttt
tactggctcttctcgctaaccaaaccggtaaccccgcttattaaaagcattctgtaacaaagcgggaccaaagcc
atgacaaaaacgcgtaacaaaagtgtctataatcacggcagaaaagtccacattgattatttgcacggcgtcaca
ctttgctatgccatagcatttttatccataagattagcggatcctacctgacgctttttatcgcaactctctact
gtttctccatacccgtttttttgggctagcgaattcgagctcggtacccggggatcctctaga**GGAGTGAAACG**A
TGGTGAG...AAGTAAGAAGCTTGGAATTC

PCR-amplification of the Em*gfp*ΔACA template from plasmid pMS2_612
U33 Fwd_KpnBAD33_MSgfp
CACGGTACCcggggatcctctaga**GGAGTGAAACG**ATGGTGAGCAAGGGCGAG
V33 Rev_EcoHind_MSgfp CACGAATTCCAAGCTTCTTACTTATACAGCTCGTCCATGC
D34 Rev_Bgl_MSgfp CACAGATCTCTTACTTATACAGCTCGTC

Model Description

The parameter set for the *mazEF* toxin-antitoxin module was built up similarly as the parameter sets for the *phd/doc*, *ccdAB* and *relBE* toxin-antitoxin modules in [Gelens et al. 2013]. mRNA transcription only takes place when the promoter/operator region is unbound. This promoter/operator region is assumed to contain three independent binding sites for the antitoxin MazE. The transcription and translation rates are based upon the transcript lengths, and further take into account that MazE and MazF form dimers in solution. To account for the translational coupling, which ensures that the translation rate for the toxin is always lower than that for the antitoxin, the toxin translation rate based on the transcript length was decreased by a factor ten.

The decay rate of the mRNA in the absence of the toxin MazF is based on a half-life of 5.7 minutes *in vivo* [Bernstein et al. 2002]. Cell division is only implicitly included in the model in the decay rate d_c for the toxin and complexes AT and TAT. As the antitoxins are always degraded faster than their cognate toxins, the antitoxin decay rate d_a was fixed as 8 times d_c. Antitoxin degradation in AT and TAT complexes is included in the model and described by the parameter F.

The high affinity interaction between MazE and MazF was studied using Surface Plasmon Resonance (SPR) [Loris et al., unpublished results] and is described by the parameters α_{TH} and ϑ_{TH}. The parameters for the DNA binding by the antitoxin and the toxin-antitoxin complex are based upon the 5 µM dissociation constant found by isothermal titration calorimetry (ITC) for the interaction of MazE with a DNA fragment containing one antitoxin binding site, and the higher affinity found for the toxin-antitoxin complex using electrophoretic mobility shift assays (EMSA) [Zorzini et al. 2015].

When indicated, the cleavage of the *mazEF* mRNA by the toxin MazF was included in the model as

$$d_m \to d_m + (d_{m,max} - d_m)\frac{T^n}{K_T^n + T^n}$$

The cell division times are calculated based on the amount of free toxin in the cell as

$$t_D = (t_{D,0} + (t_{D,max} - t_{D,0})\frac{T^{n_D}}{K_{TD}^{n_D} + T^{n_D}})(1 + Dr)$$

where *r* is a random number taken from the standard normal distribution.

A period of stress was included in the simulations in two different ways: either by increasing the antitoxin degradation rate with a factor 3 or by increasing the toxin production rate with a factor 5.
The models were simulated using a Gillespie algorithm [Gillespie 1977] with core reactions as described in [Gelens et al. 2013] and presented systematically in [Vandervelde et al. 2016], shown schematically in Figure S16.

Supplementary References

Arnoldini M, Vizcarra IA, Peña Miller R, Stocker N, Diard M, et al. (2014) Bistable expression of virulence genes in *Salmonella* leads to the formation of an antibiotic-tolerant subpopulation. PLoS Biol 12(8):e1001928.

Battesti A, Majdalani N, Gottesman S (2011) The RpoS-mediated general stress response in *Escherichia coli*. Annu Rev Microbiol 65: 189-213.

Bergmiller T, Andersson AMC, Tomasek K, Balleza E, Kiviet DJ, et al. (2017) Biased partitioning of the multidrug efflux pump AcrAB-TolC underlies long-lived phenotypic heterogeneity. Science 356(6335): 311-315.

Bernstein JA, Khodursky AB, Lin PH, Lin-Chao S, Cohen SN (2002) Global analysis of mRNA decay and abundance in *Escherichia coli* at single-gene resolution using two-color fluorescent DNA microarrays. Proc Natl Acad Sci U S A 99: 9697-9702.

Blattner FR, Plunkett G, Bloch CA, Perna NT, Burland V, et al. (1997) The complete genome sequence of *Escherichia coli* K-12. Science 277: 1453-1462.

Cherepanov PP, Wackernagel W (1995) Gene disruption in *Escherichia coli*: TcR and KmR cassettes with the option of Flp-catalyzed excision of the antibiotic-resistance determinant. Gene 158: 9-14.

Coleman TM, Wang G, Huang F (2004) Superior 5′ homogeneity of RNA from ATP-initiated transcription under the T7 phi 2.5 promoter. Nucleic Acids Res 32: e14.

Gelens L, Hill L, Vandervelde A, Danckaert J, Loris R (2013) A general model for toxin-antitoxin module dynamics can explain persister cell formation in *E. coli*. PLoS Comput Biol 9(8): e1003190.

Gillespie DT (1977) Exact stochastic simulation of coupled chemical reactions. J Phys Chem 81: 2340-2361.

Haldimann A, Wanner BL (2001) Conditional-replication, integration, excision, and retrieval plasmid-host systems for gene structure-function studies of bacteria. J Bacteriol 183: 6384-6393.

Hofmann K, Baron MD (1996) BOXSHADE 3.21. http://www.ch.embnet.org/software/BOX_form.html

Khlebnikov A, Datsenko KA, Skaug T, Wanner BL, Keasling JD (2001) Homogeneous expression of the P(BAD) promoter in *Escherichia coli* by constitutive expression of the low-affinity high-capacity AraE transporter. Microbiol 147:3241-3247.

Mets T, Lippus M, Schryer D, Liiv A, Kasari V, Paier A, Maiväli Ü, Remme J, Tenson T, Kaldalu N (2017) Toxins MazF and MqsR cleave *Escherichia coli* rRNA precursors at multiple sites. RNA Biol 14(1): 124-135.

Miller JH (1972) Cold Spring Harbor. NY: Cold Spring Harbor Laboratory Press; Experiments in Molecular Genetics.

Müller C, Sokol L, Vesper O, Sauert M, Moll I (2016) Insights into the stress response triggered by kasugamycin in *Escherichia coli*. Antibiotics (Basel) 5(2): 19.

Notredame C, Higgins DG, Heringa J (2000) T-Coffee: A novel method for fast and accurate multiple sequence alignment. J Mol Biol 302: 205-217.

Oron-Gottesman A, Sauert M, Moll I, Engelberg-Kulka H (2016) A stress-induced bias in the reading of the genetic code in *Escherichia coli*. mBio 7(6):e01855-16.

Scott M, Gunderson CW, Mateescu EM, Zhang Z, Hwa T (2010) Interdependence of cell growth and gene expression: origins and consequences. Science 330: 1099-1102.

Sauert M (2015) Selective translation of leaderless mRNAs by specialized ribosomes upon MazF-mediated stress response in *Escherichia coli*. PhD Dissertation, University of Vienna, Center for Molecular Biology, Vienna, Austria.

Selinger DW, Saxena RM, Cheung KJ, Church GM, Rosenow C (2003) Global RNA half-life analysis in *Escherichia coli* reveals positional patterns of transcript degradation. Genome Res 13: 216–223.

Suzuki M, Mao L, Inouye M (2007) Single protein production (SPP) system in *Escherichia coli*. Nat Protoc 2: 1802-1810.

Tripathi A, Dewan PC, Siddique SA, Varadarajan R (2014) MazF induced growth inhibition and persister generation in *Escherichia coli*. J Biol Chem 289: 4191-4205.

Tsilibaris V, Maenhaut-Michel G, Mine N, Van Melderen L (2007) What is the benefit to *Escherichia coli* of having multiple toxin-antitoxin systems in its genome? J Bacteriol 189: 6101-6108.

Vandervelde A, Loris R, Danckaert J, Gelens L (2016) Computational Methods to Model Persistence. In: Michiels J, Fauvart M, editors. Bacterial Persistence: Methods and Protocols. Springer New York: pp.207-240.

Venturelli OS, Tei M, Bauer S, Chan LJ, Petzold CJ, Arkin AP (2017) Programming mRNA decay to modulate synthetic circuit resource allocation. Nat Commun 8: 15128.

Wang P, Robert L, Pelletier J, Dang WL, Taddei F, et al. (2010) Robust growth of *Escherichia coli*. Curr Biol 20: 1099-1103.

Yamaguchi Y, Inouye M (2013) Type II toxin-antitoxin loci: The *mazEF* Family. In: Gerdes K, editor. Prokaryotic Toxin-Antitoxins. Springer, Berlin, Germany: pp. 107-136.

Zorzini V, Buts L, Schrank E, Sterckx YG, Respondek M, et al. (2015) *Escherichia coli* antitoxin MazE as transcription factor: insights into MazE-DNA binding. Nucleic Acids Res 43(2): 1241-1256.
